# Development of Indole Alkaloid-Type Dual Immune Checkpoint Inhibitors Against CTLA-4 and PD-L1 Based on Diversity-Enhanced Extracts

**DOI:** 10.3389/fchem.2021.766107

**Published:** 2021-11-08

**Authors:** Yoshihide Suzuki, Keisuke Ichinohe, Akihiro Sugawara, Shinya Kida, Shinya Murase, Jing Zhang, Osamu Yamada, Toshio Hattori, Yoshiteru Oshima, Haruhisa Kikuchi

**Affiliations:** ^1^ Graduate School of Pharmaceutical Sciences, Tohoku University, Sendai, Japan; ^2^ Research and Development Center, FUSO Pharmaceutical Industries, Ltd., Osaka, Japan; ^3^ Research Institute of Health and Welfare, Kibi International University, Takahashi, Japan; ^4^ Division of Natural Medicines, Faculty of Pharmacy, Keio University, Tokyo, Japan

**Keywords:** immune checkpoint inhibitors, natural products, indole, PD-L1, CTLA-4, diversity-enhanced extracts

## Abstract

Cancer immunotherapy involves the use of the immune system for cancer treatment. Recently, immune checkpoint-blocking antibodies have become integral for the treatment of some cancers. However, small molecules exhibit advantages over monoclonal antibody drugs, such as cell penetration, long half-life, and low manufacturing costs, and the possibility of oral administration. Thus, it is imperative to develop small-molecule immune checkpoint inhibitors. Previously, we have screened a library of synthetic indole-alkaloid-type compounds, which are produced by diversity-enhanced extracts of Japanese cornelian cherry, and reported that an unnatural pentacyclic compound inhibits CTLA-4 gene expression. In this study, immune checkpoint inhibitors with increased potency were developed by introducing substituents and conversion of functional groups based on the unnatural pentacyclic compound. The developed compounds suppressed not only CTLA-4 and PD-L1 gene expression but also protein expression on the cell surface. Their efficacy was not as potent as that of the existing small-molecule immune checkpoint inhibitors, but, to the best of our knowledge, the developed compounds are the first reported dual small-molecule inhibitors of CTLA-4 and PD-L1.

## Introduction

Cancer immunotherapy involves the use of the immune system for cancer treatment. Recently, immune checkpoint inhibitors have become integral for the treatment of some cancers (Pardoll, 2012; Sharma and Allison, 2015; Darvin et al., 2018; Robert, 2020). Immune checkpoints are negative regulators of the immune system, playing roles in autoimmunity prevention and self-tolerance maintenance. Programmed cell death-1 (PD-1) (Wherry, 2011; Schietinger and Greenberg, 2014) and cytotoxic T-lymphocyte-associated protein 4 (CTLA-4) (Wing et al., 2008) are typical immune checkpoint proteins on T cells. Sometimes, cancer cells find ways to use these checkpoints to avoid attack by the immune system. Recently, ipilimumab (Yervoy^®^), an anti-CTLA-4 monoclonal antibody, pembrolizumab (Keytruda^®^), and nivolumab (Opdivo^®^), anti-PD-1 monoclonal antibodies, have been approved. These antibody drugs exhibit clinically significant antitumor responses; however, small molecules exhibit advantages over monoclonal antibody drugs, such as cell penetration, long half-life, and low manufacturing costs, and the possibility of oral administration. Thus, it is imperative to develop small-molecule immune checkpoint inhibitors (Adams et al., 2015; Smith et al., 2019).

Recently, we have proposed the use of “diversity-enhanced extracts” (Kikuchi et al., 2014; Kikuchi et al., 2016; Oshima and Kikuchi, 2018; Kikuchi et al., 2019), an approach for increasing the chemical diversity of natural-product-like compounds *via* the combination of natural product chemistry and diversity-oriented synthesis (Schreiber, 2000; Burke et al., 2003; Burke and Schreiber, 2004). Diversity-enhanced extracts are obtained from multiple chemical reactions that directly form new carbon–carbon bonds in the extracts of natural resources in order to afford diverse natural product–like library-bearing remodeled molecular scaffolds. Several chemical transformations of natural extracts using similar methods have been reported (López et al., 2007; Ramallo et al., 2011; Ramallo et al., 2018; Solís et al., 2019; Salazar et al., 2020) (Kawamura et al., 2011; Kamauchi et al., 2015; Tomohara et al., 2016; Kamauchi et al., 2018; Guo et al., 2021; Sulistyowaty et al., 2021), but most of the methods simply provide compounds in which some functional groups have been transformed. However, we have defined diversity-oriented extracts as those natural extracts formed by reactions that produce multiple diversities similar to that observed in diversity-oriented synthesis, including the formation of new carbon–carbon bonds, modification of molecular scaffolds (Oshima and Kikuchi, 2018), and conversion of functional groups.

As reported previously (Kikuchi et al., 2016), the CTLA-4 gene expression inhibitor **1** was obtained by screening a library of unnatural indole alkaloid-type compounds produced from the diversity-enhanced extracts. In this study, we synthesized derivatives of **1**, which we subsequently evaluated to identify compounds with higher potency. This led to the development of dual immune checkpoint inhibitors against PD-L1 and CTLA-4.

## Results and Discussion

### Synthesis of Indole Ring–Substituted Derivatives Using Diversity-Enhanced Extracts and Their Immune Checkpoint Inhibitory Activity

**FIGURE 1:**
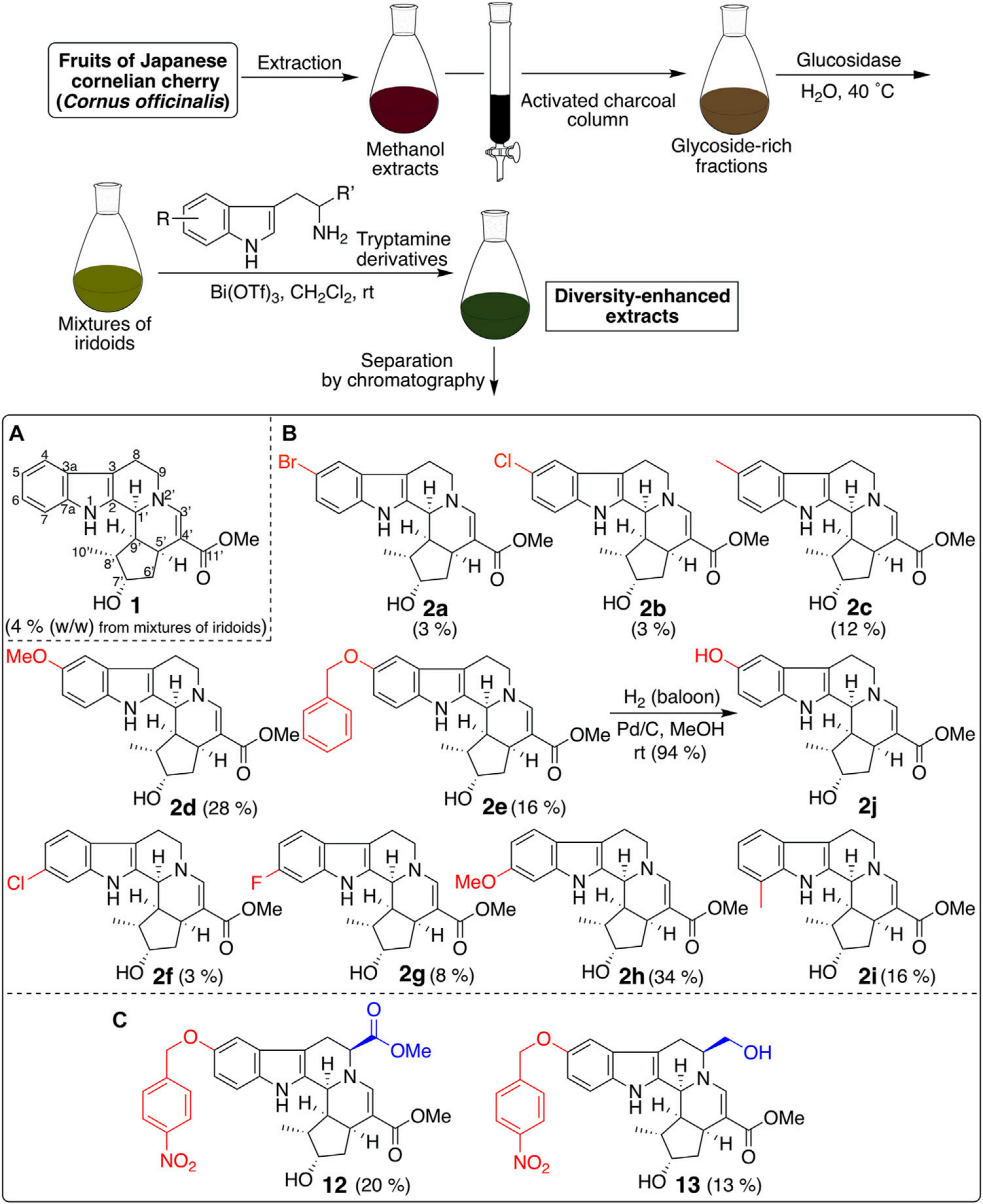
Synthesis of a pentacyclic indole-alkaloid-type compound **1** and its derivatives using the diversity-enhanced extracts of *C. officinalis*.

CTLA-4 expression inhibitor **1** was prepared from the diversity-enhanced extracts of Japanese cornelian cherry, fruits of *Cornus officinalis*, in our previous study (Figure 1A) (Kikuchi et al., 2016). That is, the methanol extracts of *C. officinalis*, which contain various iridoid glucosides, were treated with α-glucosidase to afford mixtures of iridoids. Next, these mixtures were subjected to the Pictet–Spengler reaction with tryptamine to obtain diversity-enhanced extracts containing indole alkaloid–like compounds. Finally, the diversity-enhanced extracts were separated to afford a pentacyclic indole alkaloid-type compound **1** as the major product. Compound **1** moderately suppressed the CTLA-4 gene expression (IC_50_ 49 μM) in reporter gene assays using CTLA-4/luciferase reporter/HEK293 cells. In addition, **1** suppressed PD-L1 gene expression (IC_50_ 57 μM) in reporter gene assays using PD-L1/luciferase reporter/A549 cells. Therefore, **1** is a dual immune checkpoint inhibitor against PD-L1 and CTLA-4, although its effect is weak.

**FIGURE 2:**
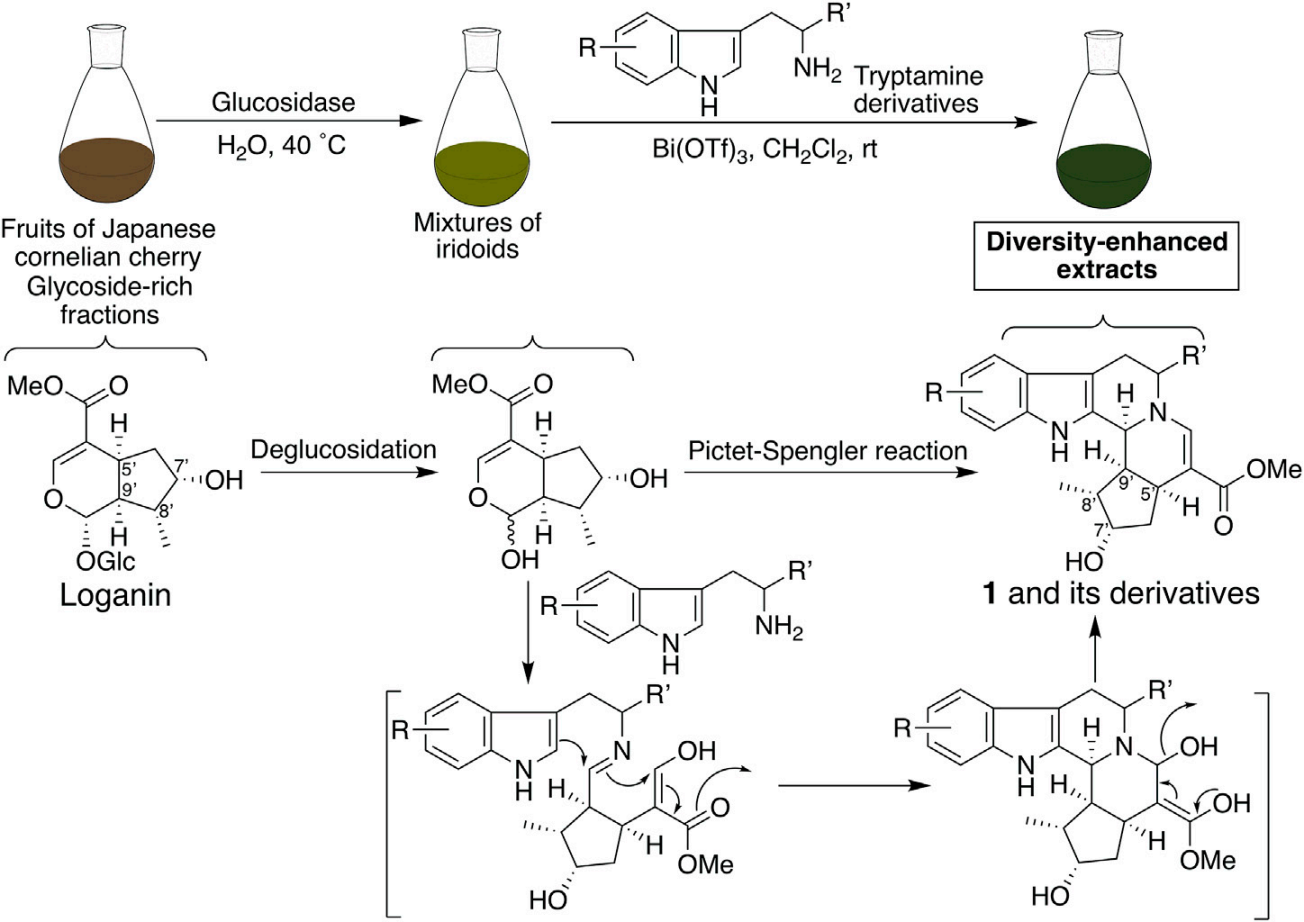
Plausible synthetic pathways for the production of pentacyclic compound **1** and its derivatives using the diversity-enhanced extracts of *C. officinalis*.

Next, derivatives of **1** were synthesized to obtain dual suppressers with higher potency against PD-L1 and CTLA-4. Compound **1** is a conjugate of tryptamine and loganin (Figure 2) (Endo and Taguchi, 1973; Inouye et al., 1974), which is a major iridoid glucoside contained in *C. officinalis*; however, in general, it is difficult to isolate iridoid glucosides. Thus, diversity-enhanced extracts of *C. officinalis* were used to produce derivatives of **1**. Namely, mixtures of iridoids, which were α-glucosidase-treated extracts of *C. officinalis*, were subjected to condensation with commercially available tryptamines bearing several substituents on the indole ring. As a result, 5-bromo (**2a**), 5-chloro (**2b**), 5-methyl (**2c**), 5-methoxy (**2d**), 5-benzyloxy (**2e**), 6-chloro (**2f**), 6-fluoro (**2g**), 6-methoxy (**2h**), and 7-methyl (**2i**) derivatives were obtained (Figure 1B). The hydrogenolysis of a benzyl group of **2e** afforded a 5-hydroxy derivative (**2j**).

Next, effects of **2a**–**2j** with substituents on the indole ring of **1** were examined on the CTLA-4 and PD-L1 expression (Table 1). Compared to **1**, a majority of the compounds exhibited a marginal change. However, 5-benzyloxy compound **2e** exhibited a slightly increased inhibitory activity toward CTLA-4 and PD-L1 expression. Thus, the introduction of a bulky substituent at C-5 of the indole ring is desirable; as a result, further derivatization was conducted on the basis of the compound structure of **2e**.

**TABLE 1:** Immune checkpoint inhibitory effects of the synthesized compounds on the gene expression of CTLA-4 and PD-L1.

Compound	IC_50_ (μM) against CTLA-4^a^	IC_50_ (μM) against PD-L1^b^
1	49	57
2a	>50	>50
2b	36	37
2c	>50	>50
2d	>50	>50
2e	26	31
2f	36	23
2g	>50	>50
2h	>50	41
2i	>50	46
2j	>50	>50
3a	31	47
3b	18	20
3c	26	18
3d	21	21
3e	20	14
5	30	27
6	>50	42
7	39	25
12	18	9.0
13	18	8.7

a On HEK293 cells transfected with pCTLA-4–luciferase.

b On A549 cells transfected with pPD-L1–luciferase.

### Synthesis of Compounds With a Bulky Substituent at the Fifth-Position of the Indole Ring and Their Immune Checkpoint Inhibitory Activity

**FIGURE 3:**
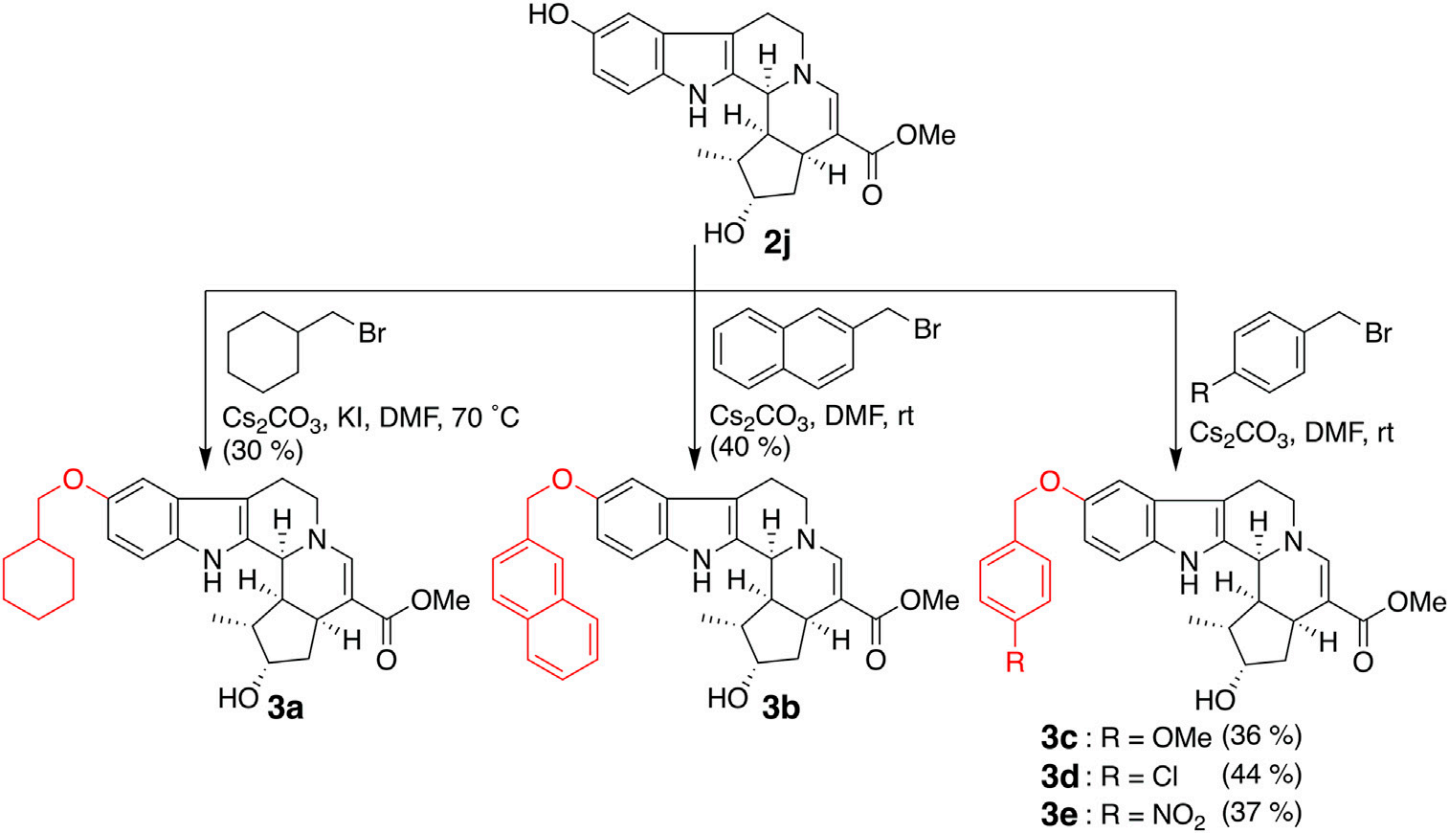
Synthesis of compounds **3a**–**e** by the introduction of bulky substituents.

**FIGURE 4:**
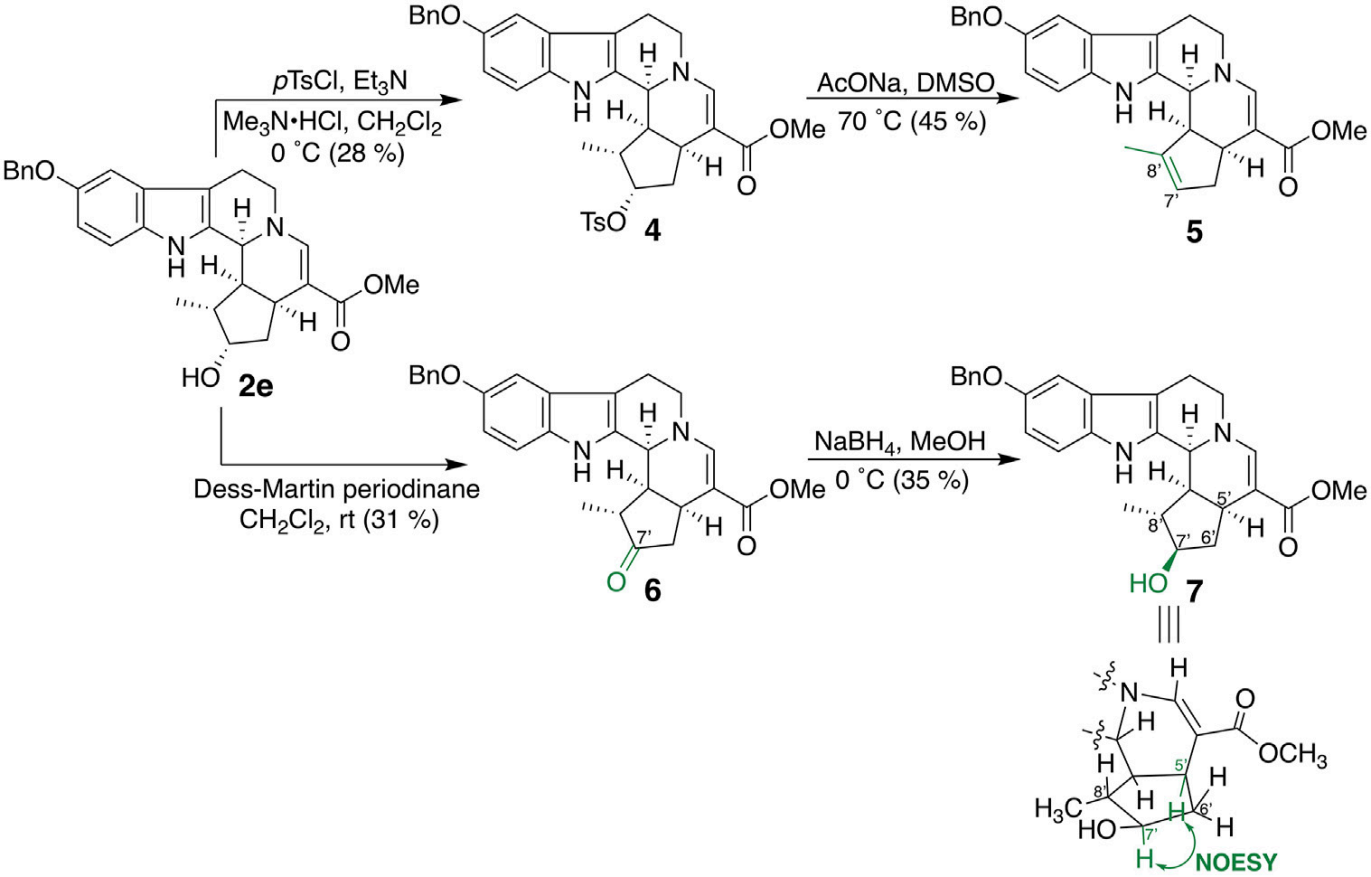
Synthesis of iridoid moiety–modified compounds **5**–**7**.

Bulky substituents equivalent to or greater than the benzyl group and cyclohexylmethoxy (**3a**) and 2-naphthylmethoxy (**3b**) groups were introduced into the fifth-position of the indole ring (Figure 3). In addition, compounds in which 4-methoxy (**3c**), 4-chloro (**3d**), and 4-nitro (**3e**) groups were introduced into the benzene ring of **2e** as electron-donating, weakly electron-withdrawing, and strongly electron-withdrawing groups, respectively, were synthesized. 5-Benzyloxy derivative **2e** was subjected to tosylation, followed by elimination of the tosyl group under basic conditions to afford 6′,7′-dehydro compound **5** (Figure 4). Compound **2e** was oxidized by Dess–Martin periodinane to the 7′-oxo derivative **6**, which was further reduced by sodium borohydride to afford alcohol **7**, corresponding to the 7′-epi-form of **2e**. The C-7 stereochemistry of **7** was confirmed by the NOESY correlation between H-5′ and H-7′.

The inhibitory effects of CTLA-4 and PD-L1 on the expression of the aforementioned synthesized compounds were investigated (Table 1). Results for **3a** and **3b** indicate that a bulkier substituent such as a naphthalene ring is desirable. The introduction of substituents into the benzene ring of **2e** slightly improved the activity, and **3e** with a 4-nitro group exhibited particularly good results. On the other hand, **5**–**7**, in which the iridoid moiety of **2e** was modified, did not give good results; particularly, when the oxygen functional group at C-7 was removed, the inhibitory effect was weakened. Therefore, it is crucial to retain the iridoid moiety in this compound.

### Synthesis of Compounds Using Tryptophan Derivatives and Their Immune Checkpoint Inhibitory Activity

**FIGURE 5:**
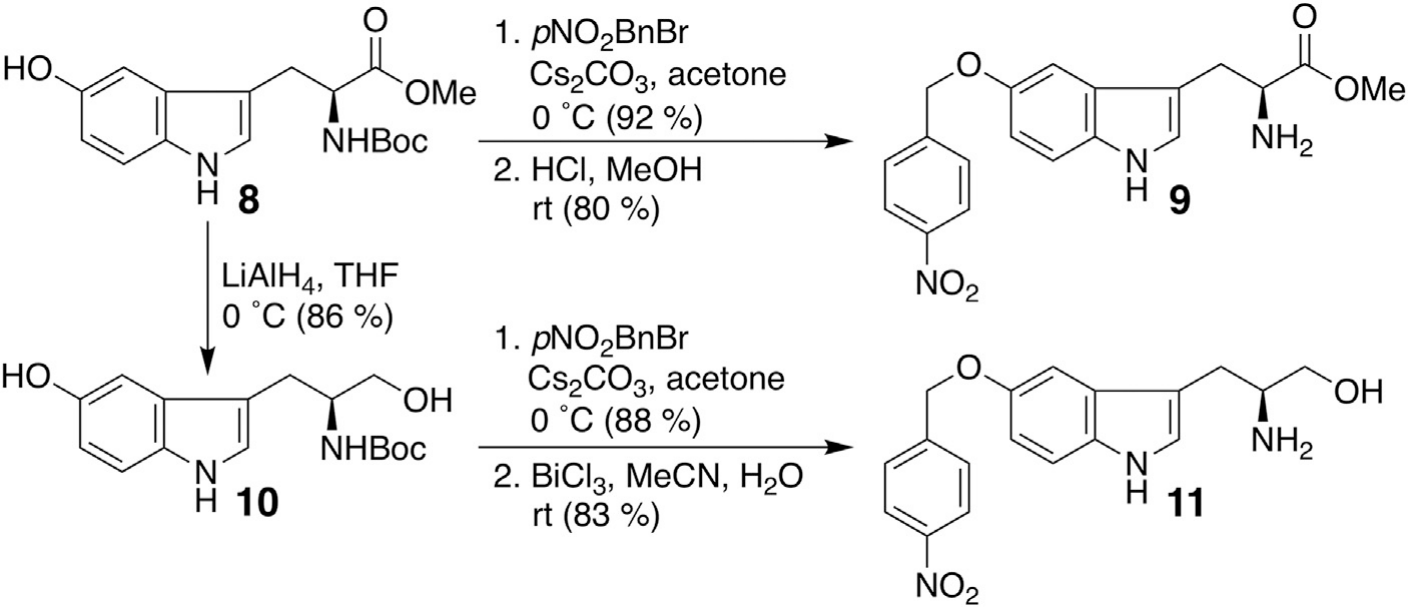
Synthesis of compounds **9** and **11**.

**FIGURE 6:**
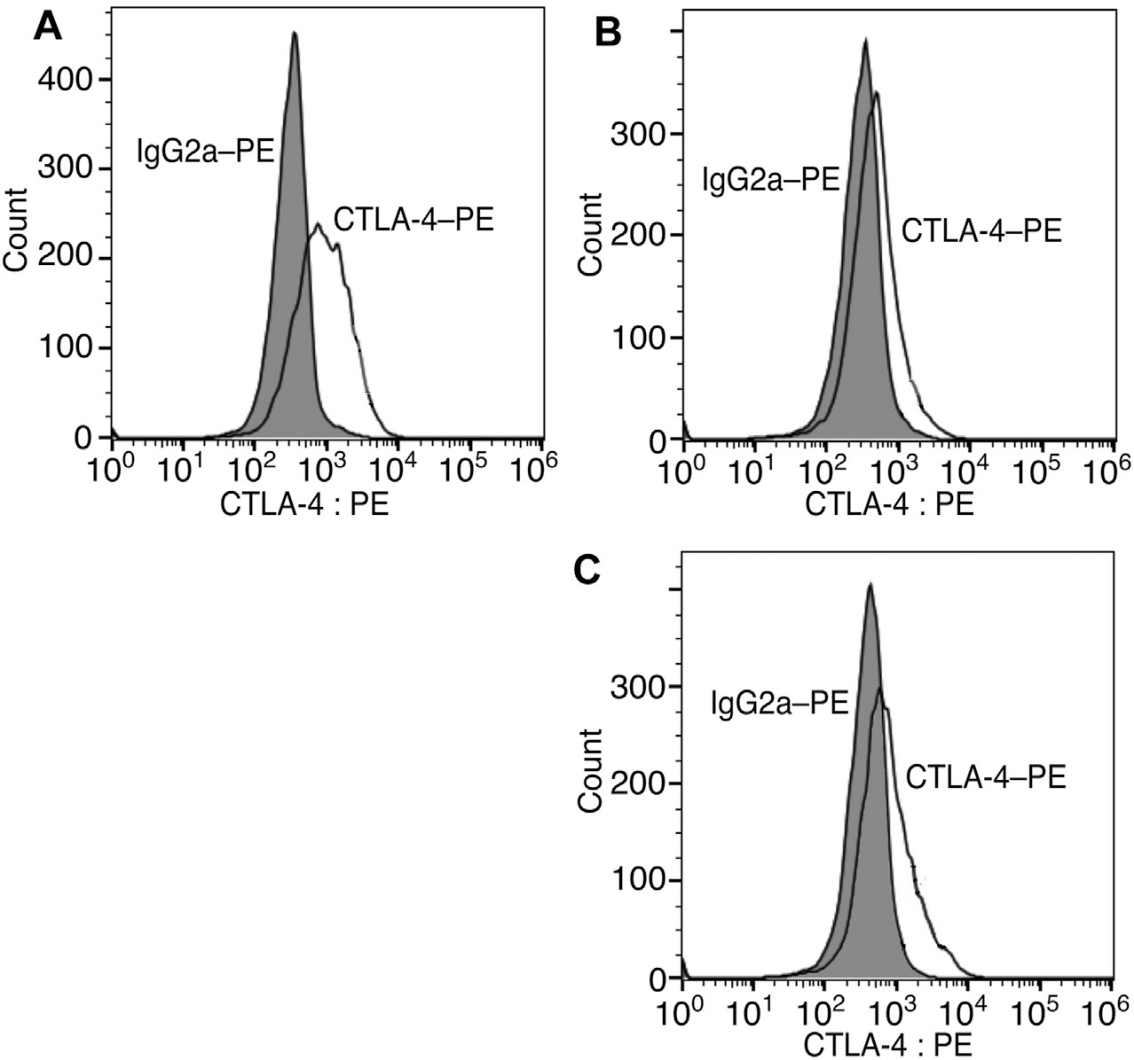
Inhibition of cell surface CTLA-4 protein expression via compounds **12** and **13**. Representative flow cytometry histograms of CTLA-4 on MT-2 cells untreated **(A)** or treated with 20 μM of **12 (B)** or **13 (C)**.

**FIGURE 7:**
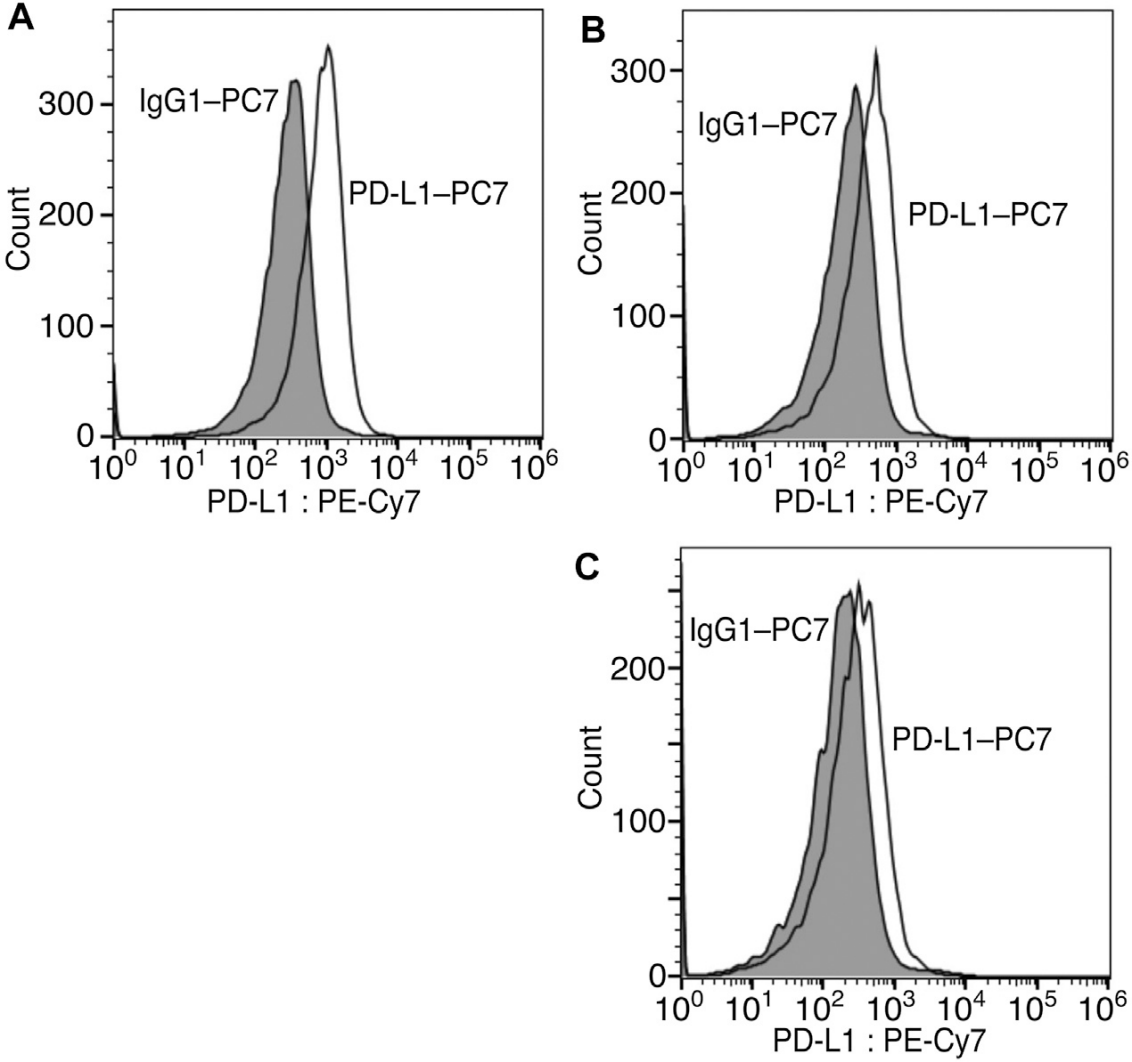
Inhibition of cell surface PD-L1 protein expression via compounds **12** and **13**. Representative flow cytometry histograms of PD-L1 on THP-1 cells untreated **(A)** or treated with 15 μM of **12 (B)** or **13 (C)**.

Finally, we modified the chemical structure of **3e**, which gave the best results thus far, and we synthesized compounds using tryptophan derivatives to further improve their biological activity. We synthesized *N*-Boc-5-hydroxy-L-tryptophan methyl ester (**8**) according to a previously reported method (Zhu et al., 2015), followed by the etherification of the *p*-nitrobenzyl group and removal of the Boc group to afford **9** (Figure 5). In addition, **7** was reduced to amino alcohol derivative **10**, and the etherification of the *p*-nitrobenzyl group and removal of the Boc group were performed to obtain **11**. Mixtures of iridoids, which were α-glucosidase-treated extracts of *C. officinalis*, were then subjected to condensation with **10** and **11** to afford diversity-enhanced extracts; these extracts were separated to afford corresponding compounds **12** and **13**, respectively (Figure 1C). The inhibitory effects of compounds **12** and **13** on the expression of CTLA-4 and PD-L1 were slightly enhanced compared to compound **3e** (Table 1); in particular, the inhibitory effects of **12** and **13** toward PD-L1 expression were enhanced by approximately sevenfold compared with that of **1**. Thus, whether **12** and **13** suppress not only CTLA-4 and PD-L1 gene expression but also protein expression on the cell surface is investigated. The consistent change in the surface CTLA-4 expression was identified by flow cytometry analysis, where the mean fluorescence intensity (MFI) of MT-2 cells treated with **12** and **13** exhibited 73 and 29% decrease, respectively, at a concentration of 20 μM (Figure 6). The consistent change in the surface PD-L1 expression was also identified by flow cytometry analysis, where the MFI of THP-1 cells treated with **12** and **13** exhibited 55 and 76% decrease, respectively, at a concentration of 15 μM (Figure 7).

## Conclusion

Based on the unnatural pentacyclic compound **1** obtained from the diversity-enhanced extracts of Japanese cornelian cherry, **12** and **13** were obtained with immune checkpoint inhibitory activities *via* the introduction of substituents and conversion of functional groups. Although it is difficult to obtain these unnatural pentacyclic indole alkaloid–like compounds and their derivatives by other synthetic methods, they could be efficiently obtained by utilizing the diversity-enhanced extracts. Compounds **12** and **13** suppressed the CTLA-4 and PD-L1 gene expression and their protein expression on the cell surface. Although their potency is not as high as those of the existing small-molecule immune checkpoint inhibitors against CTLA-4 (Huxley et al., 2004; Zeng et al., 2013) or PD-L1 (Skalniak et al., 2017; Taylor et al., 2018; Park et al., 2021), these compounds are the first reported dual small-molecule inhibitors of CTLA-4 and PD-L1. Therefore, using these compounds can provide an option for cancer immunotherapy, either as monotherapy or in combination with monoclonal antibody-based blockers. On the other hand, since these compounds inhibit CTLA-4 and PD-L1 expression at the same time, it is unlikely that they specifically inhibit only the expression of these two proteins. Rather, they may suppress the expression of several related proteins by suppressing the expression of genes upstream of these proteins. These are issues that need to be addressed in the future.

## Experimental Section

### General Methods

Analytical TLC was performed on silica gel 60F_254_ and RP-18F_254_S (Merck). Column chromatography was carried out on silica gel 60 (70–230 mesh, Merck) and COSMOSIL 75C_18_-OPN (Nacalai Tesque, Inc.). NMR spectra were recorded on JEOL ECA-600 and AL-400. Chemical shifts for ^1^H and ^13^C NMR are given in parts per million (δ) relative to tetramethylsilane (δH 0.00) and residual solvent signals (δC 77.0) as internal standards. Mass spectra were measured on JEOL JMS-700, JMS-DX303, and JMS-T 100 GC. Optical rotations were measured on JASCO P-1030.

### Preparation of Mixtures of Iridoids From *Cornus officinalis*

Accessory fruits (500 g) of *Cornus officinalis*, which was purchased from Uchidawakanyaku Ltd. (Tokyo, Japan), were extracted twice with methanol (3 L) at room temperature to give the extract (105 g). This extract was partitioned with ethyl acetate and water to yield water solubles. The water solubles was subjected to activated charcoal (200 g) and then successively eluted with water (2 L), 5% ethanol-water solution (2 L) and methanol (2 L). The methanol eluent was concentrated *in vacuo* to give glycoside-rich fractions (16.1 g). The glycoside-rich fractions were dissolved in 0.05 M citrate buffer (pH 6.0) (600 ml), and β-glucosidase (from Sweet Almond, Toyobo Co., Ltd.) (300 mg) was added to the solution. After being stirred for 2 days at 45°C, the reaction mixture was extracted with ethyl acetate three times. The combined organic layer was washed with water, dried over sodium sulfate, and concentrated *in vacuo* to give a mixture of iridoids (1.03 g).

### Preparation of Compounds 2a–2h by Using the Diversity-Enhanced Extracts

The mixture of iridoids (140 mg) was dissolved in dichloromethane (6 ml), and 5-bromotryptamine (133 mg, 0.559 mmol) and bismuth (III) trifluoromethanesulfonate (37 mg, 0.056 mmol) were added to the solution. After being stirred for 12 h at room temperature, the reaction mixture was poured into saturated sodium bicarbonate solution and extracted with ethyl acetate three times. The combined organic layer was washed with water and brine, dried over sodium sulfate, and concentrated *in vacuo* to give diversity-enhanced extracts (179 mg). They were chromatographed over silica gel and the column eluted with chloroform-methanol mixtures with increasing polarity to afford chloroform-methanol (19:1) eluent (45 mg), which was separated by ODS column using water-acetonitrile solvent system to give water-acetonitrile (3:7) eluent (21 mg). It was subjected to recycle preparative HPLC (column, YMC-GPC T-2000 (ϕ 20 mm × 600 mm, TMC Co., Ltd.); solvent, ethyl acetate) to give compound **2a** (3.8 mg, 2.7% (w/w) from the mixture of iridoids). Analytical data for **2a**: (α)_D_^23^–65.2° (*c* 0.181, CHCl_3_); ^1^H NMR (600 MHz, CDCl_3_) δ 8.05 (1H, br. s), 7.55 (1H, d, *J* = 1.7 Hz), 7.30 (1H, s), 7.22 (1H, dd, *J* = 8.6, 1.7 Hz), 7.17 (1H, d, *J* = 8.6 Hz), 4.34–4.37 (1H, br. m), 4.21 (1H, t, *J* = 4.6 Hz), 3.71 (1H, dd, *J* = 15.3, 5.8 Hz), 3.60 (3H, s), 3.42 (1H, dt, *J* = 15.3, 5.6 Hz), 2.95 (1H, dd, *J* = 15.1, 5.6 Hz), 2.84–2.89 (1H, m), 2.69 (1H, dd, *J* = 15.5, 4.7 Hz), 2.36 (1H, dt, *J* = 12.8, 4.9 Hz), 2.21 (1H, dd, *J* = 14.8, 8.1 Hz), 2.02–2.06 (1H, m) 1.79 (1H, dt, *J* = 14.8, 5.7 Hz), 1.17 (3H, d, *J* = 7.0 Hz); ^13^C NMR (150 MHz, CDCl_3_) δ 168.4, 144.3, 134.8, 134.4, 129.0, 124.7, 120.7, 113.0, 112.4, 108.5, 104.9, 74.5, 52.7, 51.3, 50.7, 44.8, 42.5, 41.4, 30.9, 22.0, 12.7; LREIMS: *m/z* 432 (M+2)^+^, 430 (M)^+^ (100%), 359, 313, 248, 149, 57 (base); HREIMS: *m/z* 430.0893 (M)^+^ (430.0891 calcd. for C_21_H_23_^79^BrN_2_O_3_).

By the use of the procedure described above, compounds **2b** (3.1 mg, 2.6% (w/w) from the mixture of iridoids), **2c** (16 mg, 12%), **2d** (9.5 mg, 28%), **2e** (13 mg, 16%), **2f** (4.3 mg, 3.0%), **2g** (12 mg, 8.4%), **2h** (15 mg, 34%), and **2i** (16 mg, 16%) were synthesized from the mixture of iridoids and the corresponding substituted tryptamine, respectively.

Analytical data for **2b**: yellowish oil; (α)_D_^24^–188° (*c* 0.199, CHCl_3_); ^1^H NMR (600 MHz, CDCl_3_) δ 8.09 (1H, br. s), 7.38 (1H, d, *J* = 1.9 Hz), 7.31 (1H, s) 7.20 (1H, d, *J* = 8.5 Hz), 7.08 (1H, dd, *J* = 8.5, 1.9 Hz), 4.35–4.39 (1H, br. m), 4.22 (1H, dd, *J* = 5.0, 4.9 Hz), 3.71 (1H, dd, *J* = 15.3, 5.8 Hz), 3.60 (3H, s), 3.42 (1H, dt, *J* = 15.3, 5.6 Hz), 2.95 (1H, dd, *J* = 15.1, 5.6 Hz), 2.84–2.90 (1H, m), 2.69 (1H, dd, *J* = 15.5, 4.6 Hz), 2.36 (1H, ddd, *J* = 10.8, 7.0, 2.7 Hz), 2.21 (1H, ddd, *J* = 14.7, 8.1, 1.3 Hz), 2.03–2.06 (1H, m), 1.79 (1H, dt, *J* = 14.8, 5.7 Hz), 1.17 (3H, d, *J* = 6.9 Hz); ^13^C NMR (150 MHz, CDCl_3_) δ 168.4, 144.3, 134.9, 134.1, 128.3, 125.5, 122.2, 117.6, 111.9, 108.5, 104.9, 74.5, 52.7, 51.3, 50.7, 44.8, 42.5, 41.4, 30.8, 22.0, 12.7; LREIMS: *m/z* 388 (M+2)^+^, 386 (M)^+^, 313, 204, 57 (100%); HREIMS: *m/z* 386.1369 (M)^+^ (386.1397 calcd. for C_21_H_23_^35^ClN_2_O_3_).

Analytical data for **2c**: yellowish oil; (α)_D_^25^–113° (*c* 0.760, CHCl_3_); ^1^H NMR (600 MHz, CDCl_3_) δ 8.18 (1H, br. s), 7.33 (1H, s), 7.22 (1H, d, *J* = 1.0 Hz), 7.19 (1H, d, *J* = 8.3 Hz), 6.95 (1H, dd, *J* = 8.3, 1.0 Hz), 4.33–4.37 (1H, br. m), 4.21 (1H, t, *J* = 4.7 Hz), 3.69 (1H, dd, *J* = 15.2, 6.0 Hz), 3.60 (3H, s), 3.43 (1H, dt, *J* = 15.2, 5.5 Hz), 2.94 (1H, dd, *J* = 14.9, 5.5 Hz), 2.85–2.91 (1H, m), 2.70 (1H, dd, *J* = 15.3, 4.7 Hz), 2.41 (3H, s), 2.35 (1H, dt, *J* = 11.8, 4.8 Hz), 2.19 (1H, ddd, *J* = 14.8, 8.1, 1.2 Hz) 2.01–2.07 (1H, m), 1.76 (1H, dt, *J* = 14.7, 5.9 Hz), 1.16 (3H, d, *J* = 7.0 Hz); ^13^C NMR (150 MHz, CDCl_3_) δ 168.6, 144.7, 134.1, 133.5, 129.0, 127.3, 123.4, 117.7, 110.7, 108.1, 104.2, 74.4, 52.9, 51.5, 50.6, 44.9, 42.5, 41.5, 31.0, 22.2, 21.4, 12.8; LREIMS: *m/z* 366 (M)^+^ (100%), 307, 295, 184; HREIMS: *m/z* 366.1956 (M)^+^ (366.1942 calcd. for C_22_H_26_N_2_O_3_).

Analytical data for **2d**: yellowish oil; [α]_D_^25^–153° (*c* 0.631, CHCl_3_); ^1^H NMR (600 MHz, CDCl_3_) δ 8.20 (1H, br. s), 7.32 (1H, s), 7.19 (1H, d, *J* = 8.8 Hz), 6.87 (1H, d, *J* = 2.4 Hz), 6.79 (1H, dd, *J* = 8.4, 2.4 Hz), 4.34–4.37 (1H, br. m), 4.20 (1H, t, *J* = 4.7 Hz), 3.82 (3H, s), 3.70 (1H, dd, *J* = 14.8, 5.7 Hz), 3.59 (3H, s), 3.43 (1H, dt, *J* = 14.8, 5.4 Hz), 2.94 (1H, dd, *J* = 15.1, 5.4 Hz), 2.85–2.91 (1H, m), 2.70 (1H, dd, *J* = 15.1, 4.7 Hz), 2.34–2.36 (1H, m), 2.20 (1H, ddd, *J* = 14.7, 8.0, 1.2 Hz), 2.00–2.06 (1H, m), 1.76 (1H, dt, *J* = 14.7, 5.8 Hz), 1.19 (3H, d, *J* = 7.0 Hz); ^13^C NMR (150 MHz, CDCl_3_) δ 168.6, 154.2, 144.6, 134.3, 130.9, 127.5, 111.74, 111.73, 108.4, 104.4, 100.2, 74.5, 56.0, 52.9, 51.5, 50.6, 44.8, 42.5, 41.4, 30.9, 22.2, 12.8; LREIMS: *m/z* 382 (M)^+^, 364, 323, 129, 57 (100%); HREIMS: *m/z* 382.1896 (M)^+^ (382.1891 calcd. for C_22_H_26_N_2_O_4_).

Analytical data for **2e**: yellowish oil; (α)_D_^26^–164° (*c* 0.751, CHCl_3_); ^1^H NMR (600 MHz, CDCl_3_) δ 8.20 (1H, br. s), 7.45 (2H, t, *J* = 8.3 Hz), 7.35 (2H, t, *J* = 8.3 Hz), 7.32 (1H, s), 7.29 (1H, t, *J* = 8.3 Hz), 7.20 (1H, d, *J* = 8.7 Hz), 6.97 (1H, d, *J* = 2.5 Hz), 6.87 (1H, dd, *J* = 8.7, 2.5 Hz), 5.07 (2H, s), 4.33–4.36 (1H, br. m), 4.20 (1H, t, *J* = 5.0 Hz), 3.70 (1H, dd, *J* = 15.5, 6.0 Hz), 3.60 (3H, s), 3.46 (1H, dt, *J* = 15.5, 5.1 Hz), 2.94 (1H, dd, *J* = 15.4, 5.1 Hz), 2.84–2.89 (1H, m), 2.68 (1H, dd, *J* = 15.5, 4.4 Hz), 2.34–2.38 (1H, m), 2.19 (1H, ddd, *J* = 14.7, 8.0, 1.2 Hz), 2.01–2.06 (1H, m), 1.77 (1H, dt, *J* = 14.7, 5.8 Hz), 1.15 (3H, d, *J* = 7.0 Hz); ^13^C NMR (150 MHz, CDCl_3_) δ 168.5, 153.4, 144.6 137.6, 134.3, 131.1, 128.4 (2C), 127.7, 127.5 (2C), 112.4, 111.7, 108.4, 104.3, 101.9, 74.5, 71.0, 52.9, 51.5, 44.8, 42.5, 41.4, 30.8, 22.2, 12.8; LREIMS: *m/z* 458 (M)^+^ (100%), 440, 399, 367, 185, 91; HREIMS: *m/z* 458.2194 (M)^+^ (458.2206 calcd. for C_28_H_30_N_2_O_4_).

Analytical data for **2f**: yellowish oil; (α)_D_^25^–44.8° (*c* 0.313, CHCl_3_); ^1^H NMR (600 MHz, CDCl_3_) δ 8.19 (1H, br. s), 7.32 (1H, s), 7.31 (1H, d, *J* = 8.3 Hz), 7.28 (1H, d, *J* = 1.6 Hz), 7.04 (1H, dd, *J* = 8.3, 1.6 Hz), 4.35–4.39 (1H, br. m), 4.22 (1H, t, *J* = 5.0 Hz), 3.71 (1H, dd, *J* = 15.5, 5.5 Hz), 3.61 (3H, s), 3.43 (1H, dt, *J* = 15.5, 5.5 Hz), 2.95 (1H, dd, *J* = 15.0, 5.5 Hz), 2.93–2.85 (1H, m), 2.72 (1H, dd, *J* = 15.2, 5.2 Hz), 2.37 (1H, ddd, *J* = 10.8, 3.3, 1.6 Hz), 2.22 (1H, ddd, *J* = 14.8, 8.0, 1.1 Hz), 2.07–2.03 (1H, m), 1.78 (1H, dt, *J* = 14.8, 5.7 Hz), 1.17 (3H, d, *J* = 7.1 Hz); ^13^C NMR (150 MHz, CDCl_3_) δ 168.5, 144.5, 136.1, 134.1, 127.7, 125.8, 120.4, 118.8, 111.0, 108.8, 104.7, 74.5, 52.7, 51.3, 50.7, 44.8, 42.5, 41.4, 30.9, 22.0, 12.7; LREIMS: *m/z* 388 (M+2)^+^, 386 (M)^+^ (base), 313, 204; HREIMS: *m/z* 386.1431 (M)^+^ (386.1397 calcd. for C_21_H_23_^35^ClN_2_O_3_).

Analytical data for **2g**: yellowish oil; (α)_D_^25^–69.2° (*c* 0.460, CHCl_3_); ^1^H NMR (600 MHz, CDCl_3_) δ 8.50 (1H, br. s), 7.34 (1H, s), 7.31 (1H, dd, *J* = 8.6, 5.1 Hz), 6.98 (1H, dd, *J* = 7.6, 2.1 Hz), 6.80–6.85 (1H, m), 4.33–4.36 (1H, br. m), 4.20 (1H, t, *J* = 4.9 Hz), 3.70 (1H, dd, *J* = 13.4, 5.9 Hz), 3.59 (3H, s), 3.42 (1H, dt, *J* = 12.5, 4.5 Hz), 2.93 (1H, dd, *J* = 14.4, 7.7 Hz), 2.84–2.90 (1H, m), 2.71 (1H, dd, *J* = 15.3, 4.2 Hz) 2.40 (1H, dt, *J* = 13.0, 4.6 Hz), 2.20 (1H, dd, *J* = 14.7, 8.0 Hz) 1.77 (1H, dt, *J* = 14.7, 5.7 Hz) 1.15 (3H, d, *J* = 6.8 Hz); ^13^C NMR (150 MHz, CDCl_3_) δ 168.6, 159.7 (d, *J* = 237 Hz), 144.6, 135.7 (d, *J* = 12.2 Hz), 133.6 (d, *J* = 2.9 Hz), 123.7, 118.5 (d, *J* = 10.0 Hz), 108.4, 108.1 (d, *J* = 24.4 Hz), 104.5, 97.5 (d, *J* = 25.8 Hz), 74.5, 52.7, 51.4, 50.7, 44.7, 42.4, 41.4, 30.8, 22.8, 12.7; LREIMS: *m/z* 370 (M)^+^ (base), 311, 188, 57; HREIMS: *m/z* 370.1685 (M)^+^ (370.1693 calcd. for C_21_H_23_FN_2_O_3_).

Analytical data for **2h**: yellowish oil; (α)_D_^26^–24.2° (*c* 1.00, CHCl_3_); ^1^H NMR (600 MHz, CDCl_3_) δ 8.60 (1H, br. s), 7.34 (1H, s), 7.29 (1H, d, *J* = 8.5 Hz), 6.81 (1H, d, *J* = 2.1 Hz), 6.75 (1H, dd, *J* = 8.5, 2.1 Hz), 4.30–4.34 (1H, br. m), 4.20 (1H, t, *J* = 4.8 Hz), 3.78 (3H, s), 3.70 (1H, dd, *J* = 15.0, 5.5 Hz), 3.60 (3H, s), 3.40 (1H, dt, *J* = 15.0, 5.4 Hz), 2.94 (1H, dd, *J* = 14.6, 5.4 Hz), 2.83–2.86 (1H, m), 2.70 (1H, dd, *J* = 15.3, 4.2 Hz), 2.39 (1H, dt, *J* = 13.0, 4.5 Hz), 2.23 (1H, ddd, *J* = 14.7, 8.0, 0.8 Hz) 1.99–2.02 (1H, m), 1.77 (1H, dt, *J* = 14.7, 7.1 Hz), 1.19 (3H, d, *J* = 7.1 Hz); ^13^C NMR (150 MHz, CDCl_3_) δ 168.7, 156.2, 144.9, 136.6, 132.2, 121.5, 118.4, 109.0, 108.1, 104.0, 95.2, 74.3, 55.7, 52.8, 51.5, 50.6, 44.7, 42.3, 41.4, 30.8, 22.2, 12.8; LREIMS: *m/z* 382 [M]^+^ (base), 323, 200, 120; HREIMS: *m/z* 382.1851 [M]^+^ (382.1891 calcd. for C_22_H_26_N_2_O_4_).

Analytical data for **2i**: yellowish oil; (α)_D_^24^–24.9° (*c* 0.692, CHCl_3_); ^1^H NMR (600 MHz, CDCl_3_) δ 8.10 (1H, br. s), 7.34 (1H, s), 7.29 (1H, d, *J* = 7.8 Hz), 7.00 (1H, dd, *J* = 7.8, 7.3 Hz), 6.94 (1H, d, *J* = 7.3 Hz), 4.33–4.37 (1H, br. m), 4.22 (1H, t, *J* = 4.8 Hz), 3.70 (1H, dd, *J* = 15.1, 5.7 Hz), 3.60 (3H, s), 3.44 (1H, dt, *J* = 15.1, 5.4 Hz), 2.94 (1H, dd, *J* = 14.8, 5.4 Hz), 2.88–2.94 (1H, m), 2.70 (1H, dd, *J* = 15.5, 4.6 Hz), 2.47 (3H, s), 2.42 (1H, dt, *J* = 12.7, 4.1 Hz), 2.23 (1H, ddd, *J* = 14.7, 8.0, 1.0 Hz), 2.02–2.09 (1H, m), 1.78 (1H, dt, *J* = 14.7, 5.8 Hz), 1.19 (3H, d, *J* = 6.9 Hz); ^13^C NMR (150 MHz, CDCl_3_) δ 168.6, 144.7, 135.3, 133.0, 126.6, 122.6, 120.4, 119.9, 115.6, 109.1, 104.3, 74.5, 52.9, 51.5, 50.6, 44.7, 42.5, 41.6, 31.0, 22.3, 16.8, 12.7; LREIMS: *m/z* 366 (M)^+^ (base), 307, 293, 184, 57; HREIMS: *m/z* 366.1968 (M)^+^ (366.1942 calcd. for C_22_H_26_N_2_O_3_).

### Synthesis of Compound 2j

A mixture of compound **2e** (29 mg, 0.064 mmol) and palladium hydroxide (4.0 mg) (20% on carbon, wet with 50% water content) in methanol (3 ml) was stirred at room temperature under hydrogen atmosphere for 5 h. After filtration through a Celite pad, the filtrate was concentrated *in vacuo*. The residue was chromatographed over ODS eluted by water-acetonitrile (3:7) to afford **2j** (21 mg, 94%). Analytical data for **2j**: yellowish powder; (α)_D_^26^–118° (*c* 0.190, methanol); ^1^H NMR (600 MHz, methanol-*d*_4_) δ 7.45 (1H, s), 7.14 (1H, d, *J* = 8.6 Hz), 6.75 (1H, d, *J* = 2.2 Hz), 6.62 (1H, dd, *J* = 8.6, 2.2 Hz), 4.40–4.43 (1H, br. m), 4.15 (1H, dt, *J* = 5.2, 2.2 Hz), 3.77 (1H, dd, *J* = 15.0, 5.6 Hz), 3.60 (3H, s), 3.40 (1H, dt, *J* = 15.0, 5.4 Hz), 2.94 (1H, dd, *J* = 15.0, 5.4 Hz), 2.78–2.84 (1H, m), 2.66 (1H, dd, *J* = 15.0, 4.1 Hz), 2.36 (1H, ddd, *J* = 10.7, 6.6, 2.3 Hz), 2.14 (1H, ddd, *J* = 14.2, 7.7, 2.3 Hz), 2.01–2.10 (1H, m), 1.77 (1H, dt, *J* = 14.2, 5.9 Hz), 1.19 (3H, d, *J* = 7.0 Hz); ^13^C NMR (150 MHz, methanol-*d*_4_) δ 171.0, 151.3, 147.1, 135.8, 132.8, 129.1, 112.5, 111.9, 108.1, 104.4, 102.9, 74.7, 54.6, 52.7, 51.1, 46.7, 43.1, 42.4, 32.3, 23.5, 13.4; LREIMS: *m/z* 368 (M)^+^ (base), 309, 297, 271, 186; HREIMS: *m/z* 368.1753 (M)^+^ (368.1735 calcd. for C_21_H_24_N_2_O_4_).

### Synthesis of Compound 3a

Cesium carbonate (26 mg, 0.081 mmol), potassium iodide (10 mg, 0.063 mmol), and cyclohexylmethyl bromide (15 μL, 0.11 mmol) were added to a solution of compound **2j** (8.6 mg, 0.026 mmol) in DMF (1 ml) at room temperature. After being stirred for 24 h at 70°C, the reaction mixture was cooled to room temperature, poured into saturated ammonium chloride solution, and extracted with ethyl acetate three times. The combined organic layer was washed with water and brine, dried over sodium sulfate, and concentrated *in vacuo*. The residue was chromatographed over silica gel eluted by hexane-ethyl acetate (3:2) to afford **3a** (3.5 mg, 30%). Analytical data for **3a**: yellowish oil; (α)_D_^29^–186° (*c* 0.161, CHCl_3_); ^1^H NMR (600 MHz, CDCl_3_) δ 7.95 (1H, br. s), 7.33 (1H, s), 7.18 (1H, d, *J* = 8.8 Hz), 6.86 (1H, d, *J* = 2.3 Hz), 6.86 (1H, dd, *J* = 8.8, 2.3 Hz), 4.30–4.34 (1H, br. m), 4.20 (1H, t, *J* = 5.1 Hz), 3.76 (2H, d, *J* = 6.6 Hz), 3.70–3.72 (1H, m), 3.60 (3H, s), 3.43 (1H, dt, *J* = 14.8, 5.7 Hz), 2.95 (1H, dd, *J* = 15.2, 5.7 Hz), 2.84–2.90 (1H, m), 2.69 (1H, dd, *J* = 15.2, 4.7 Hz), 2.33 (1H, dt, *J* = 8.8, 4.0 Hz), 2.19 (1H, ddd, *J* = 14.7, 8.0, 1.3 Hz), 2.02–2.07 (1H, m), 1.62–1.88 (6H, m), 1.20–1.32 (3H, m), 1.16 (3H, d, *J* = 6.8 Hz), 0.98–1.10 (3H, m); ^13^C NMR (150 MHz, CDCl_3_) δ 168.5, 153.9, 144.6, 134.1, 130.8, 127.5, 112.4, 111.5, 108.5, 104.2, 101.4, 74.5, 74.4, 52.9, 51.5, 50.6, 44.9, 42.5, 41.5, 37.9, 30.9, 30.0 (2C), 26.6, 25.8 (2C), 22.2, 12.8; LREIMS: *m/z* 464 (M)^+^ (base), 405, 367, 282; HREIMS: *m/z* 464.2628 (M)^+^ (464.2673 calcd. for C_28_H_36_N_2_O_4_).

### Synthesis of Compounds 3b–3e

Cesium carbonate (26 mg, 0.081 mmol) and 2-(bromomethyl)naphthalene (8.3 mg, 0.038 mmol) were added to a solution of compound **2j** (10 mg, 0.027 mmol) in DMF (1 ml) at room temperature. After being stirred for 18 h at room temperature, the reaction mixture was cooled to room temperature, poured into saturated ammonium chloride solution, and extracted with ethyl acetate three times. The combined organic layer was washed with water and brine, dried over sodium sulfate, and concentrated *in vacuo*. The residue was chromatographed over silica gel eluted by hexane-ethyl acetate (2:3) to afford **3b** (5.3 mg, 40%). Analytical data for **3b**: yellow oil; (α)_D_^27^–165° (*c* 0.357, CHCl_3_); ^1^H NMR (400 MHz, CDCl_3_) δ 7.82–7.91 (5H, m), 7.57 (1H, d, *J* = 9.0 Hz), 7.46–7.48 (2H, m), 7.35 (1H, s), 7.21 (1H, d, *J* = 8.8 Hz), 7.03 (1H, d, *J* = 2.4 Hz), 6.92 (1H, d, *J* = 8.8 Hz), 5.05 (2H, s), 4.34–4.37 (1H, br. m), 4.23 (1H, t, *J* = 4.6 Hz), 3.71 (1H, dd, *J* = 14.3, 5.6 Hz), 3.61 (3H, s), 3.41–3.47 (1H, m), 2.98 (1H, ddd, *J* = 7.2, 7.2, 7.2 Hz), 2.84–2.93 (1H, m), 2.68 (1H, dd, *J* = 15.6, 4.6 Hz), 2.31–2.37 (1H, m), 2.20 (1H, dd, *J* = 15.6, 7.9 Hz) 2.04–2.11 (1H, m), 1.76–1.84 (1H, m), 1.19 (3H, d, *J* = 7.3 Hz) ^13^C NMR (100 MHz, CDCl_3_) δ 168.5, 153.5, 144.6, 135.2, 134.3, 133.3, 133.0, 131.2, 128.2, 128.0, 127.9, 127.7, 126.2, 126.1, 125.2, 125.1, 112.6, 111.7, 108.6, 104.3, 102.1, 74.4, 71.2, 52.9, 51.5, 50.6, 45.0, 42.5, 41.5, 31.0, 22.2, 12.8; EIMS: *m/z* 508 (M)^+^, 367, 141 (base); HREIMS: *m/z* 508.2368 (M)^+^ (508.2360 calcd. for C_32_H_32_N_2_O_4_).

By the use of the procedure described above, compounds **3c** (3.8 mg, 36%), **3d** (6.0 mg, 44%), and **3e** (5.9 mg, 37%) were synthesized from compound **2j** and the corresponding substituted benzyl bromide, respectively.

Analytical data for **3c**: yellow oil; (α)_D_^28^–167° (*c* 0.190, CHCl_3_); ^1^H NMR (400 MHz, CDCl_3_) δ 7.85 (1H, s), 7.35–7.39 (3H, m), 7.21 (1H, d, *J* = 8.5 Hz), 6.97 (1H, d, *J* = 2.6 Hz), 6.87–6.92 (3H, m), 5.01 (2H, s), 4.35–4.38 (1H, br. m), 4.23 (1H, br. s), 3.82 (3H, s), 3.72 (1H, dd, *J* = 15.4, 5.8 Hz), 3.62 (3H, s), 3.42–3.49 (1H, m), 2.99 (1H, ddd, *J* = 7.2, 7.2, 7.2 Hz), 2.85–2.94 (1H, m), 2.68–2.74 (1H, m), 2.30–2.35 (1H, m), 2.22 (1H, dd, *J* = 14.5, 7.8 Hz), 2.04–2.11 (1H, m), 1.77–1.84 (1H, m), 1.19 (3H, d, *J* = 7.2 Hz); ^13^C NMR (100 MHz, CDCl_3_) δ 168.5, 159.4, 153.6, 144.6, 134.2, 131.1, 129.8, 129.2 (2C), 127.6, 113.9 (2C), 112.7, 111.6, 108.7, 104.3, 102.0, 74.4, 70.8, 56.3, 52.9, 51.5, 50.7, 45.0, 42.6, 41.6, 31.0, 22.2, 12.9; EIMS: *m/z* 488 (M)^+^, 367, 121 (base); HREIMS: *m/z* 488.2302 (M)^+^ (488.2309 calcd. for C_29_H_32_N_2_O_5_).

Analytical data for **3d**: yellow oil; (α)_D_^27^–157° (*c* 0.306, CHCl_3_); ^1^H NMR (400 MHz, CDCl_3_) δ 7.89 (1H, s), 7.39 (2H, d, *J* = 8.3 Hz), 7.34 (2H, d, *J* = 8.3 Hz), 7.33 (1H, s), 7.21 (1H, d, *J* = 8.8 Hz), 6.95 (1H, d, *J* = 2.3 Hz), 6.87 (1H, dd, *J* = 8.8, 2.3 Hz), 5.05 (2H, s), 4.35–4.37 (1H, br. m), 4.23 (1H, t, *J* = 4.5 Hz), 3.72 (1H, dd, *J* = 15.2, 5.6 Hz), 3.62 (3H, s), 3.41–3.49 (1H, m), 2.98 (1H, dd, *J* = 14.4, 7.5 Hz), 2.85–2.92 (1H, m), 2.69 (1H, dd, *J* = 15.5, 4.5 Hz), 2.31–2.36 (1H, m), 2.19–2.25 (1H, m), 2.04–2.12 (1H, m), 1.77–1.84 (1H, m), 1.19 (3H, d, *J* = 7.1 Hz); ^13^C NMR (100 MHz, CDCl_3_) δ 168.5, 153.2, 144.6, 136.2, 134.3, 133.5, 131.2, 128.8 (2C), 128.6 (2C), 127.6, 112.4, 111.7, 108.6, 104.4, 102.1, 74.4, 70.2, 52.9, 51.5, 50.7, 45.0, 42.6, 41.5, 31.0, 22.2, 12.8; EIMS: *m/z* 494 (M+2)^+^, 492 (M)^+^, 435, 433, 367 (base); HREIMS: *m/z* 492.1819 (M)^+^ (492.1814 calcd. for C_28_H_29_^35^ClN_2_O_4_).

Analytical data for **3e**: yellowish oil; (α)_D_^27^–160° (*c* 0.568, CHCl_3_); ^1^H NMR (400 MHz, CDCl_3_) δ 8.23 (2H, d, *J* = 8.8 Hz), 8.02 (1H, s), 7.64 (2H, d, *J* = 8.8 Hz), 7.34 (1H, s), 7.23 (1H, d, *J* = 8.7 Hz), 6.87 (1H, d, *J* = 2.3 Hz), 6.94 (1H, dd, *J* = 8.7, 2.3 Hz), 5.20 (2H, s), 4.36–4.39 (1H, br. m), 4.23 (1H, t, *J* = 4.5 Hz), 3.72 (1H, dd, *J* = 14.3, 5.6 Hz), 3.62 (3H, s), 3.42–3.48 (1H, m), 2.98 (1H, ddd, *J* = 7.0, 7.0, 7.0 Hz), 2.84–2.93 (1H, m), 2.69 (1H, dd, *J* = 15.3, 4.5 Hz), 2.33–2.39 (1H, m), 2.19–2.24 (1H, m), 2.05–2.10 (1H, m), 1.80 (1H, dt, *J* = 14.6, 5.6 Hz), 1.19 (3H, d, *J* = 7.2 Hz); ^13^C NMR (100 MHz, CDCl_3_) δ 168.5, 152.7, 145.2, 144.5, 134.7, 134.6, 131.3, 127.6 (2C), 123.8, 123.7 (2C), 112.2, 111.8, 108.5, 104.6, 102.1, 74.4, 69.7, 52.8, 51.4, 50.7, 44.8, 42.5, 41.4, 30.9, 22.2, 12.7; EIMS: *m/z* 503 (M)^+^ (base), 444, 367; HREIMS: *m/z* 503.2047 (M)^+^ (503.2055 calcd. for C_28_H_29_N_3_O_6_).

### Synthesis of Compound 4

Triethylamine (30 μL, 0.215 mmol), trimethylamine hydrochloride (6.9 mg, 0.072 mmol), and *p*-toluenesulfonyl chloride (25.8 mg, 0.135 mmol) were added to a solution of compound **2e** (32 mg, 0.070 mmol) in dichloromethane (1.0 ml) at 0°C. After being stirred for 2 h, the reaction mixture was poured into water and extracted with ethyl acetate three times. The combined organic layer was washed with water and brine, dried over sodium sulfate, and concentrated *in vacuo*. The residue was chromatographed over silica gel eluted by hexane-ethyl acetate (2:1) to afford **4** (12 mg, 28%). Analytical data for **4**: yellowish oil; ^1^H NMR (400 MHz, CDCl_3_) δ 7.95 (1H, br. s), 7.81 (2H, d, *J* = 8.4 Hz), 7.20–7.49 (9H, m), 6.96 (1H, d, *J* = 2.3 Hz), 6.90 (1H, dd, *J* = 8.7, 2.3 Hz), 5.08 (2H, s), 4.92 (1H, d, *J* = 5.0 Hz), 4.38–4.41 (1H, m), 3.61–3.74 (2H, m), 3.56 (3H, s), 3.40 (1H, dt, *J* = 6.3, 1.2 Hz), 2.82–2.90 (2H, m), 2.68 (1H, dd, *J* = 15.3, 4.7 Hz), 2.43 (3H, s), 2.37–2.41 (1H, m), 2.26 (1H, dd, *J* = 15.4, 8.6 Hz), 2.10–2.16 (1H, m), 1.78 (1H, dt, *J* = 10.6, 5.2 Hz), 1.11 (3H, d, *J* = 6.9 Hz); ^13^C NMR (100 MHz, CDCl_3_) δ 168.1, 153.5, 144.7, 144.5, 137.6, 134.3, 133.6, 131.1, 129.8 (2C), 126.5 (2C), 127.8 (2C), 127.5 (2C), 112.6, 111.8, 108.5, 104.4, 102.0, 86.8, 71.1, 60.3, 52.2, 51.4, 50.6, 44.9, 40.6, 39.6, 30.3, 22.2, 21.6, 14.2, 12.6; HRFABMS: *m/z* 613.2328 (M + H)^+^ (613.2370 calcd. for C_35_H_37_N_2_O_6_S).

### Synthesis of Compound 5

Sodium acetate (7.2 mg, 0.088 mmol) was added to a solution of **4** (5.6 mg, 0.009 mmol) at room temperature. After being stirred for 8 h at 70°C, the reaction mixture was cooled to room temperature, poured into water, and extracted with ethyl acetate three times. The combined organic layer was washed with water and brine, dried over sodium sulfate, and concentrated *in vacuo*. The residue was chromatographed over silica gel eluted by hexane-ethyl acetate (3:1) to afford **5** (1.9 mg, 45%). Analytical data for **5**: yellowish oil; (α)_D_^26^–59° (*c* 0.095, CHCl_3_); ^1^H NMR (600 MHz, CDCl_3_) δ 7.74 (1H, br. s), 7.49 (1H, s), 7.42–7.45 (2H, m), 7.34–7.38 (2H, m), 7.32–7.28 (1H, m), 7.20 (1H, d, *J* = 9.5 Hz), 6.99 (1H, d, *J* = 2.6 Hz), 6.89 (1H, d, *J* = 9.5, 2.6 Hz), 5.60–5.63 (1H, m), 5.08 (2H, s), 4.46 (1H, d, *J* = 5.3 Hz), 3.62–3.69 (1H, m), 3.61 (3H, s), 3.43 (1H, dt, *J* = 15.6, 5.8 Hz), 3.08–3.12 (1H, m), 2.82–2.89 (1H, m), 2.79 (1H, t, *J* = 5.8 Hz), 2.67–2.79 (2H, m), 2.19–2.24 (1H, m), 1.81 (3H, s); ^13^C NMR (150 MHz, CDCl_3_) δ 168.6, 153.5, 146.5, 139.2, 137.6, 133.7, 131.5, 128.6, 128.5 (2C), 127.8, 127.5 (2C), 127.1 112.5, 111.5, 109.0, 103.7, 101.9, 71.3, 52.6, 51.1, 50.6, 49.9, 38.9, 36.1, 22.0, 15.8; EIMS: *m/z* 440 (M)^+^, 349, 276, 185, 91 (base); HREIMS: *m/z* 440.2076 (M)^+^ (440.2098 calcd. for C_28_H_28_N_2_O_3_).

### Synthesis of Compound 6

Dess–Martin periodinane (15 wt% solution in dichloromethane) (0.18 ml) was added to a solution of compound **2e** (22 mg, 0.048 mmol) in dichloromethane (1.0 ml) at 0°C. After being stirred for 2 h, the reaction mixture was poured into 10% water solution of sodium thiosulfate and extracted with ethyl acetate three times. The combined organic layer was washed with water and brine, dried over sodium sulfate, and concentrated *in vacuo*. The residue was chromatographed over silica gel eluted by hexane-ethyl acetate (2:1) to afford **6** (7.1 mg, 31%). Analytical data for **6**: yellowish oil; (α)_D_^28^–360° (*c* 0.245, CHCl_3_); ^1^H NMR (600 MHz, CDCl_3_) δ 7.85 (1H, br. s), 7.43–7.46 (2H, m), 7.41 (1H, s), 7.35–7.38 (2H, m), 7.28–7.32 (1H, m), 7.21 (1H, d, *J* = 8.7 Hz), 6.98 (1H, d, *J* = 2.3 Hz), 6.90 (1H, dd, *J* = 8.7, 2.3 Hz), 5.07 (2H, s), 4.54–4.57 (1H, m), 3.77 (1H, dd, *J* = 15.3, 5.5 Hz), 3.60 (3H, s), 3.46 (1H, dt, *J* = 15.3, 4.9 Hz), 3.01–3.04 (1H, m), 2.87–2.93 (1H, m), 2.74 (1H, ddd, *J* = 15.1, 3.9, 1.5 Hz), 2.64 (1H, ddd, *J* = 19.4, 3.0, 1.8 Hz), 2.53 (1H, dd, *J* = 19.4, 8.1 Hz), 2.30–2.43 (1H, m), 1.22 (3H, d, *J* = 7.2` Hz); ^13^C NMR (150 MHz, CDCl_3_) δ 219.4, 167.9, 153.6, 145.7, 137.6, 132.7, 131.2, 128.5 (2C), 127.8, 127.6, 127.5 (2C), 112.9, 111.8, 109.4, 102.0, 101.4, 71.0, 52.5, 51.5, 50.8, 45.5, 44.9, 43.7, 28.2, 22.5, 13.5; LREIMS: *m/z* 456 (M)^+^ (base), 397, 365, 333; HREIMS: *m/z* 456.2042 (M)^+^ (456.2047 calcd. for C_28_H_28_N_2_O_4_).

### Synthesis of Compound 7

Sodium borohydride (1.4 mg, 0.037 mmol) was added to a solution of compound **6** (5.0 mg, 0.011 mmol) in methanol (1.0 ml) at 0°C. After being stirred for 2 h, the reaction mixture was poured into 0.5 M hydrochloric acid and extracted with ethyl acetate three times. The combined organic layer was washed with water and brine, dried over sodium sulfate, and concentrated *in vacuo*. The residue was chromatographed over silica gel eluted by hexane-ethyl acetate (1:2) to afford **7** (1.8 mg, 35%). Analytical data for **7**: yellowish oil; (α)_D_^26^–59° (*c* 0.117, CHCl_3_); ^1^H NMR (600 MHz, CDCl_3_) δ 7.92 (1H, br. s), 7.48 (1H, s), 7.45 (2H, d, *J* = 7.7 Hz), 7.36 (2H, t, *J* = 7.7 Hz), 7.30 (1H, t, *J* = 7.7 Hz), 7.21 (1H, d, *J* = 8.7 Hz), 6.99 (1H, d, *J* = 2.5 Hz), 6.89 (1H, d, *J* = 8.7, 2.5 Hz), 5.08 (2H, s), 4.32 (1H, d, *J* = 7.8 Hz), 3.95 (1H, dd, *J* = 14.5, 6.0 Hz), 3.65–3.70 (1H, m), 3.65 (3H, s), 3.51 (1H, dt, *J* = 14.5, 5.1 Hz), 2.90–2.94 (1H, m), 2.82–2.88 (1H, m), 2.69–2.72 (1H, m), 2.63 (1H, dt, *J* = 13.5, 6.7 Hz), 2.10–2.13 (1H, m), 1.82 (1H, dt, *J* = 7.4, 4.8 Hz), 1.47 (1H, ddd, *J* = 15.1, 7.6, 5.3 Hz), 1.19 (3H, d, *J* = 8.4 Hz); ^13^C NMR (150 MHz, CDCl_3_) δ 168.8, 153.5, 145.8, 137.6, 133.7, 131.3, 128.5 (2C), 127.7, 127.5 (2C), 127.1, 112.7, 111.6, 109.5, 101.9, 99.9, 79.3, 71.0, 53.2, 51.6, 50.6, 48.0, 45.7, 42.7, 30.0, 22.2, 18.8; EIMS: *m/z* 458 (M)^+^ (base), 399, 385, 91, 44; HREIMS: *m/z* 458.2209 (M)^+^ (458.2204 calcd. for C_28_H_30_N_2_O_4_).

### Synthesis of Compound 9

*N*-Boc-5-hydroxy-L-tryptophan methyl ester (**8**) (Zhu et al., 2015) (481 mg, 1.44 mmol) was dissolved in acetone (5 ml), and cesium carbonate (639 mg, 1.96 mmol) and 4-nitrobenzyl bromide (390 mg, 1.80 mmol) were added to this solution at 0°C. After being stirred for 2 h at 0°C, the reaction mixture was poured into saturated ammonium chloride solution and extracted with ethyl acetate three times. The combined organic layer was washed with water and brine, dried over sodium sulfate, and concentrated *in vacuo*. The residue was chromatographed over silica gel eluted by hexane-ethyl acetate (1:2) to afford *N*-Boc-5-(*p*-nitrobenzyl)oxy-L-tryptophan methyl ester (622 mg, 92%).

*N*-Boc-5-(*p*-nitrobenzyl)oxy-L-tryptophan methyl ester (397 mg, 0.846 mmol) was dissolved in methanol (1.5 ml), and hydrogen chloride-methanol reagent (Tokyo Chemical Industry Co., Ltd.) (1.5 ml) was added to this solution. After being stirred for 15 h at room temperature, the reaction mixture was poured into saturated sodium bicarbonate solution and extracted with ethyl acetate three times. The combined organic layer was washed with water and brine, dried over sodium sulfate, and concentrated *in vacuo*. The residue was chromatographed over silica gel eluted by chloroform-methanol (19:1) to afford **9** (250 mg, 80%). Analytical data for **9**: yellowish amorphous solid; ^1^H NMR (400 MHz, CDCl_3_) δ 8.23 (2H, d, *J* = 8.7 Hz), 8.19 (1H, s), 7.64 (2H, d, *J* = 8.7 Hz), 7.27 (1H, d, *J* = 8.6 Hz), 7.13 (1H, d, *J* = 2.5 Hz), 7.05 (1H, s), 6.93 (1H, dd, *J* = 8.6, 2.5 Hz), 5.20 (2H, s), 3.79 (1H, dd, *J* = 7.5, 5.0 Hz), 3.70 (3H, s), 3.21 (1H, dd, *J* = 14.3, 5.0 Hz), 3.02 (1H, dd, *J* = 14.3, 7.5 Hz); ^13^C NMR (100 MHz, CDCl_3_) δ 177.1, 154.0, 148.8, 146.6, 133.2, 129.4, 129.1 (2C), 125.4, 125.1 (2C), 114.1, 113.5, 112.4, 103.8, 71.0, 56.4, 53.5, 31.9; EIMS: *m/z* 369 (M)^+^ (base), 281, 145; HREIMS: *m/z* 369.1279 (M)^+^ (369.1323 calcd. for C_19_H_19_N_3_O_5_).

### Synthesis of Compound 10

*N*-Boc-5-hydroxy-L-tryptophan methyl ester (**8**) (Zhu et al., 2015) (499 mg, 1.49 mmol) was dissolved in THF (6 ml), and this solution was added dropwise to the dispersion of lithium aluminum hydride (138 mg, 3.67 mmol) in THF (4 ml) at 0°C. After being stirred for 30 min at room temperature, the reaction mixture was poured into 1 M citric acid solution and extracted with ethyl acetate three times. The combined organic layer was washed with water and brine, dried over sodium sulfate, and concentrated *in vacuo*. The residue was chromatographed over silica gel eluted by hexane-ethyl acetate (1:4) to afford **10** (392 mg, 86%). Analytical data for **10**: colorless oil; ^1^H NMR (400 MHz, methanol-*d*_4_) δ 7.14 (1H, d, *J* = 8.6 Hz), 6.98–7.00 (1H, m), 6.64 (1H, d, *J* = 8.6 Hz), 3.84 (1H, br. s), 3.51 (2H, br. s), 2.78–2.89 (2H, m), 1.39 (9H, s); ^13^C NMR (100 MHz, methanol-*d*_4_) δ 158.2, 151.0, 133.0, 129.7, 124.9, 112.6, 112.3, 111.7, 103.8, 79.9, 64.5, 54.5, 28.8 (3C), 28.1; EIMS: *m/z* 306 (M)^+^, 146 (base); HREIMS: *m/z* 306.1562 (M)^+^ (306.1578 calcd. for C_16_H_22_N_2_O_4_).

### Synthesis of Compound 11

Compound **10** (200 mg, 0.653 mmol) was dissolved in acetone (3 ml), and cesium carbonate (280 mg, 0.862 mmol) and 4-nitrobenzyl bromide (170 mg, 0.787 mmol) were added to this solution at 0°C. After being stirred for 3 h at 0°C, the reaction mixture was poured into saturated ammonium chloride solution and extracted with ethyl acetate three times. The combined organic layer was washed with water and brine, dried over sodium sulfate, and concentrated *in vacuo*. The residue was chromatographed over silica gel eluted by hexane-ethyl acetate (1:3) to afford *N*-Boc-5-(*p*-nitrobenzyl)oxy-L-tryptophanol (254 mg, 88%).

*N*-Boc-5-(*p*-nitrobenzyl)oxy-L-tryptophanol (86 mg, 0.194 mmol) was dissolved in acetonitrile (1 ml), and bismuth (III) chloride (Navath et al., 2006) (70 mg, 0.221 mmol) and water (20 μL) were added to this solution. After being stirred for 18 h at room temperature, the reaction mixture was poured into saturated sodium bicarbonate solution and extracted with ethyl acetate three times. The combined organic layer was washed with water and brine, dried over sodium sulfate, and concentrated *in vacuo*. The residue was chromatographed over silica gel eluted by chloroform-methanol (19:1) to afford **11** (51 mg, 83%). Analytical data for **11**: yellowish amorphous solid; ^1^H NMR (400 MHz, methanol-*d*_4_) δ 8.17 (2H, d, *J* = 8.5 Hz), 7.63 (2H, d, *J* = 8.5 Hz), 7.25 (1H, d, *J* = 8.7 Hz), 7.05–7.11 (2H, m), 6.80–6.89 (1H, m), 5.19 (2H, s), 3.70–3.79 (2H, m), 3.37 (1H, t, *J* = 7.7 Hz), 2.99–3.05 (1H, m), 2.82–2.87 (1H, m); ^13^C NMR (100 MHz, methanol-*d*_4_) δ 153.8, 148.8, 146.6, 133.2, 129.3, 129.0 (2C), 125.1 (2C), 124.8, 114.2, 113.6, 113.3, 102.6, 71.9, 71.0, 59.6, 30.3; HRFABMS: *m/z* 342.1429 (M + H)^+^ (341.1374 calcd. for C_18_H_19_N_3_O_4_).

### Preparation of Compounds 12 and 13 by Using the Diversity-Enhanced Extracts

The mixture of iridoids (75 mg) was dissolved in dichloromethane (4.5 ml), and compound **9** (101 mg, 0.273 mmol) and bismuth (III) trifluoromethanesulfonate (18 mg, 0.027 mmol) were added to the solution. After being stirred for 5 h at room temperature, the reaction mixture was poured into saturated sodium bicarbonate solution and extracted with ethyl acetate three times. The combined organic layer was washed with water and brine, dried over sodium sulfate, and concentrated *in vacuo* to give diversity-enhanced extracts (179 mg). They were chromatographed over silica gel and the column was eluted with chloroform-methanol mixtures with increasing polarity to afford chloroform-methanol (19:1) eluent (46 mg), which was separated by ODS column using water-acetonitrile solvent system to give water-acetonitrile (3:7) eluent (32 mg). It was subjected to recycle preparative HPLC (column, YMC-GPC T-2000 (ϕ 20 mm × 600 mm, TMC Co., Ltd.); solvent, ethyl acetate) to give compound **12** (18 mg, 20% (w/w) from the mixture of iridoids). Analytical data for **12**: yellowish oil; (α)_D_^26^–16° (*c* 0.640, CHCl_3_); ^1^H NMR (400 MHz, CDCl_3_) δ 8.24 (2H, d, *J* = 8.1 Hz), 7.97 (1H, s), 7.64 (2H, d, *J* = 8.1 Hz), 7.44 (1H, s), 7.24 (1H, d, *J* = 8.8 Hz), 6.99 (1H, d, *J* = 2.3 Hz), 6.89 (1H, dd, *J* = 8.8, 2.3 Hz), 5.21 (2H, s), 4.40–4.42 (2H, m), 4.25–4.28 (1H, br), 3.68 (3H, s), 3.62 (3H, s), 3.32 (1H, d, *J* = 15.4 Hz), 3.11–3.24 (2H, m), 2.39 (1H, dd, *J* = 13.8, 6.7 Hz), 2.20–2.29 (1H, m), 1.94–2.00 (1H, m), 1.63–1.67 (1H, m), 1.22 (3H, d, *J* = 7.1 Hz); ^13^C NMR (100 MHz, CDCl_3_) δ 171.4, 168.7, 152.9, 145.7, 145.2, 133.5, 131.7, 127.6 (2C), 127.1, 123.7 (2C), 112.5, 111.8, 106.9, 102.7, 102.2, 80.4, 73.5, 69.5, 62.5, 52.5, 51.2, 50.8, 46.8, 43.3, 43.0, 33.5, 24.0, 13.9; EIMS: *m/z* 561 (M)^+^, 425, 281, 44 (base); HREIMS: *m/z* 561.2089 (M)^+^ (561.2109 calcd. for C_30_H_31_N_3_O_8_).

Using the procedure described above, compound **13** (6.1 mg, 13% (w/w) from the mixture of iridoids) was synthesized from the mixture of iridoids and compound **12**. Analytical data for **13**: yellowish oil; (α)_D_^26^–112° (*c* 0.307, CHCl_3_); ^1^H NMR (600 MHz, CDCl_3_) δ 8.21 (2H, d, *J* = 8.7 Hz), 8.07 (1H, s), 7.60 (2H, d, *J* = 8.7 Hz), 7.36 (1H, s), 7.23 (1H, d, *J* = 8.8 Hz), 6.91 (1H, d, *J* = 2.4 Hz), 6.88 (1H, dd, *J* = 8.8, 2.4 Hz), 5.17 (2H, s), 4.26 (1H, br. d, *J* = 4.9 Hz), 4.20 (1H, t, *J* = 4.7 Hz), 3.87–3.90 (1H, m), 3.73 (1H, t, *J* = 10.4 Hz), 3.62–3.65 (1H, m), 3.61 (3H, s), 2.99–3.05 (2H, m), 2.58 (1H, d, *J* = 15.6 Hz), 2.20–2.30 (2H, m), 2.07–2.12 (1H, m), 1.70–1.74 (1H, m), 1.18 (3H, d, *J* = 7.2 Hz); ^13^C NMR (150 MHz, CDCl_3_) δ 168.6, 152.8, 147.5, 145.9, 145.2, 133.1, 131.5, 127.7, 127.6 (2C), 123.7 (2C), 112.4, 111.9, 106.9, 104.4, 101.9, 74.1, 69.7, 61.8 (2C), 50.8, 48.1, 44.9, 42.6, 41.8, 31.9, 22.6, 13.0; LREIMS: *m/z* 533 (M)^+^, 398, 367 (base), 44; HREIMS: *m/z* 533.2167 (M)^+^ (533.2160 calcd. for C_29_H_31_N_3_O_7_).

### Luciferase Assay of HEK293/CTLA-4luc

HEK293 cells derived from human embryonic kidney were purchased from American Type Culture Collection (ATCC) and maintained in Dulbecco’s modified Eagle’s medium (DMEM, Invitrogen) supplemented with 10% fetal bovine serum (FBS) and 50 unit/mL penicillin and streptomycin in a 5% CO_2_ humidified atmosphere. The cell line, HEK293/CTLA-4luc, was established by co-transfection of HEK293 cells with pLightSwitch CTLA-4-luc (SwitchGear Genomics, product ID S700692) and pPUR (Clontech), followed by selection in the presence of 1 µg/ml puromycin (Sigma). HEK293/CTLA-4luc was cultured with synthesized compounds. After 48 h, the cell lysate was prepared for luciferase assay with Luciferase Reporter Assay System (Promega) according to the manufacturer’s instruction. Luciferase activities were measured using a GloMax^®^ 20/20 Luminometer (Promega). Simultaneously, the cell lysate was used to determine protein level by BCA protein assay kit (Thermo Scientific) according to the manufacture’s instruction. The relative luciferase (RLU) activity indicated in this article was normalized to total protein content and cell viability.

### Luciferase Assay of A549/PD-L1luc

A549 cells derived from human lung adenocarcinoma were purchased from American Type Culture Collection (ATCC) and maintained in Dulbecco’s modified Eagle’s medium (DMEM, Invitrogen) supplemented with 10% fetal bovine serum (FBS) and 50 unit/mL penicillin and streptomycin in a 5% CO_2_ humidified atmosphere. The cell line, A549/PD-L1luc, was established by co-transfection of A549 cells with pPD-L1luc and pPUR (Clontech), followed by selection in the presence of 1 µg/ml puromycin (Sigma). A549/PD-L1luc was cultured with synthesized compounds. After 48 h, the cell lysate was prepared for luciferase assay with Luciferase Reporter Assay System (Promega) according to the manufacturer’s instruction. Luciferase activities were measured using a GloMax^®^ 20/20 Luminometer (Promega). Simultaneously, the cell lysate was used to determine protein level by BCA protein assay kit (Thermo Scientific) according to the manufacture’s instruction. The relative luciferase (RLU) activity indicated in this article was normalized to total protein content and cell viability.

### Flow Cytometric Analysis for CTLA-4 Expression

Cell suspensions of MT-2 cells were prepared and washed in fluorescence-activated cell sorter buffer consisting of phosphate-buffered saline (PBS) containing 1% bovine serum albumin (BSA). After blocking with normal mouse serum, cells were incubated with a fluorochrome (PE) labeled anti-CD152 antibody (Beckman Coulter, IM2282) or mouse IgG2a isotype control (Beckman Coulter, A09142) in the dark for 30 min ice bath. After fixation, fluorescent signals from cells were acquired on a Cell Sorter SH800 flow cytometer (SONY) and the data were analyzed using FlowJo software (BD Biosciences).

### Flow Cytometric Analysis for PD-L1 Expression

Cell suspensions of THP-1 cells were prepared and washed in fluorescence-activated cell sorter buffer consisting of phosphate-buffered saline (PBS) containing 1% bovine serum albumin (BSA). After blocking with normal mouse serum, cells were incubated with a fluorochrome (PC7) labeled anti-CD274 antibody (Beckman Coulter, A78884) or mouse IgG1 isotype control (Beckman Coulter, 737,662) in the dark for 30 min ice bath. After fixation, fluorescent signals from cells were acquired on a Cell Sorter SH800 flow cytometer (SONY) and the data were analyzed using FlowJo software (BD Biosciences).

## Data Availability

The original contributions presented in the study are included in the article/Supplementary Material; further inquiries can be directed to the corresponding author.
