# Supplementary material for: Development of Indole Alkaloid-Type Dual Immune Checkpoint Inhibitors Against CTLA-4 and PD-L1 Based on Diversity-Enhanced Extracts
Source: Front Chem. 2021 Nov 8;9:766107. doi: 10.3389/fchem.2021.766107 (PMC8630621; doi:10.3389/fchem.2021.766107)
Supplement: Supplementary file 1 [file DataSheet1.PDF]

Supplementary Material

Development of Indole Alkaloid-Type Dual Immune Checkpoint Inhibitors against CTLA-4 and PD-L1 Based on Diversity-Enhanced Extracts

Yoshihide Suzuki,[†] Keisuke Ichinohe,[†] Akihiro Sugawara,[†] Shinya Kida,[‡] Shinya Murase,[‡] Jing Zhang,[‡] Osamu Yamada,[‡] Toshio Hattori,[§] Yoshiteru Oshima[†] and Haruhisa Kikuchi,[*,†,||]

[†]*Graduate School of Pharmaceutical Sciences, Tohoku University, 6-3, Aza-Aoba, Aramaki, Aoba-ku, Sendai 980-8578, Japan.*

[‡]*Research and Development Center, FUSO Pharmaceutical Industries, LTD., 2-3-30 Morinomiya, Joto-ku, Osaka 536-8523, Japan*

[§]*Department of Health Science and Social Welfare, Kibi International University, 8 Igamachi, Takahashi 716-8508, Japan*

[||] *Division of Natural Medicines, Faculty of Pharmacy, Keio University, 1-5-30, Shibakoen, Minato-ku, Tokyo 105-8512, Japan*

^{1}H NMR (600 MHz, $CDCl_3$)

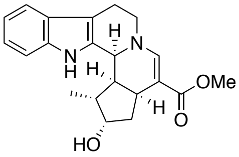

Compound **1**

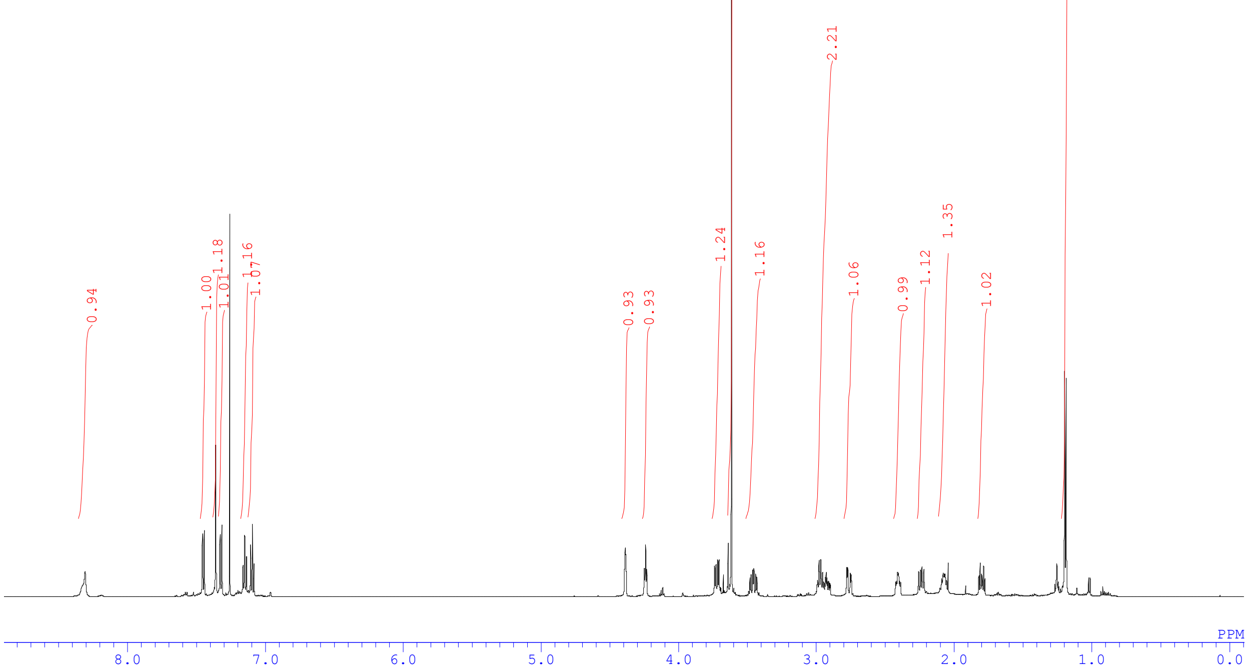

^{13}C NMR (150 MHz, $CDCl_3$)

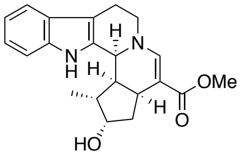

Compound **1**

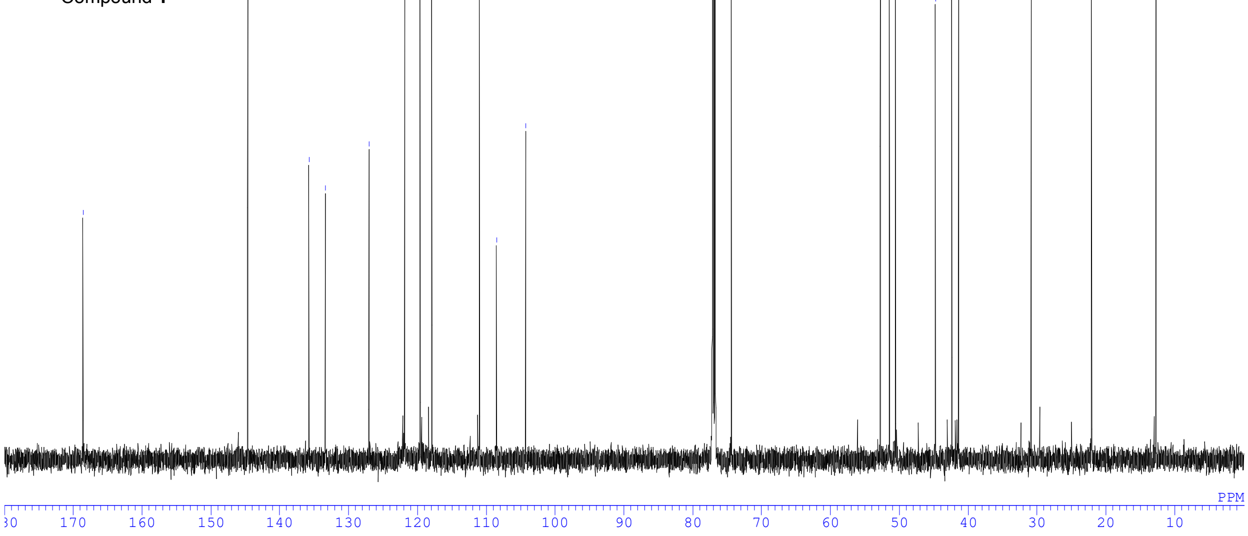

^{1}H NMR (600 MHz, $CDCl_3$)

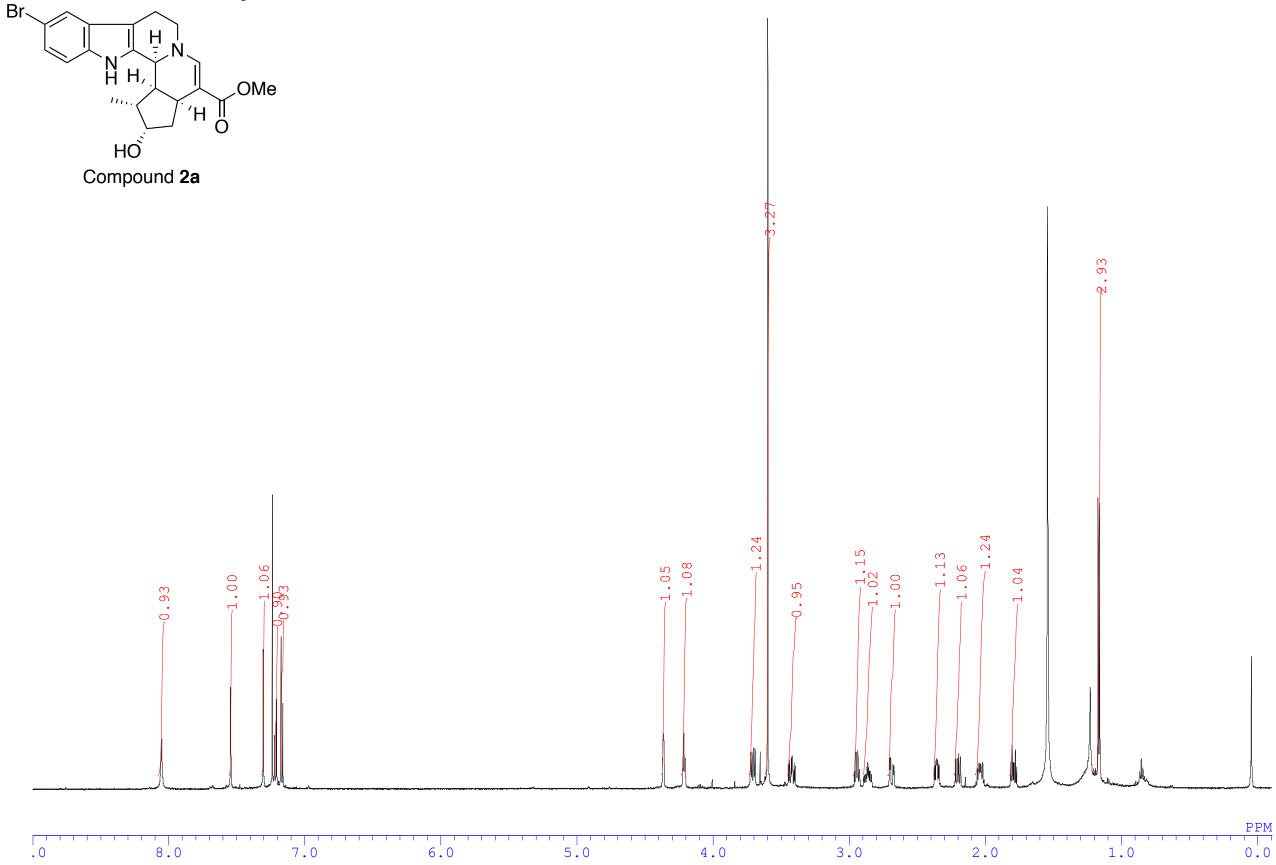

Compound **2a**

^{13}C NMR (150 MHz, $CDCl_3$)

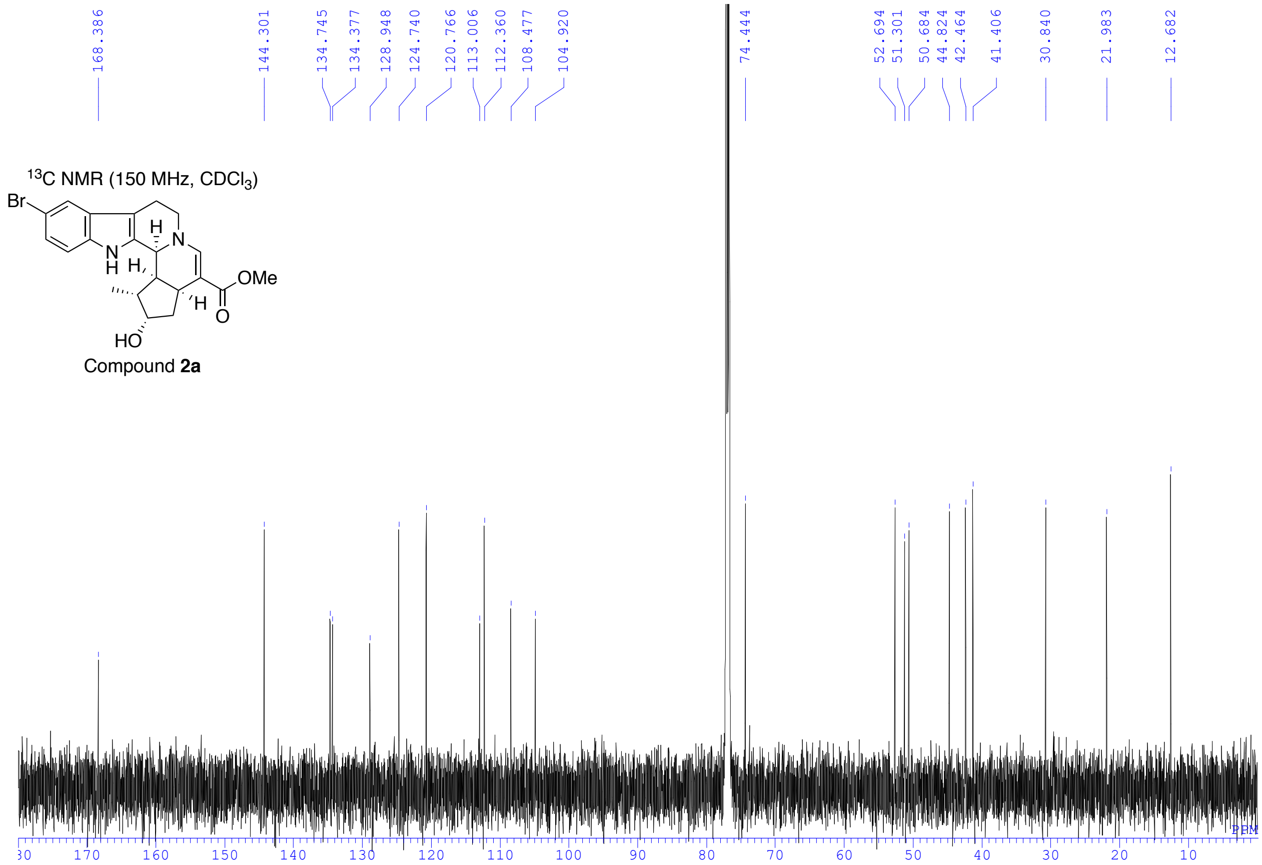

Compound **2a**

^{1}H NMR (600 MHz, $CDCl_3$)

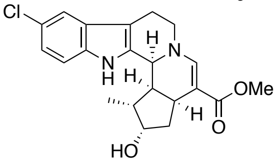

Compound **2b**

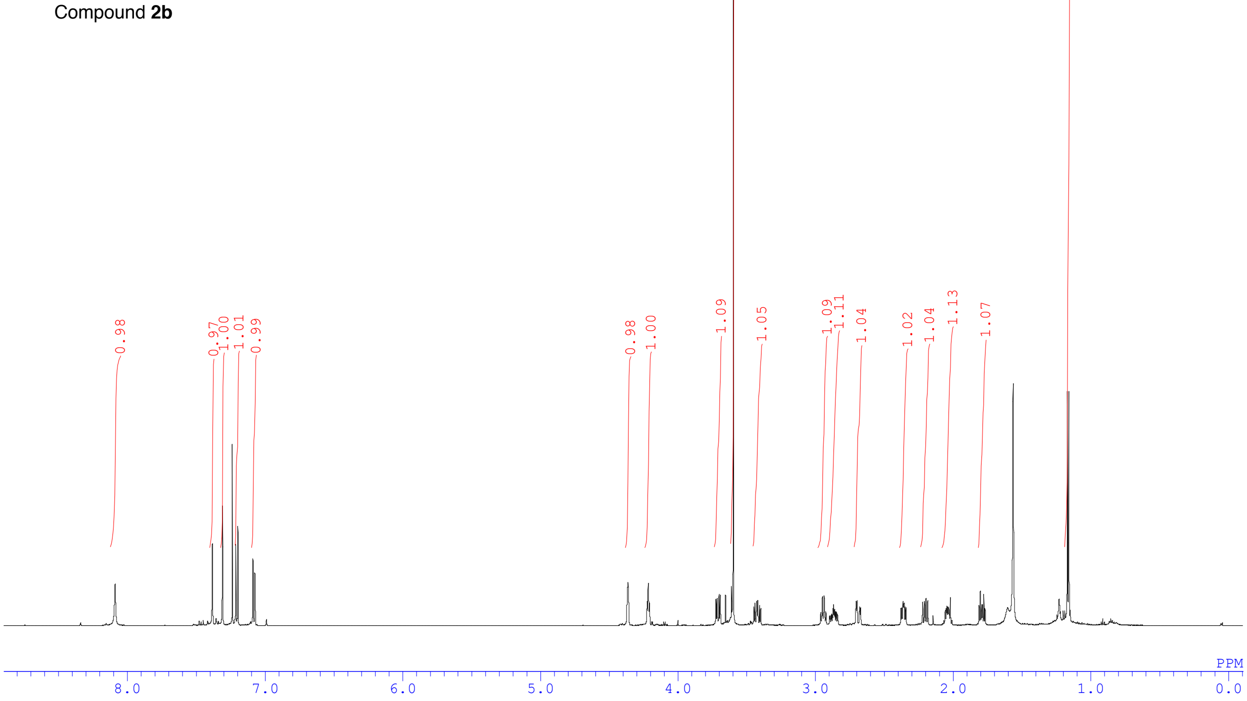

^{13}C NMR (150 MHz, $CDCl_3$)

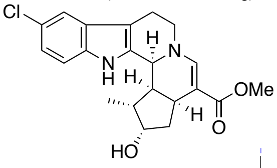

Compound **2b**

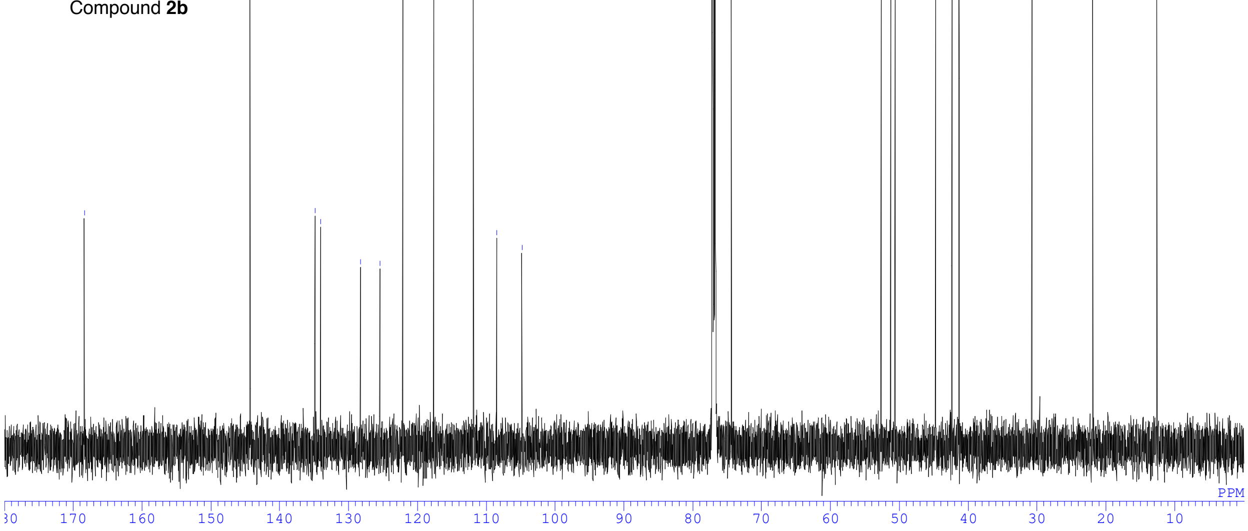

^{1}H NMR (600 MHz, $CDCl_3$)

Compound **2c**

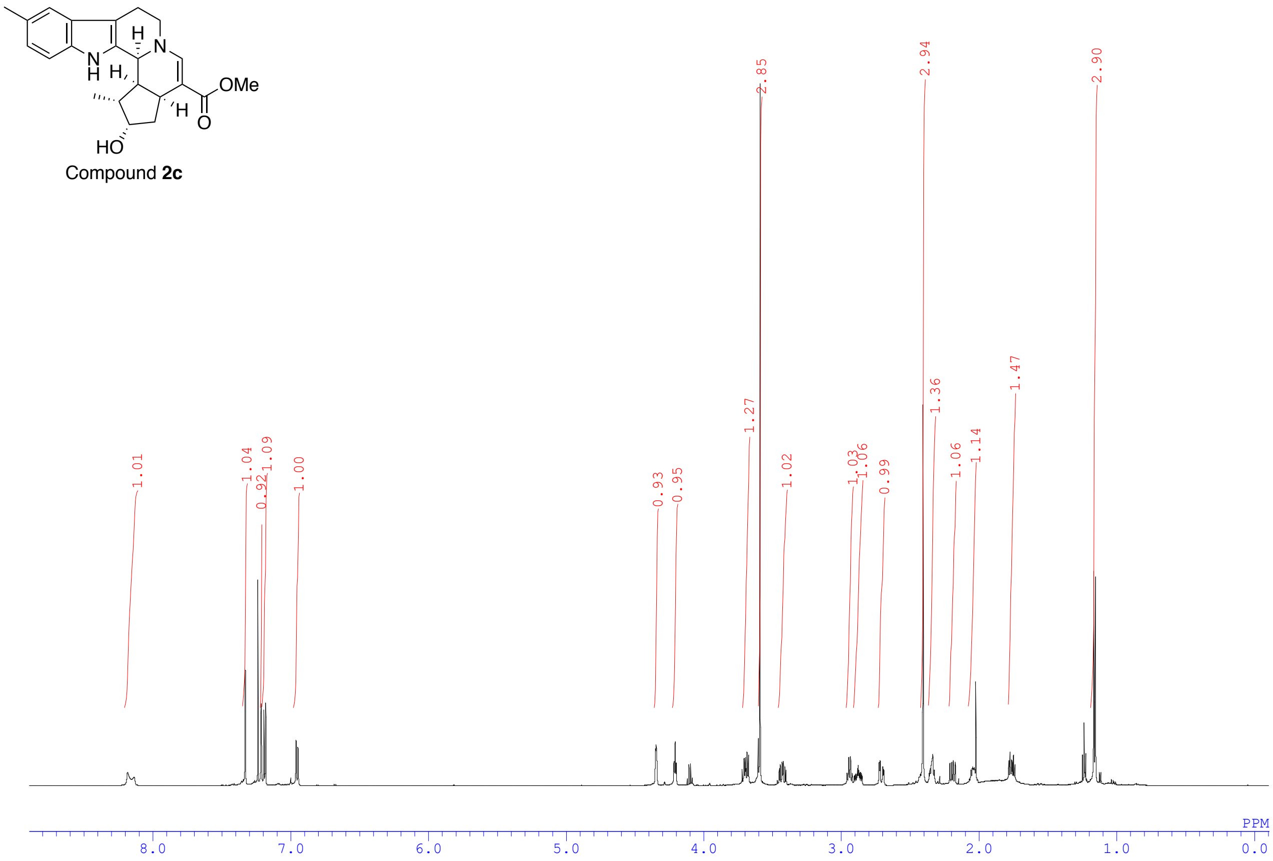

^{13}C NMR (150 MHz, $CDCl_3$)

Compound **2c**

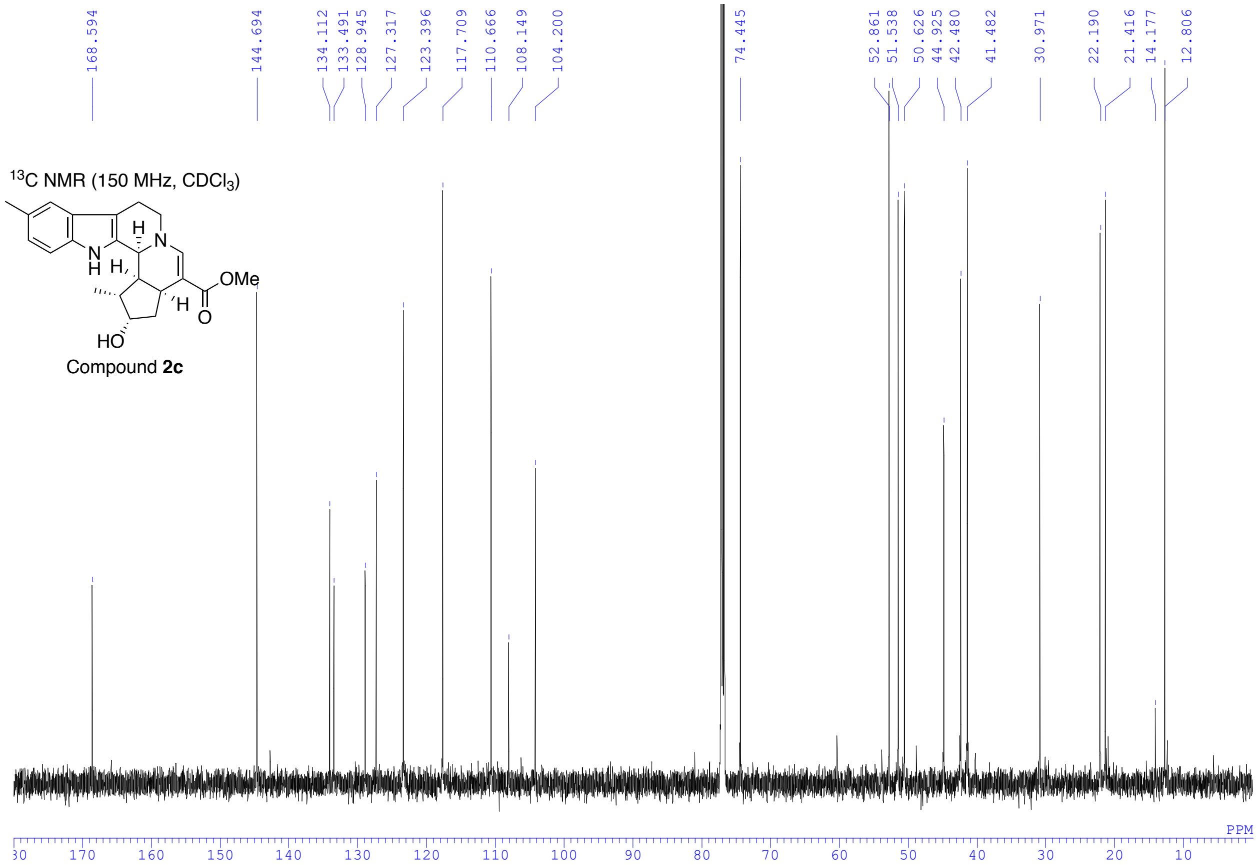

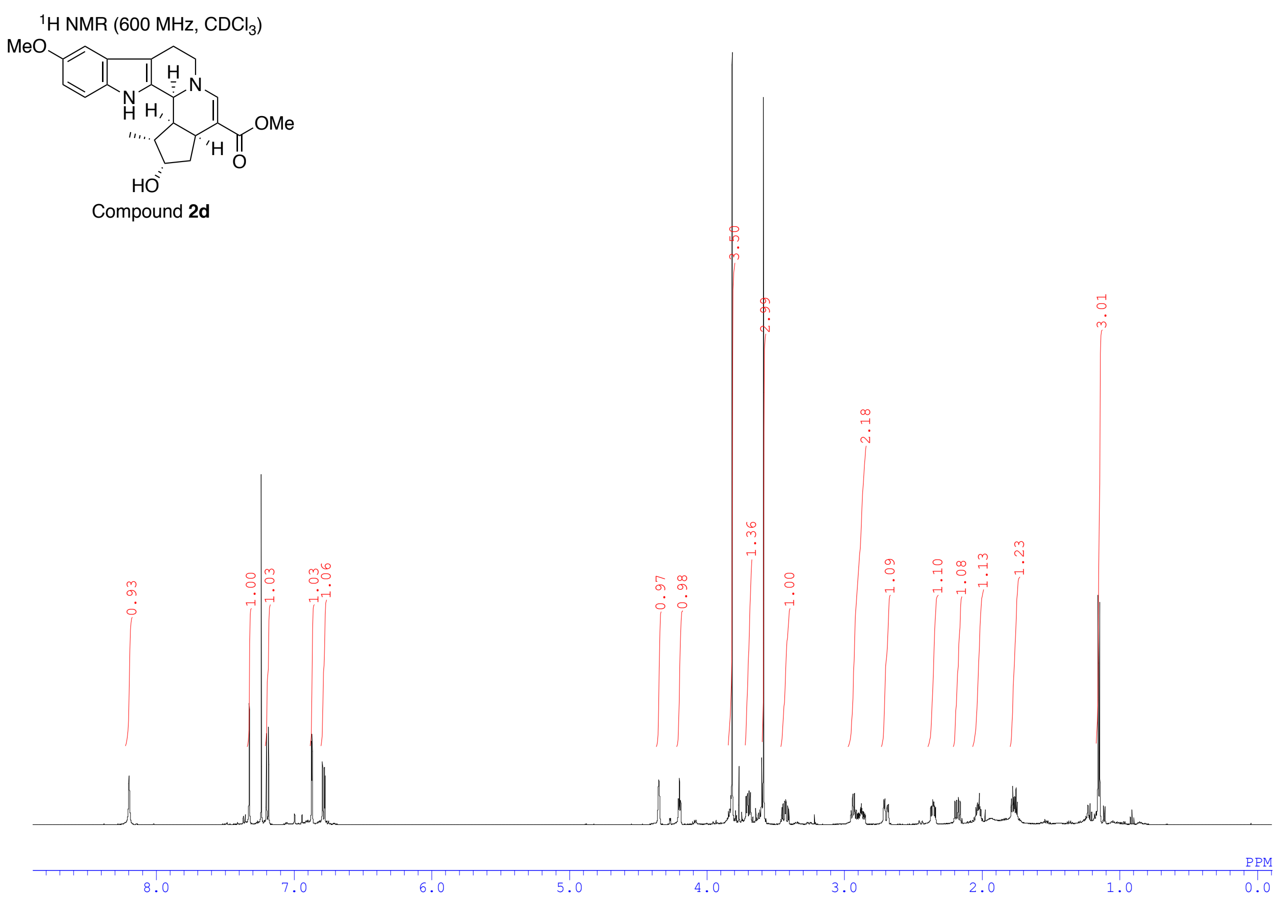
^{1}H NMR (600 MHz, $CDCl_3$)
MeO
N
H
H
H
N
OMe
O
H
HO
Compound **2d**
0.93
1.00
1.03
1.03
1.06
0.97
0.98
3.50
1.36
2.99
1.00
2.18
1.09
1.10
1.08
1.13
1.23
3.01
PPM
8.0
7.0
6.0
5.0
4.0
3.0
2.0
1.0
0.0

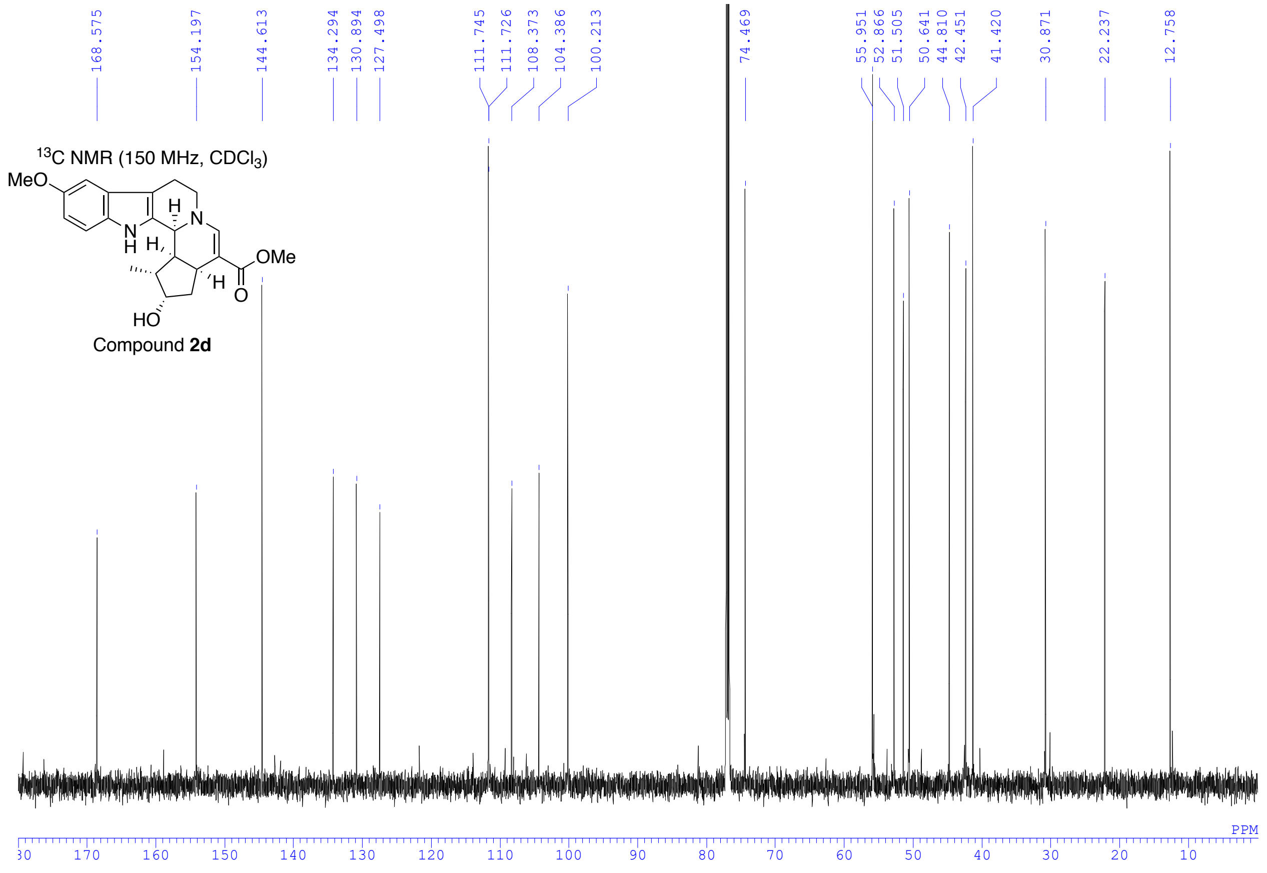
^{13}C NMR (150 MHz, $CDCl_3$)
MeO
N
H
H
H
N
OMe
O
H
HO
Compound **2d**
168.575
154.197
144.613
134.294
130.894
127.498
111.745
111.726
108.373
104.386
100.213
74.469
55.951
52.866
51.505
50.641
44.810
42.451
41.420
30.871
22.237
12.758
PPM
170
160
150
140
130
120
110
100
90
80
70
60
50
40
30
20
10

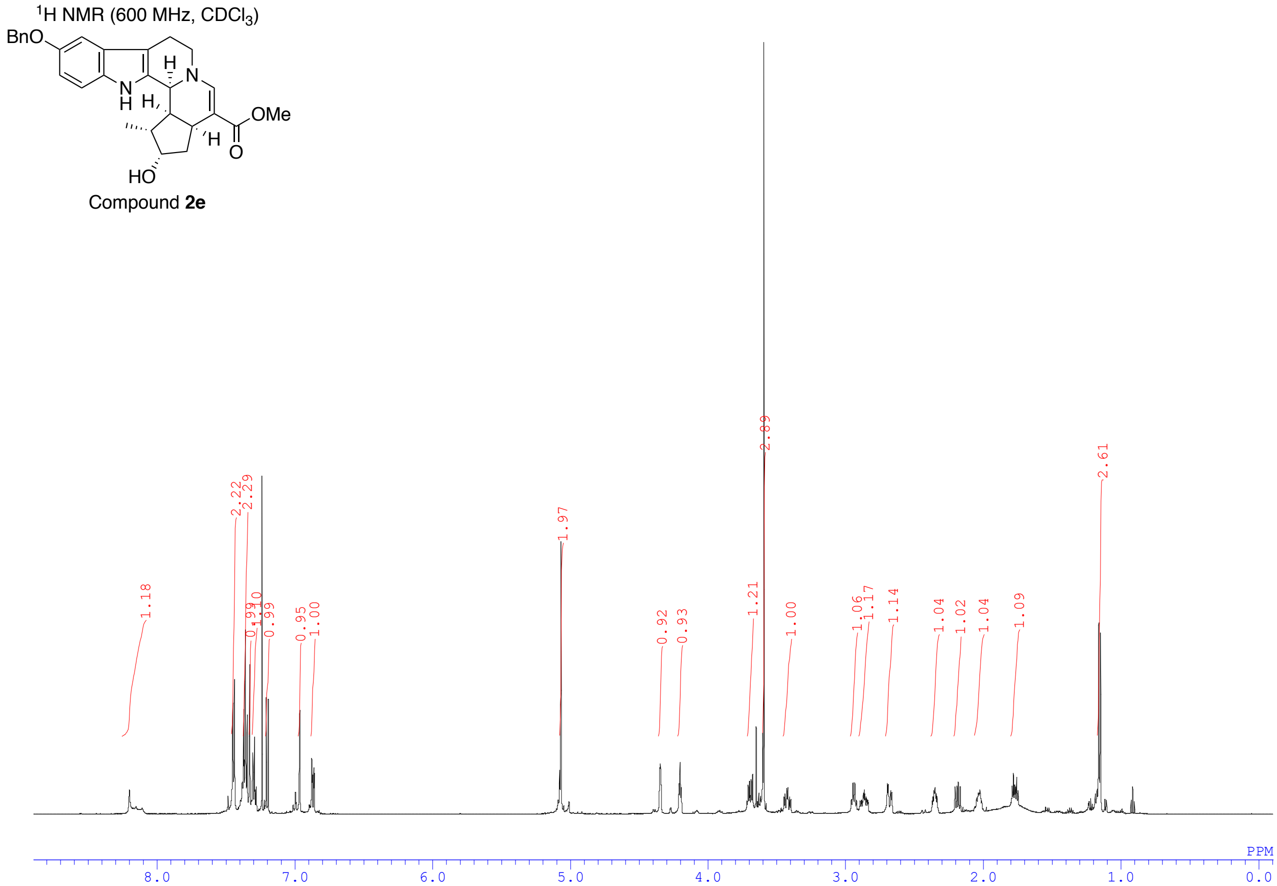

^{1}H NMR (600 MHz, $CDCl_3$)
BnO
OMe
HO
Compound **2e**
1.18
2.22
2.29
0.99
0.99
0.95
1.00
1.97
0.92
0.93
1.21
2.89
1.00
1.06
1.17
1.14
1.04
1.02
1.04
1.09
2.61
PPM
8.0
7.0
6.0
5.0
4.0
3.0
2.0
1.0
0.0

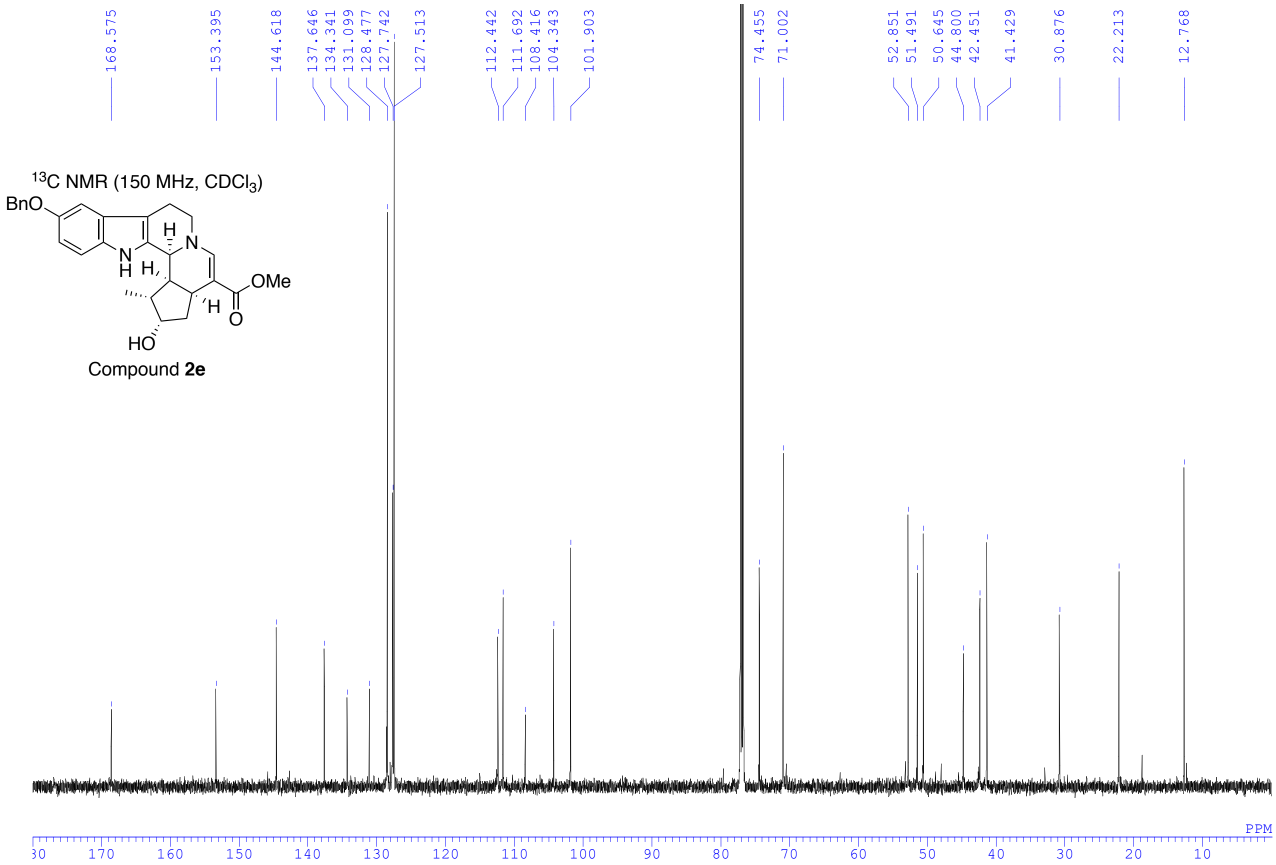

168.575
153.395
144.618
137.646
134.341
131.099
128.477
127.742
127.513
112.442
111.692
108.416
104.343
101.903
74.455
71.002
52.851
51.491
50.645
44.800
42.451
41.429
30.876
22.213
12.768
^{13}C NMR (150 MHz, $CDCl_3$)
BnO
OMe
HO
Compound **2e**
PPM
170
160
150
140
130
120
110
100
90
80
70
60
50
40
30
20
10

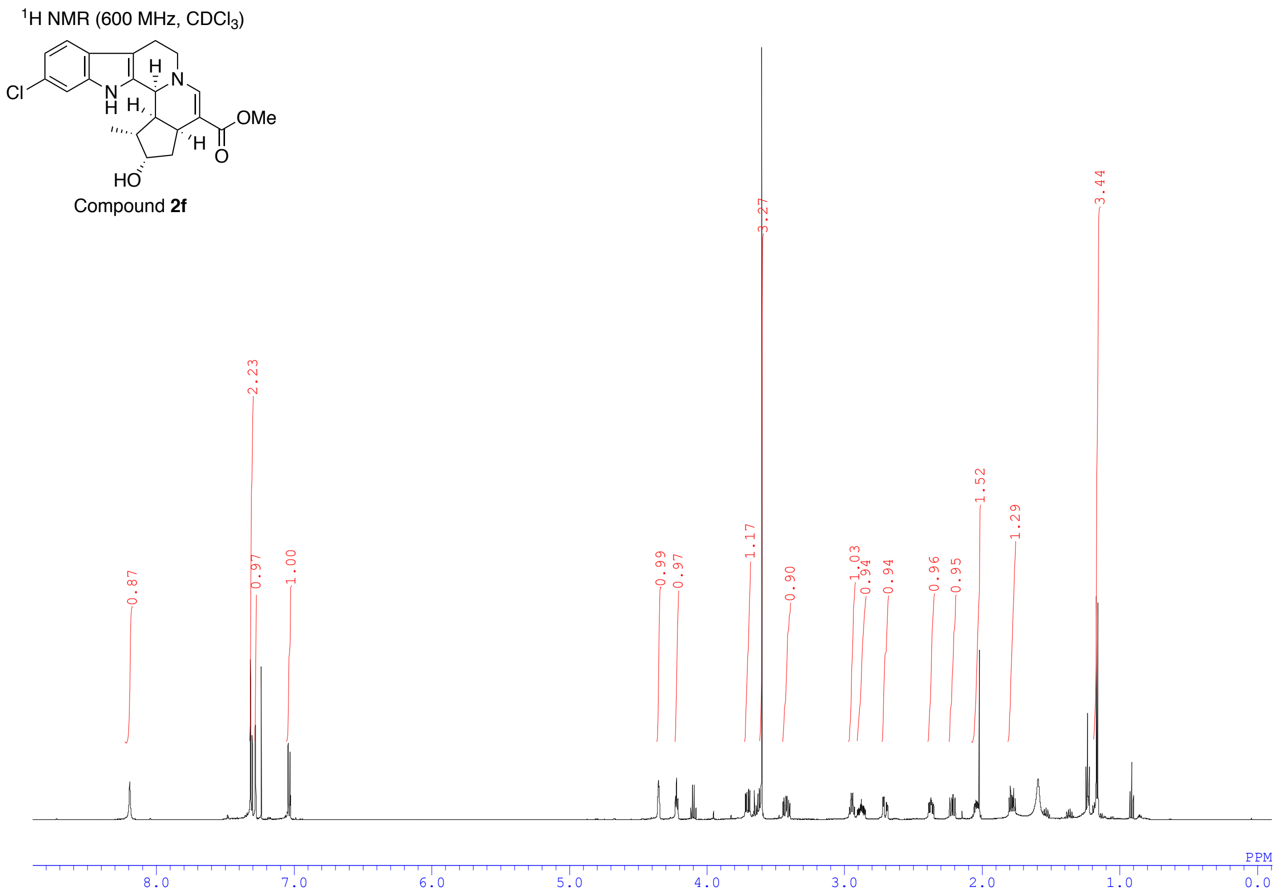
^{1}H NMR (600 MHz, CDCl$_3$)
Cl
N
H
H
H
N
OMe
O
H
HO
Compound **2f**
0.87
2.23
0.97
1.00
0.99
0.97
1.17
3.27
0.90
1.03
0.94
0.94
0.96
0.95
1.52
1.29
3.44
PPM
8.0
7.0
6.0
5.0
4.0
3.0
2.0
1.0
0.0

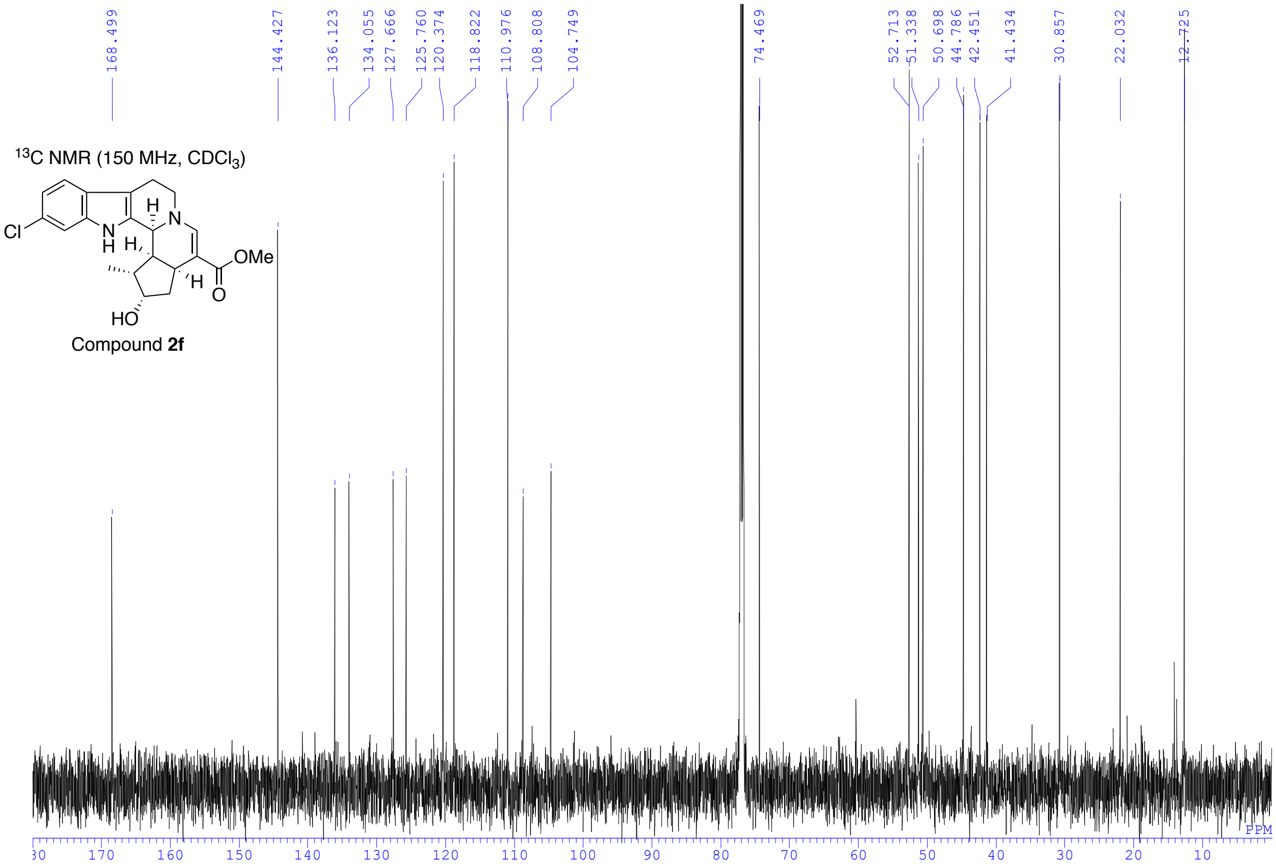
^{13}C NMR (150 MHz, CDCl$_3$)
Cl
N
H
H
H
N
OMe
O
H
HO
Compound **2f**
168.499
144.427
136.123
134.055
127.666
125.760
120.374
118.822
110.976
108.808
104.749
74.469
52.713
51.338
50.698
44.786
42.451
41.434
30.857
22.032
12.725
PPM
170
160
150
140
130
120
110
100
90
80
70
60
50
40
30
20
10

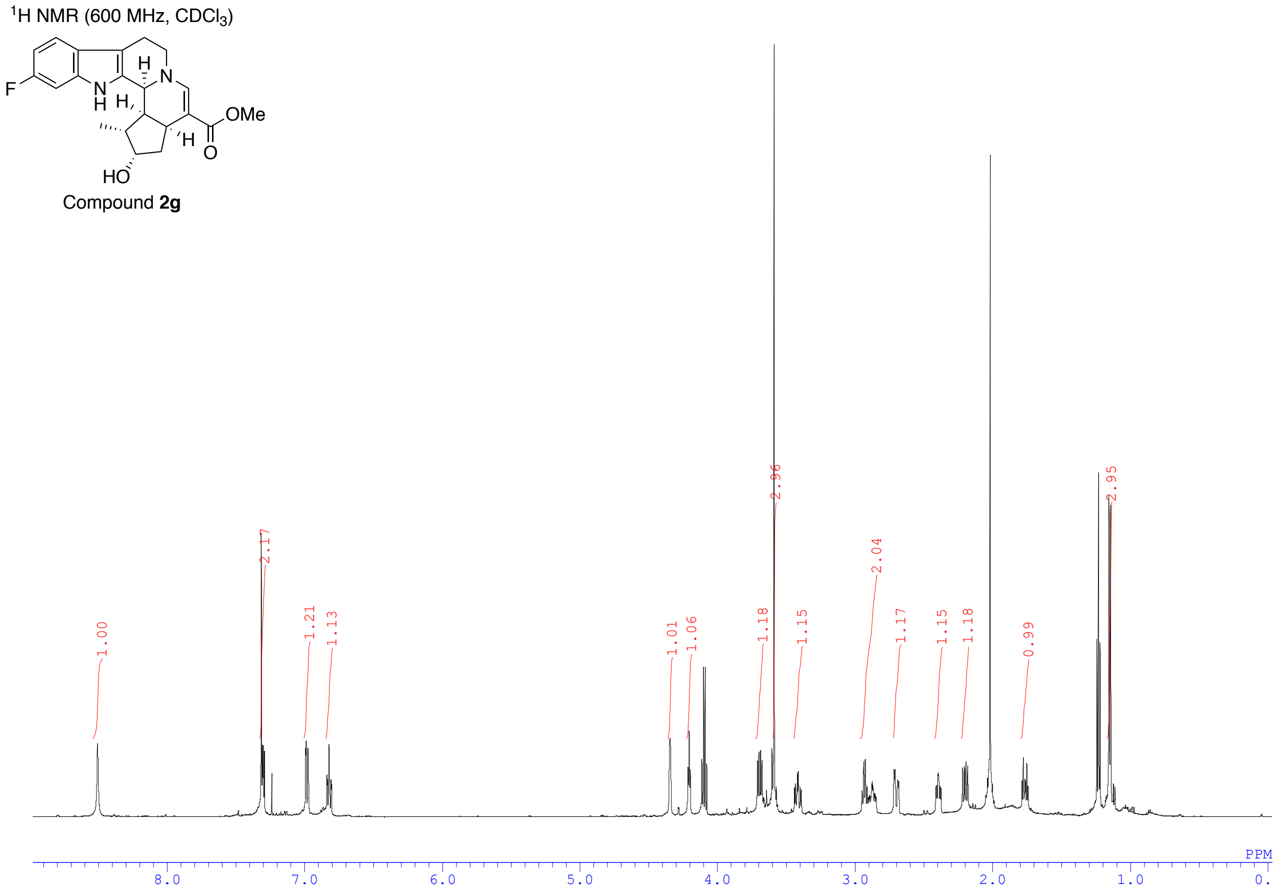
1H NMR (600 MHz, CDCl3)
F
N
H
H
N
H
OMe
H
O
HO
Compound 2g
1.00
2.17
1.21
1.13
1.01
1.06
1.18
2.96
1.15
2.04
1.17
1.15
1.18
0.99
2.95
PPM
8.0
7.0
6.0
5.0
4.0
3.0
2.0
1.0
0.

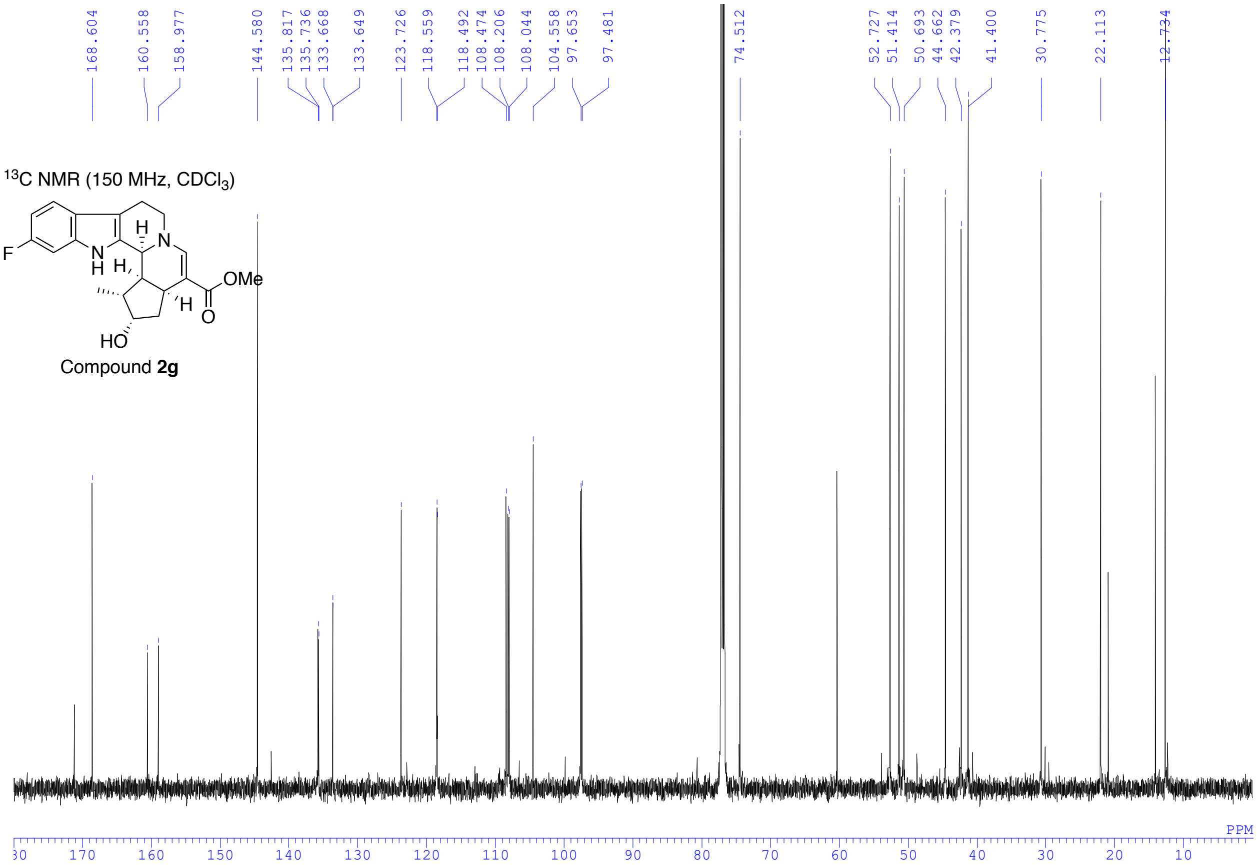
168.604
160.558
158.977
144.580
135.817
135.736
133.668
133.649
123.726
118.559
118.492
108.474
108.206
108.044
104.558
97.653
97.481
74.512
52.727
51.414
50.693
44.662
42.379
41.400
30.775
22.113
12.734
13C NMR (150 MHz, CDCl3)
F
N
H
H
N
H
OMe
H
O
HO
Compound 2g
PPM
30
170
160
150
140
130
120
110
100
90
80
70
60
50
40
30
20
10

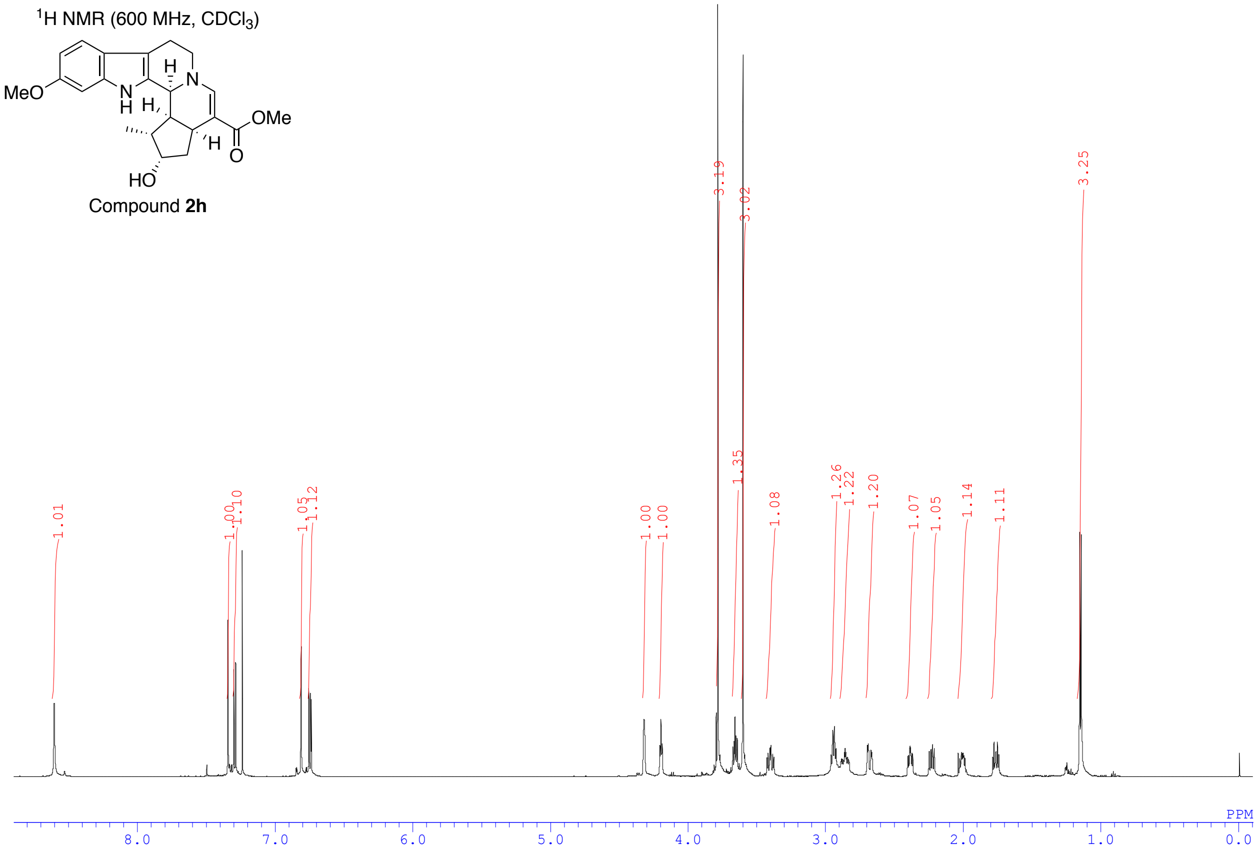

1H NMR (600 MHz, CDCl3)
MeO
H
N
H
OMe
O
HO
Compound 2h
1.01
1.00
1.10
1.05
1.12
1.00
1.00
3.19
1.35
3.02
1.08
1.26
1.22
1.20
1.07
1.05
1.14
1.11
3.25
PPM
8.0
7.0
6.0
5.0
4.0
3.0
2.0
1.0
0.0

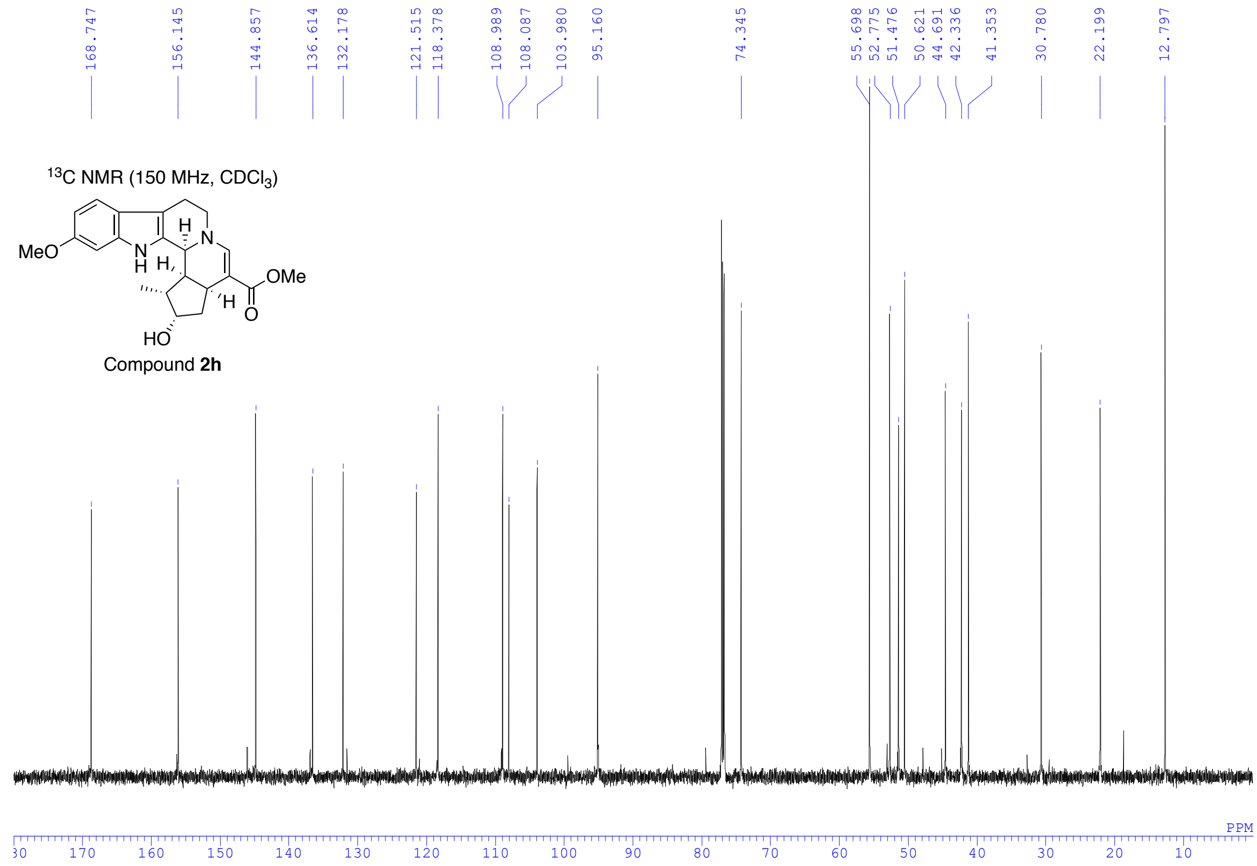

168.747
156.145
144.857
136.614
132.178
121.515
118.378
108.989
108.087
103.980
95.160
74.345
55.698
52.775
51.476
50.621
44.691
42.336
41.353
30.780
22.199
12.797
13C NMR (150 MHz, CDCl3)
MeO
H
N
H
OMe
O
HO
Compound 2h
PPM
170
160
150
140
130
120
110
100
90
80
70
60
50
40
30
20
10

^{13}C NMR (150 MHz, $CDCl_3$)

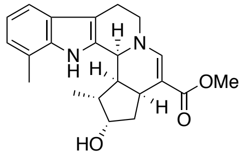

Compound **2i**

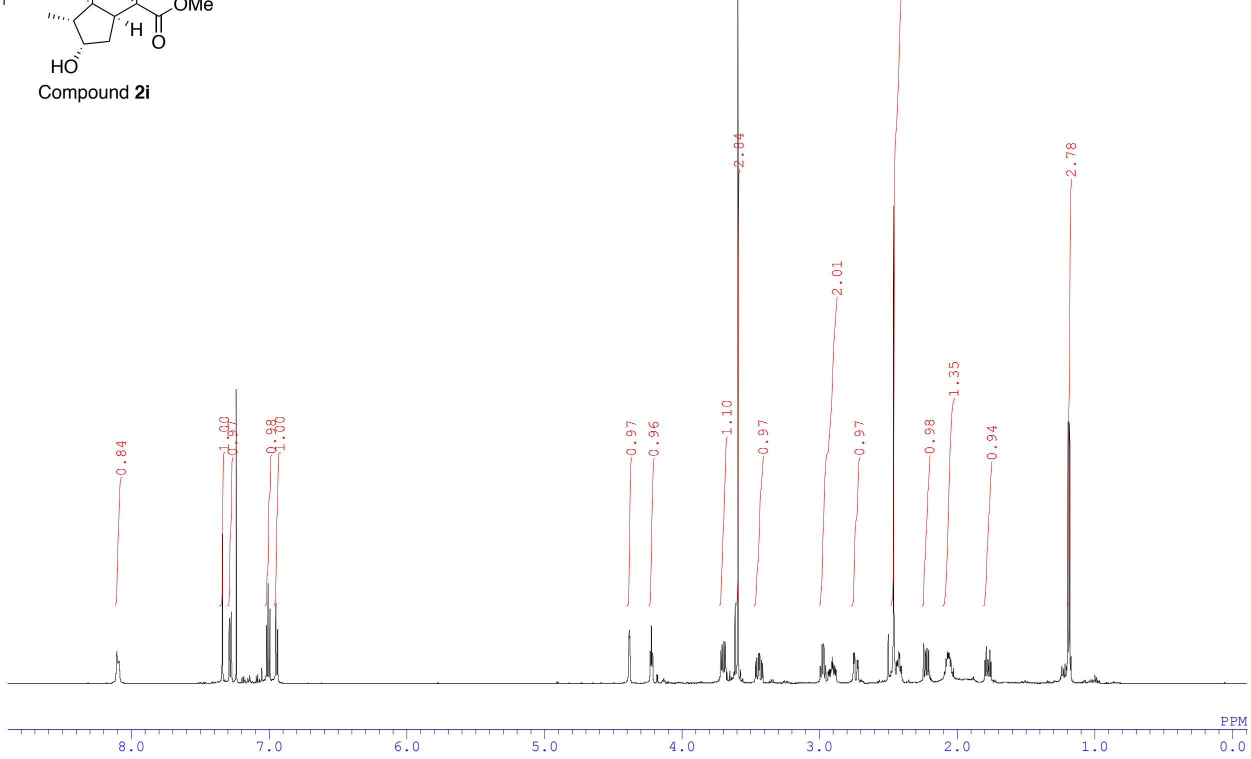

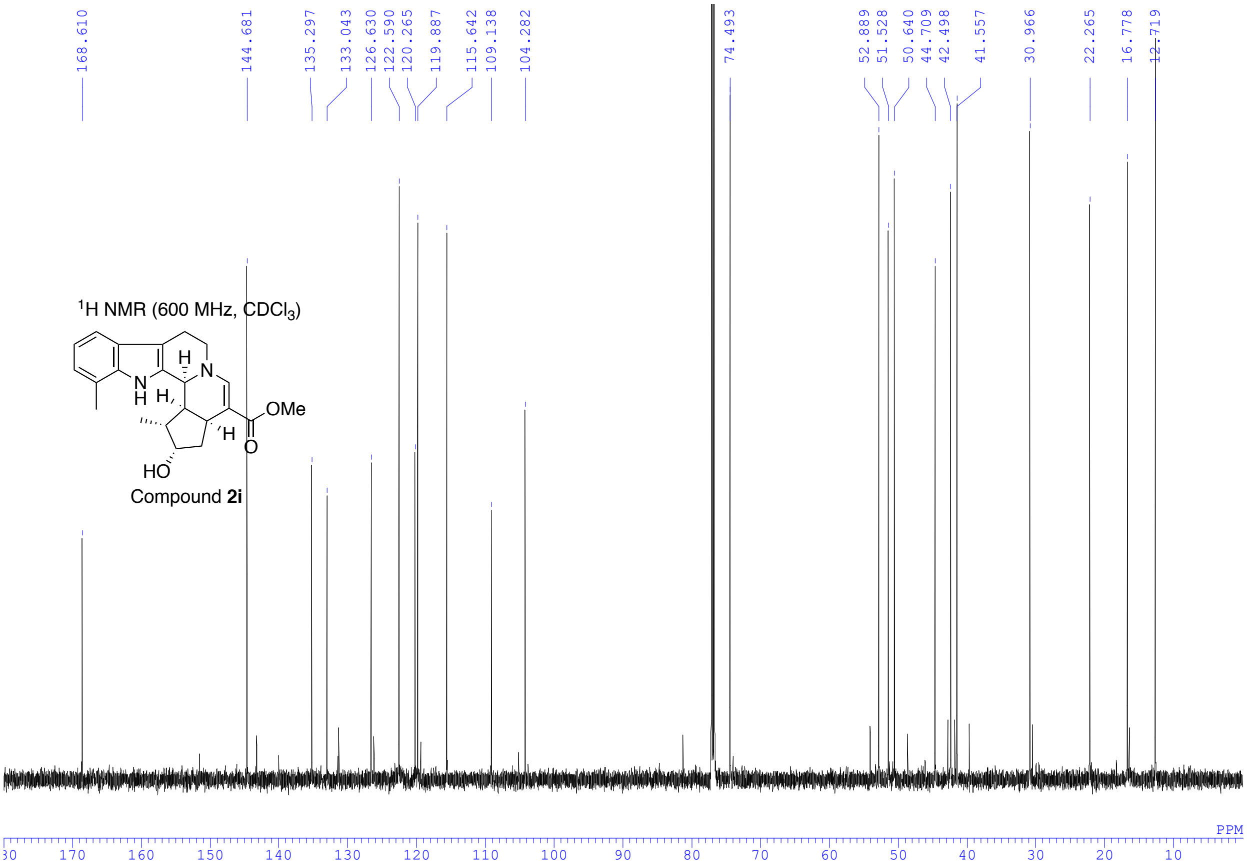

^{1}H NMR (600 MHz, $CDCl_3$)

Compound **2i**

^{1}H NMR (600 MHz, methanol-d_4)

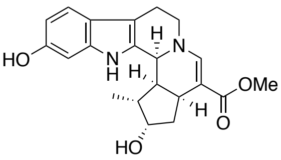

Compound **2j**

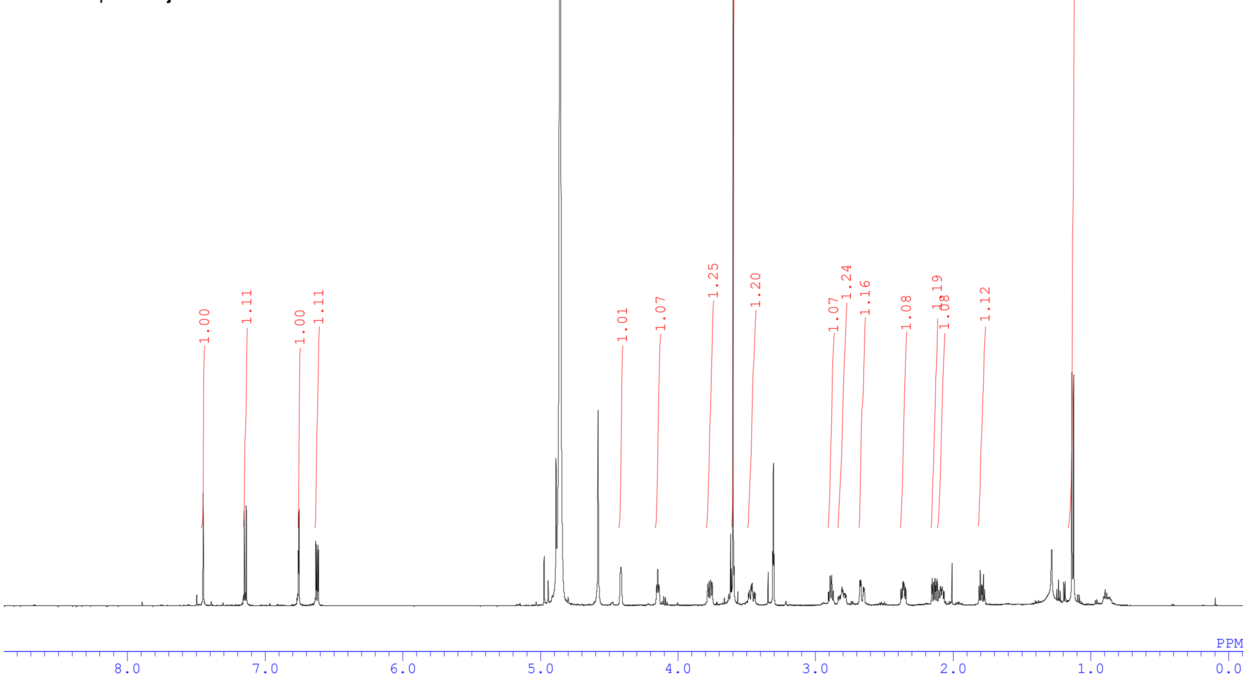

^{13}C NMR (150 MHz, methanol-d_4)

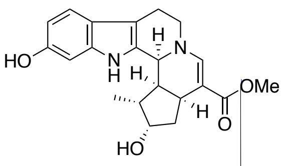

Compound **2j**

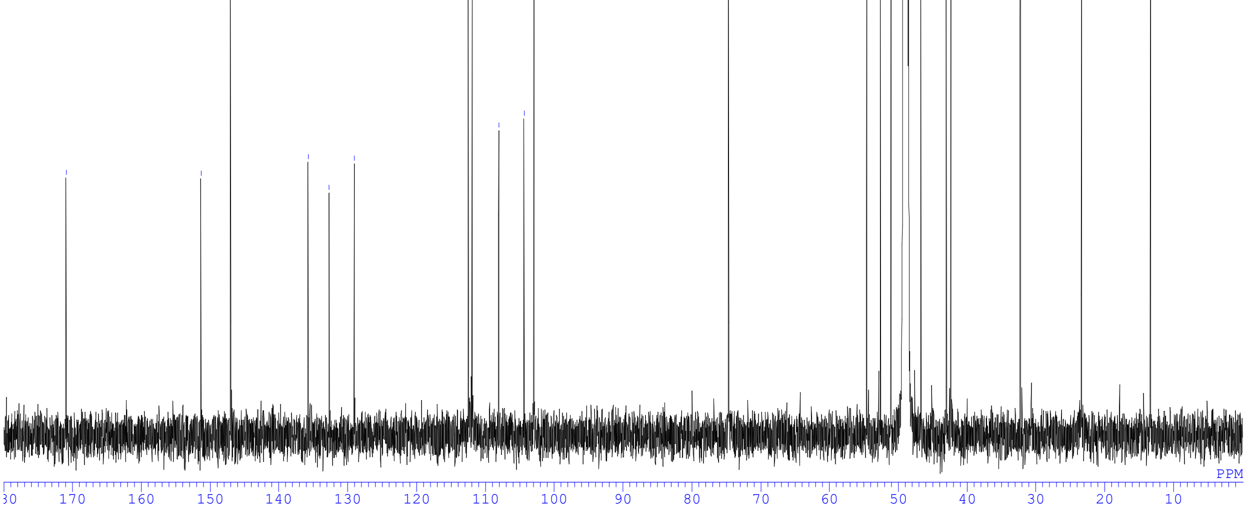

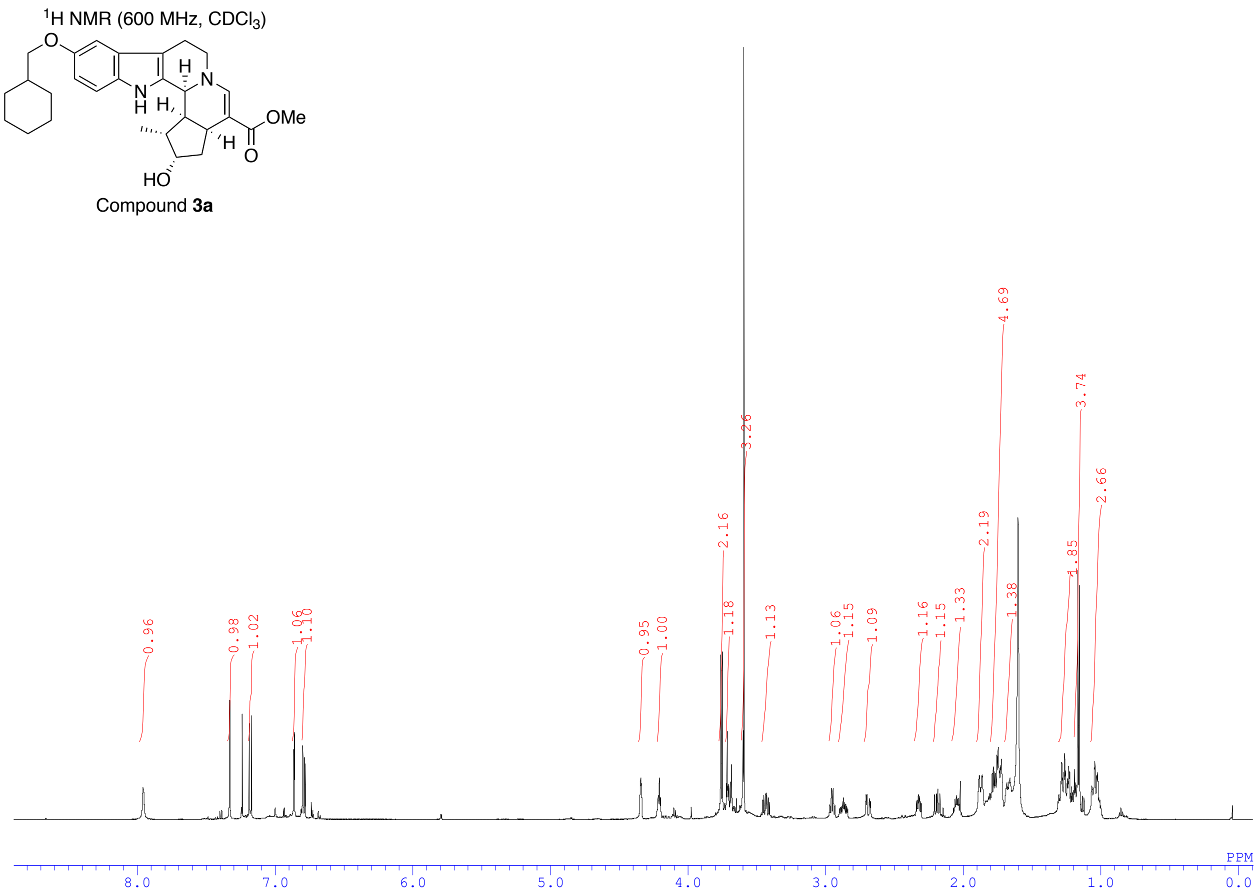
^1H NMR (600 MHz, $CDCl_3$)
OMe
HO
Compound **3a**
0.96
0.98
1.02
1.06
1.10
0.95
1.00
2.16
1.18
3.26
1.13
1.06
1.15
1.09
1.16
1.15
1.33
2.19
4.69
1.38
1.85
3.74
2.66
PPM
8.0
7.0
6.0
5.0
4.0
3.0
2.0
1.0
0.0

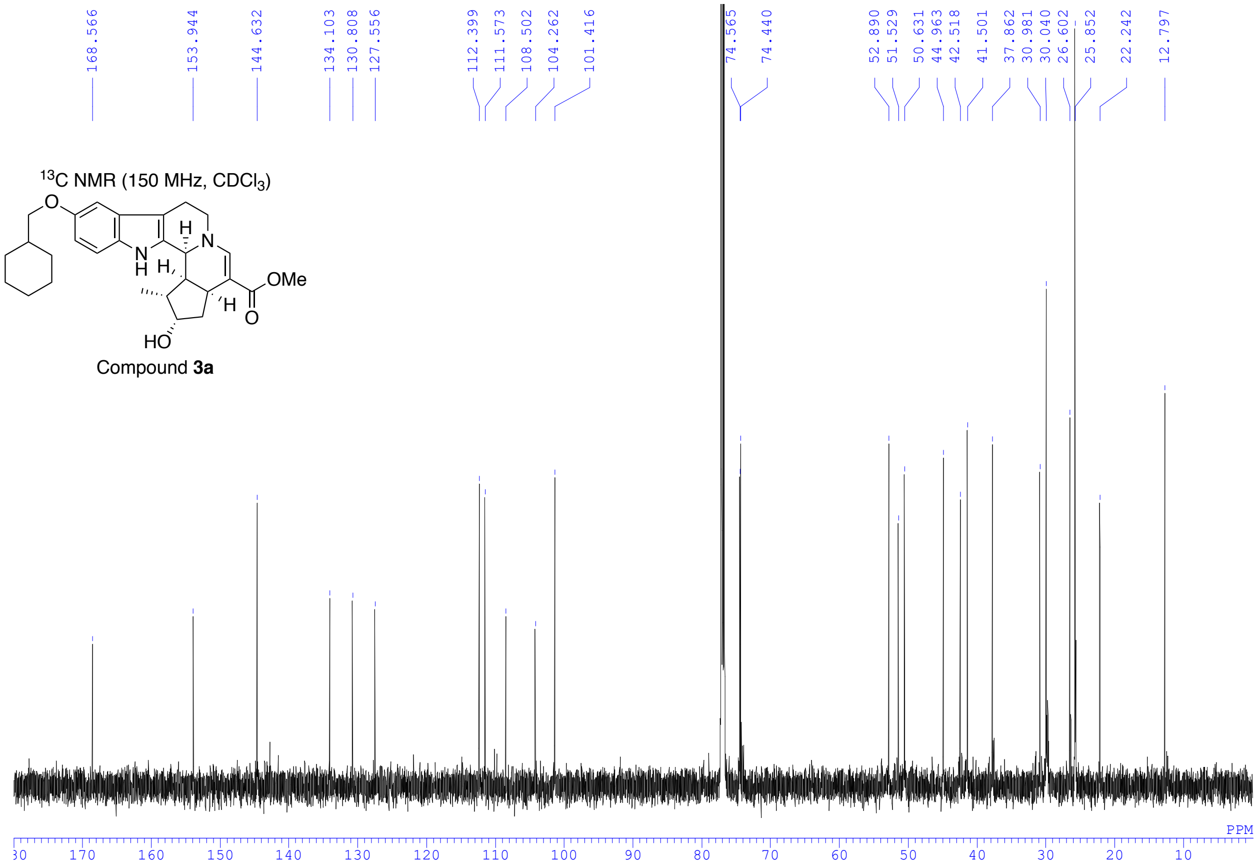
168.566
153.944
144.632
134.103
130.808
127.556
112.399
111.573
108.502
104.262
101.416
74.565
74.440
52.890
51.529
50.631
44.963
42.518
41.501
37.862
30.981
30.040
26.602
25.852
22.242
12.797
^{13}C NMR (150 MHz, $CDCl_3$)
OMe
HO
Compound **3a**
PPM
170
160
150
140
130
120
110
100
90
80
70
60
50
40
30
20
10

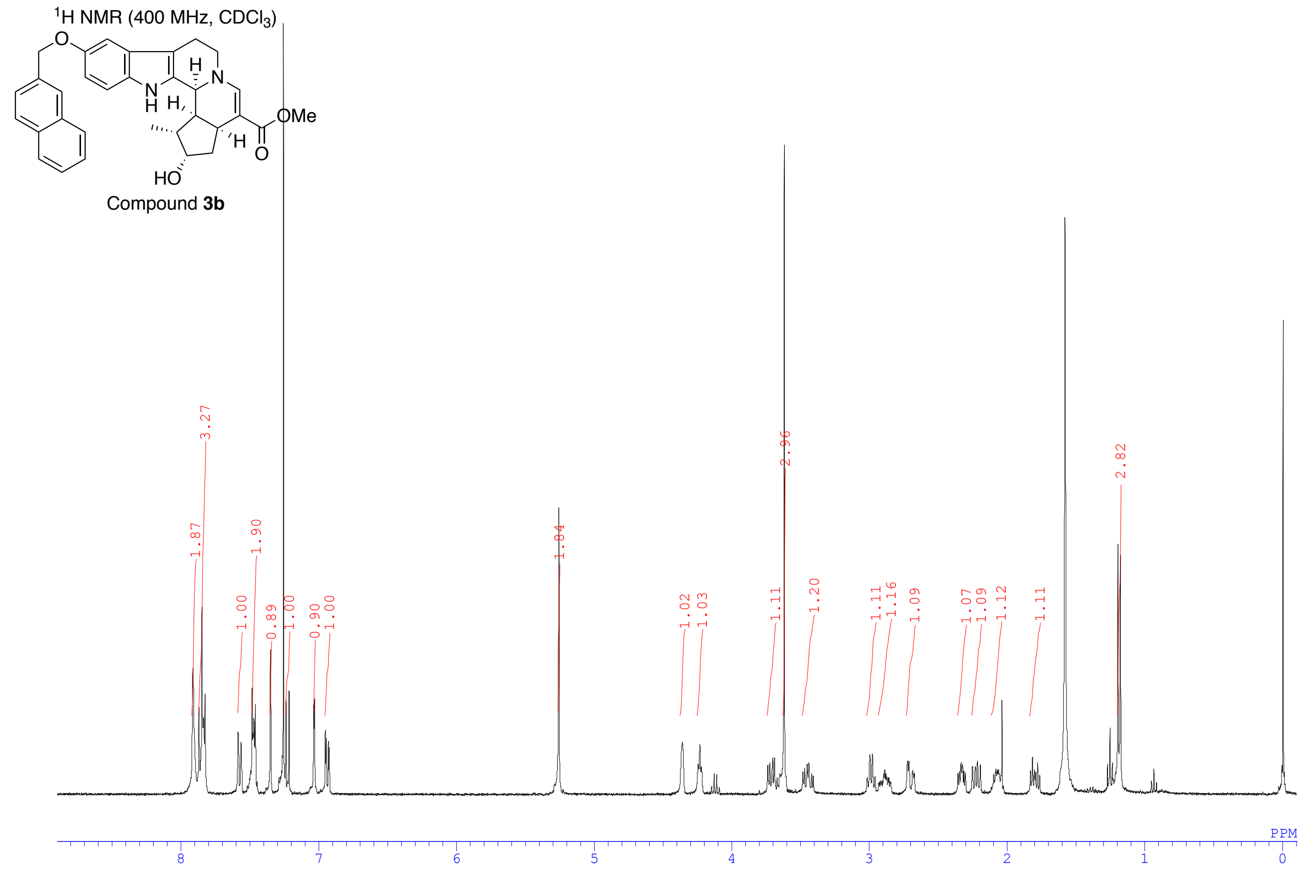

^{1}H NMR (400 MHz, CDCl$_3$)
Compound **3b**
PPM

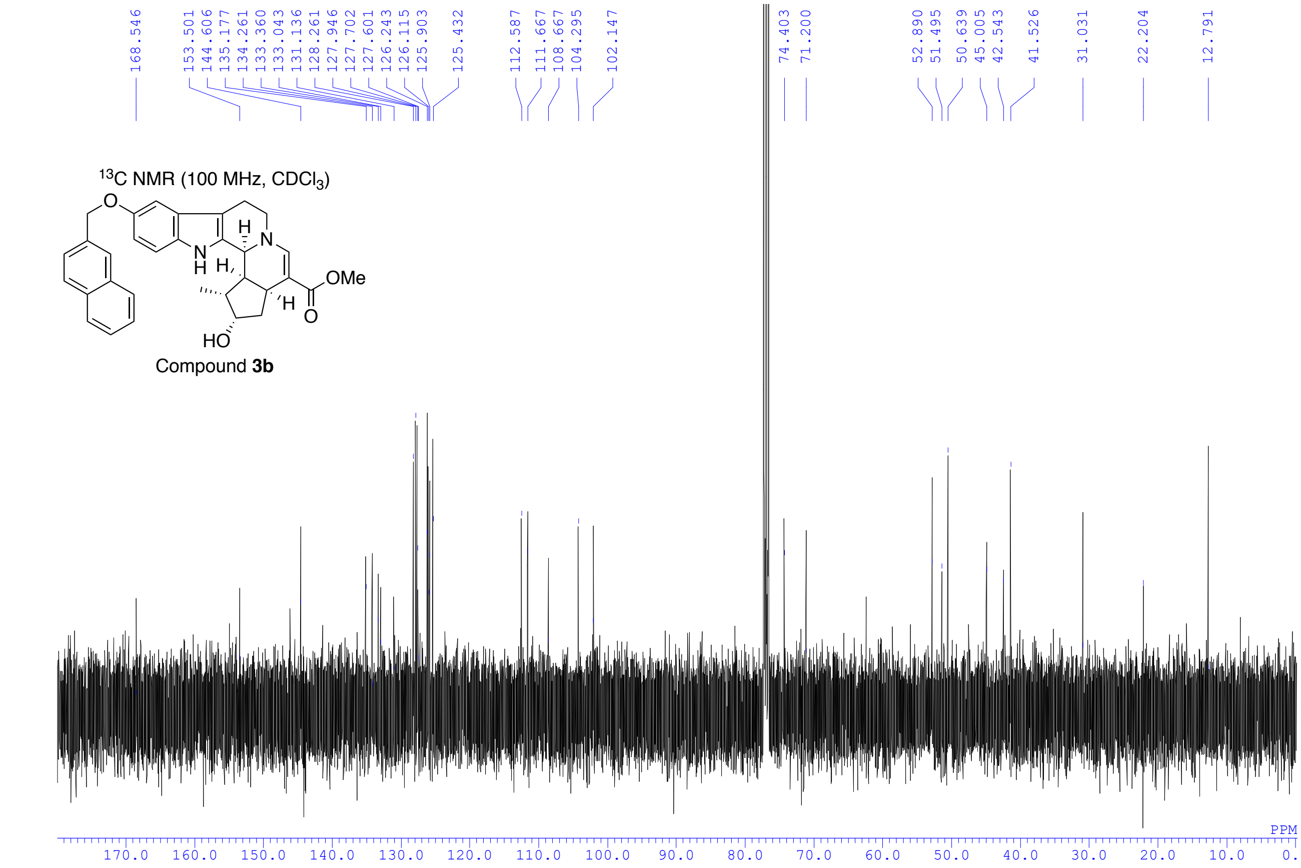

^{13}C NMR (100 MHz, CDCl$_3$)
Compound **3b**
168.546
153.501
144.606
135.177
134.261
133.360
133.043
131.136
128.261
127.946
127.702
127.601
126.243
126.115
125.903
125.432
112.587
111.667
108.667
104.295
102.147
74.403
71.200
52.890
51.495
50.639
45.005
42.543
41.526
31.031
22.204
12.791
PPM

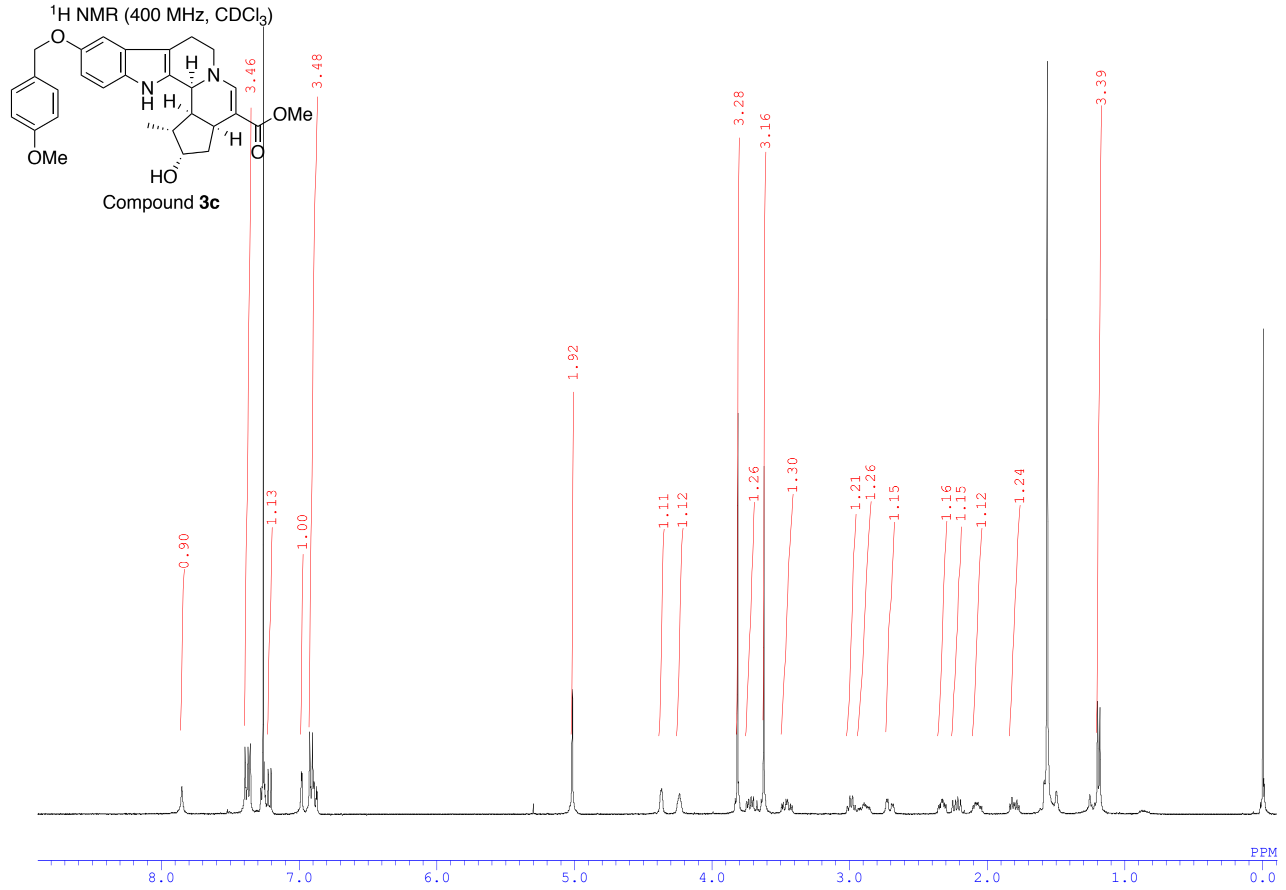

^{1}H NMR (400 MHz, CDCl$_3$)
OMe
HO
Compound **3c**
0.90
3.46
1.13
1.00
3.48
1.92
1.11
1.12
3.28
1.26
3.16
1.30
1.21
1.26
1.15
1.16
1.15
1.12
1.24
3.39
PPM
8.0
7.0
6.0
5.0
4.0
3.0
2.0
1.0
0.0

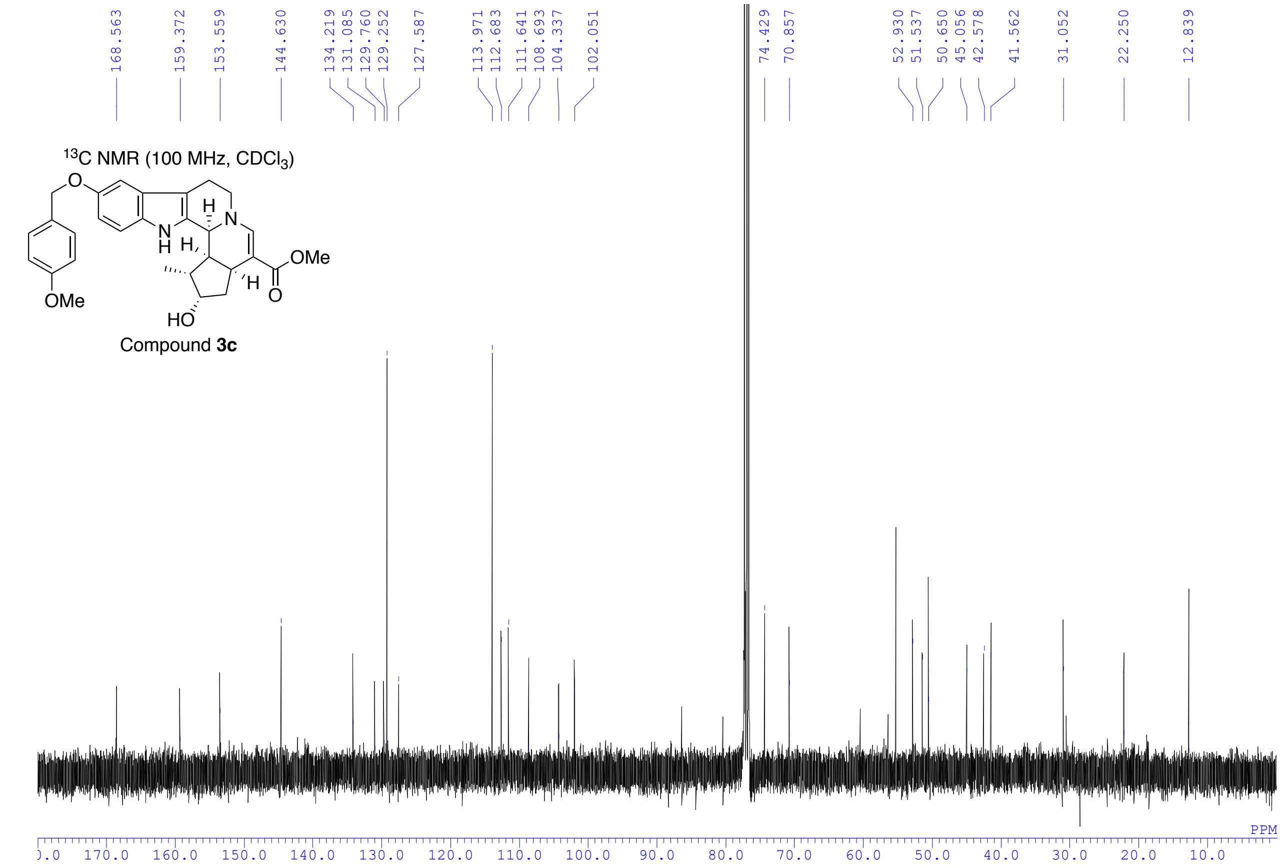

168.563
159.372
153.559
144.630
134.219
131.085
129.760
129.252
127.587
113.971
112.683
111.641
108.693
104.337
102.051
74.429
70.857
52.930
51.537
50.650
45.056
42.578
41.562
31.052
22.250
12.839
^{13}C NMR (100 MHz, CDCl$_3$)
OMe
HO
Compound **3c**
PPM
170.0
160.0
150.0
140.0
130.0
120.0
110.0
100.0
90.0
80.0
70.0
60.0
50.0
40.0
30.0
20.0
10.0

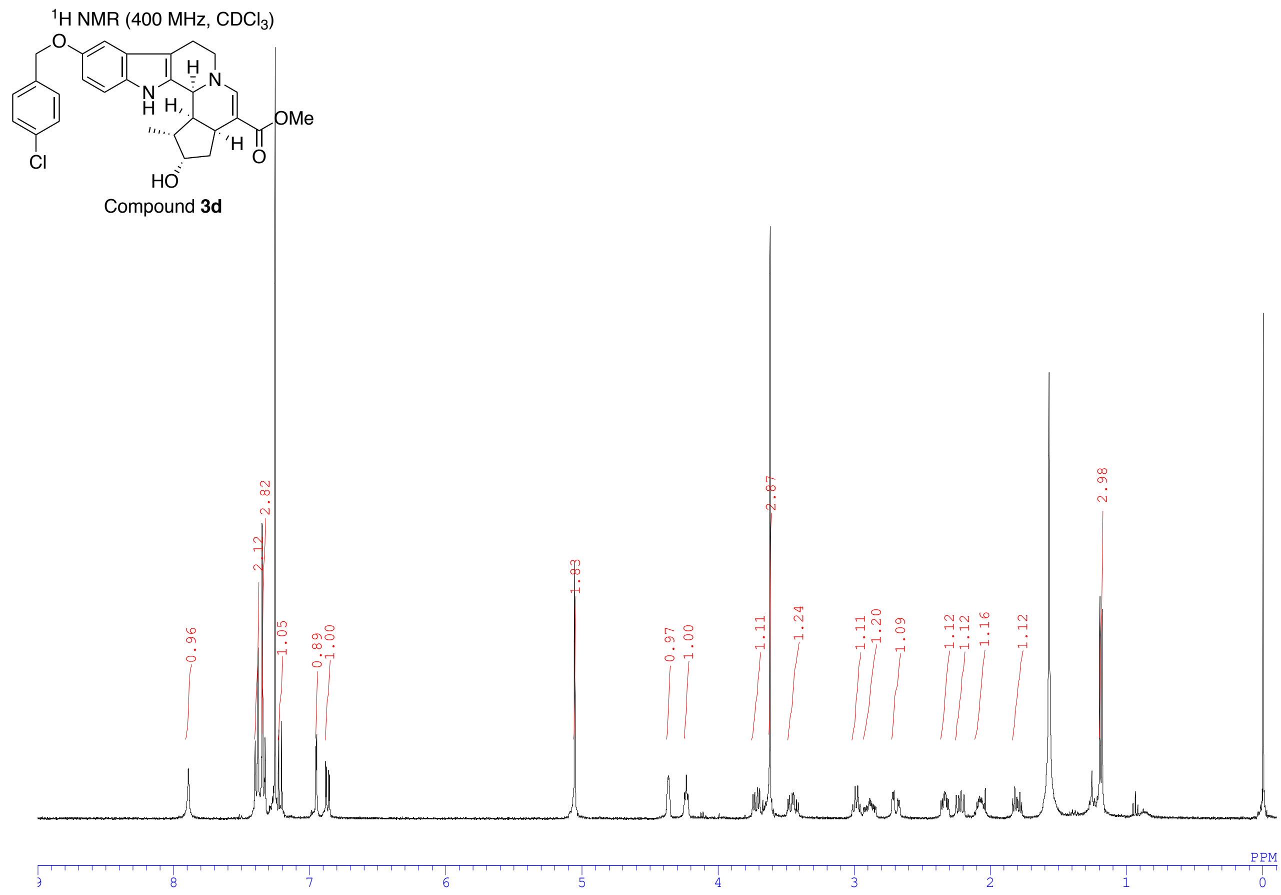

1H NMR (400 MHz, CDCl3)
OMe
HO
Cl
Compound 3d
0.96
2.12
2.82
1.05
0.89
1.00
1.83
0.97
1.00
1.11
2.87
1.24
1.11
1.20
1.09
1.12
1.12
1.16
1.12
2.98
PPM
8
7
6
5
4
3
2
1
0

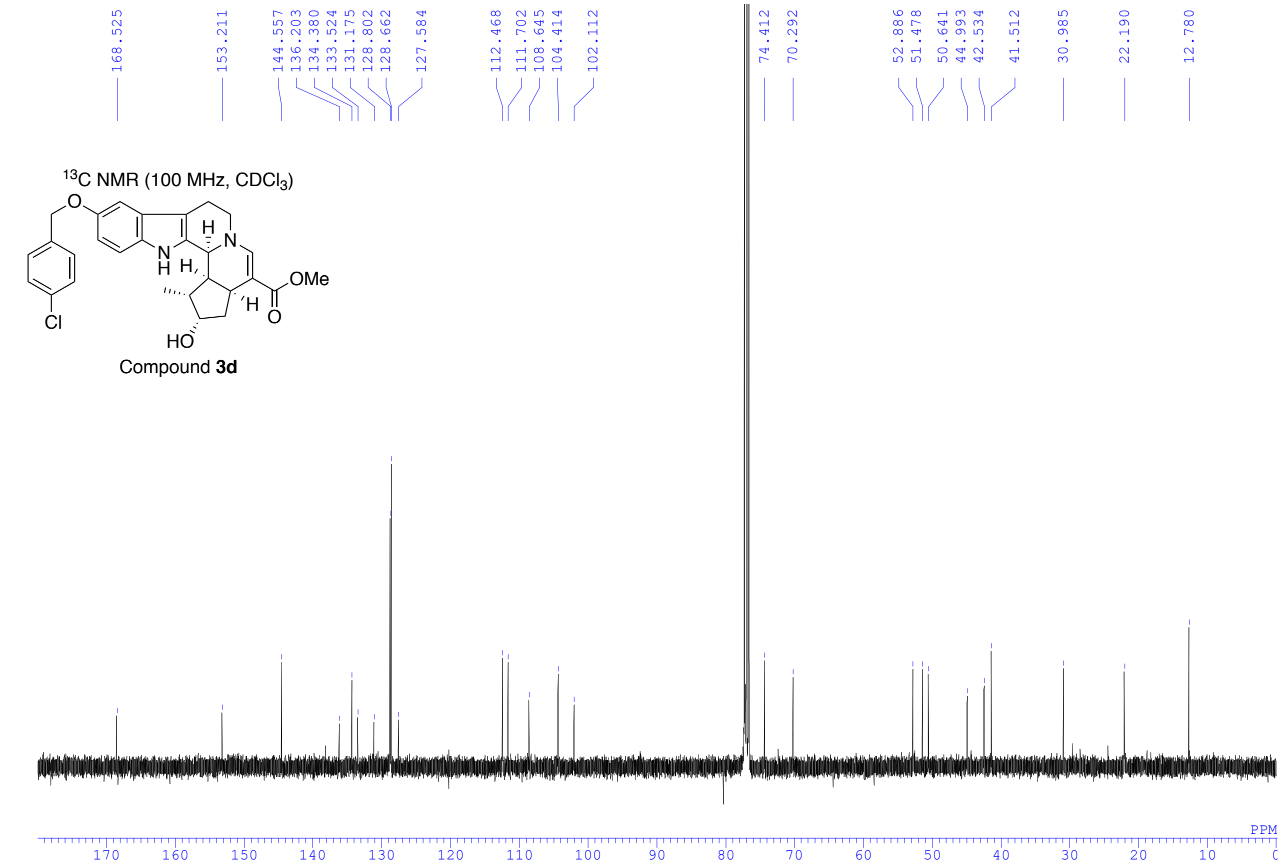

168.525
153.211
144.557
136.203
134.380
133.524
131.175
128.802
128.662
127.584
112.468
111.702
108.645
104.414
102.112
74.412
70.292
52.886
51.478
50.641
44.993
42.534
41.512
30.985
22.190
12.780
13C NMR (100 MHz, CDCl3)
OMe
HO
Cl
Compound 3d
PPM
170
160
150
140
130
120
110
100
90
80
70
60
50
40
30
20
10

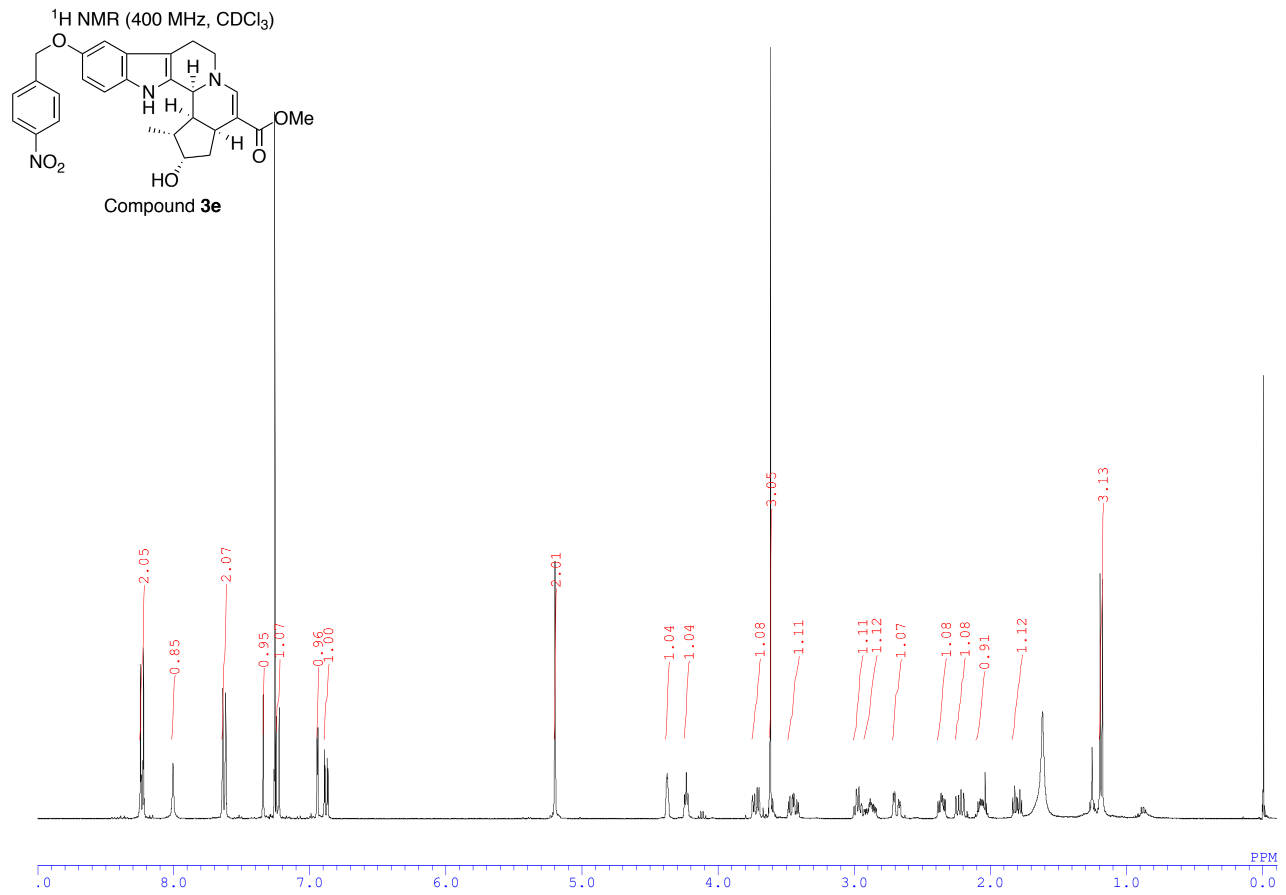
^{1}H NMR (400 MHz, CDCl$_3$)
Compound **3e**
OMe
HO
NO_2
PPM
8.0 7.0 6.0 5.0 4.0 3.0 2.0 1.0 0.0

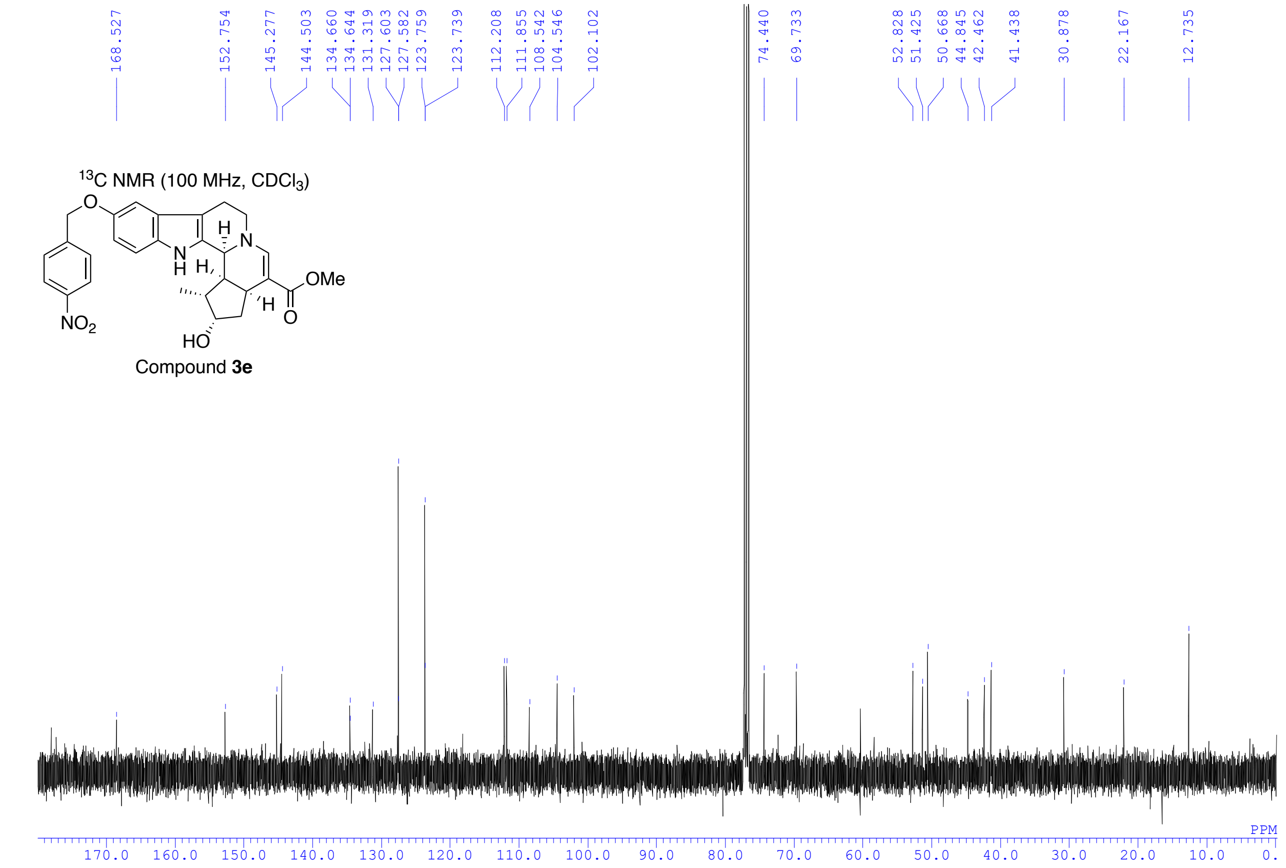
^{13}C NMR (100 MHz, CDCl$_3$)
Compound **3e**
168.527
152.754
145.277
144.503
134.660
134.644
131.319
127.603
127.582
123.759
123.739
112.208
111.855
108.542
104.546
102.102
74.440
69.733
52.828
51.425
50.668
44.845
42.462
41.438
30.878
22.167
12.735
PPM
170.0 160.0 150.0 140.0 130.0 120.0 110.0 100.0 90.0 80.0 70.0 60.0 50.0 40.0 30.0 20.0 10.0 0.

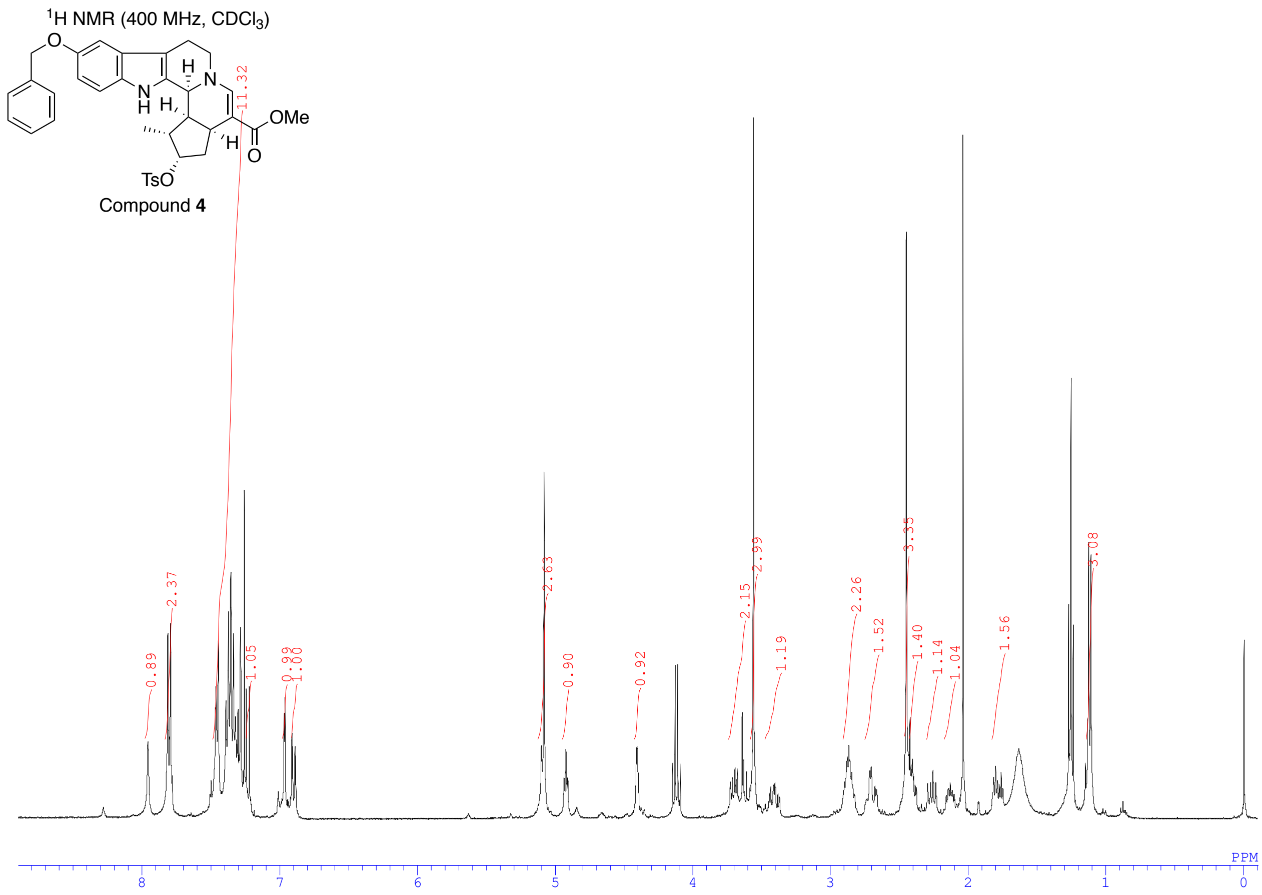

1H NMR (400 MHz, CDCl3)
OMe
TsO
Compound 4
PPM

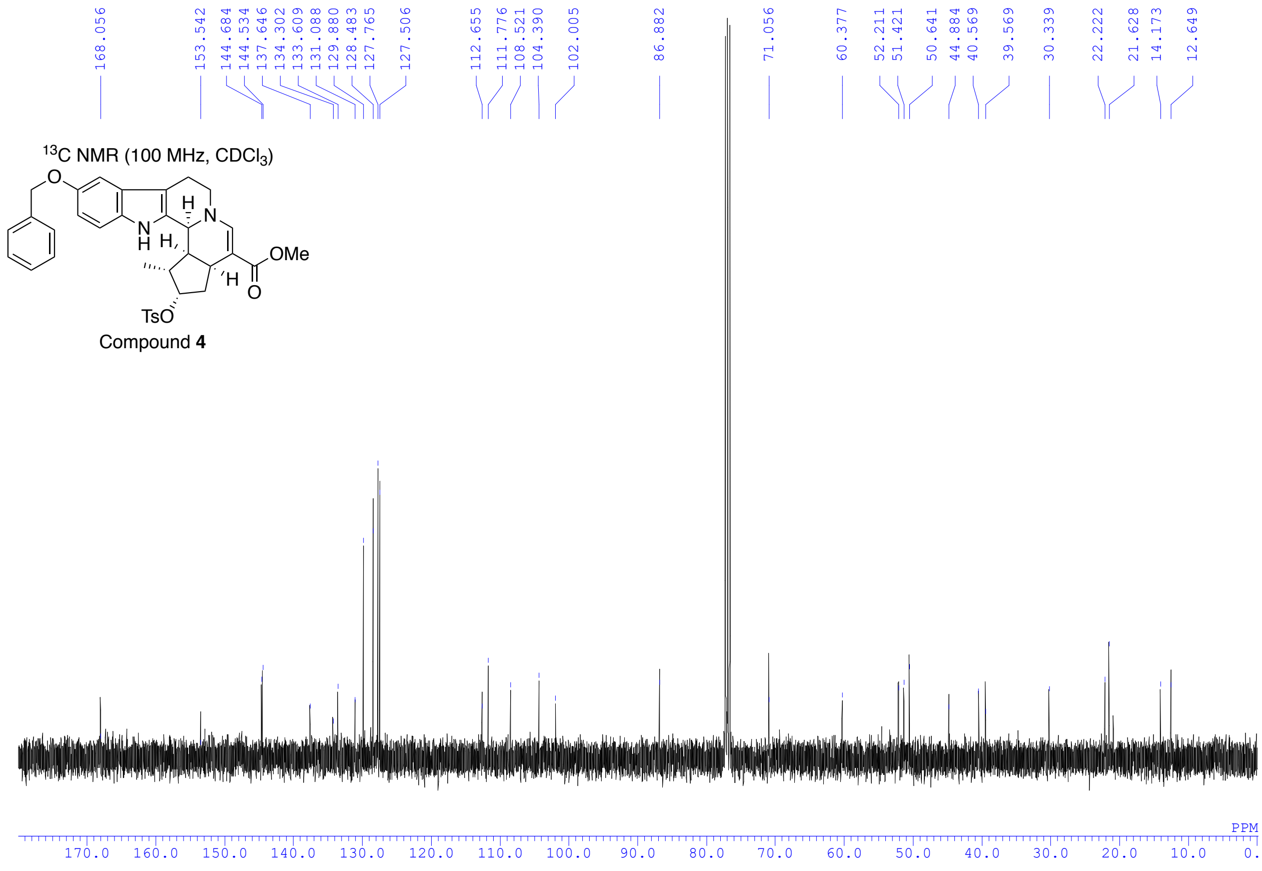

13C NMR (100 MHz, CDCl3)
OMe
TsO
Compound 4
168.056
153.542
144.684
144.534
137.646
134.302
133.609
131.088
129.880
128.483
127.765
127.506
112.655
111.776
108.521
104.390
102.005
86.882
71.056
60.377
52.211
51.421
50.641
44.884
40.569
39.569
30.339
22.222
21.628
14.173
12.649
PPM

^{1}H NMR (600 MHz, $CDCl_3$)

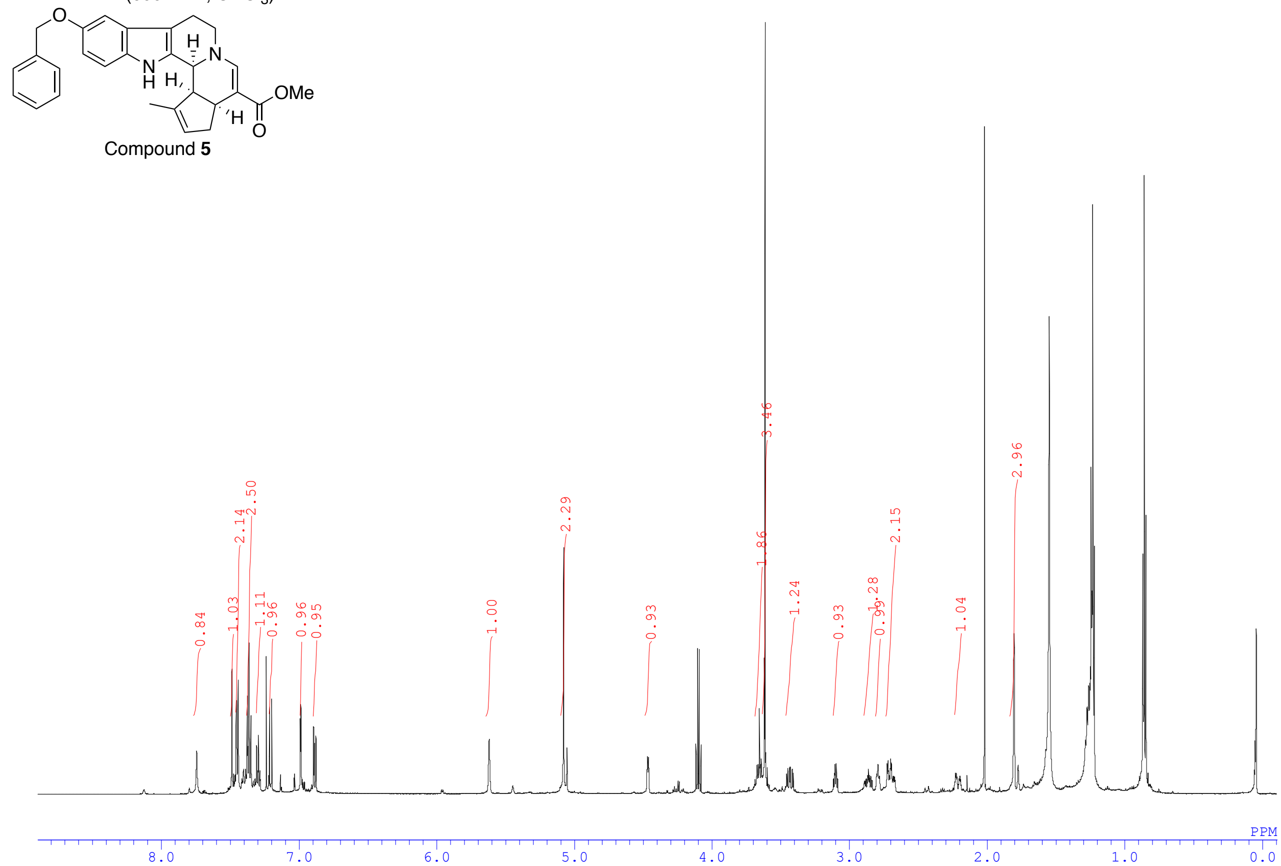

Compound **5**

^{13}C NMR (150 MHz, $CDCl_3$)

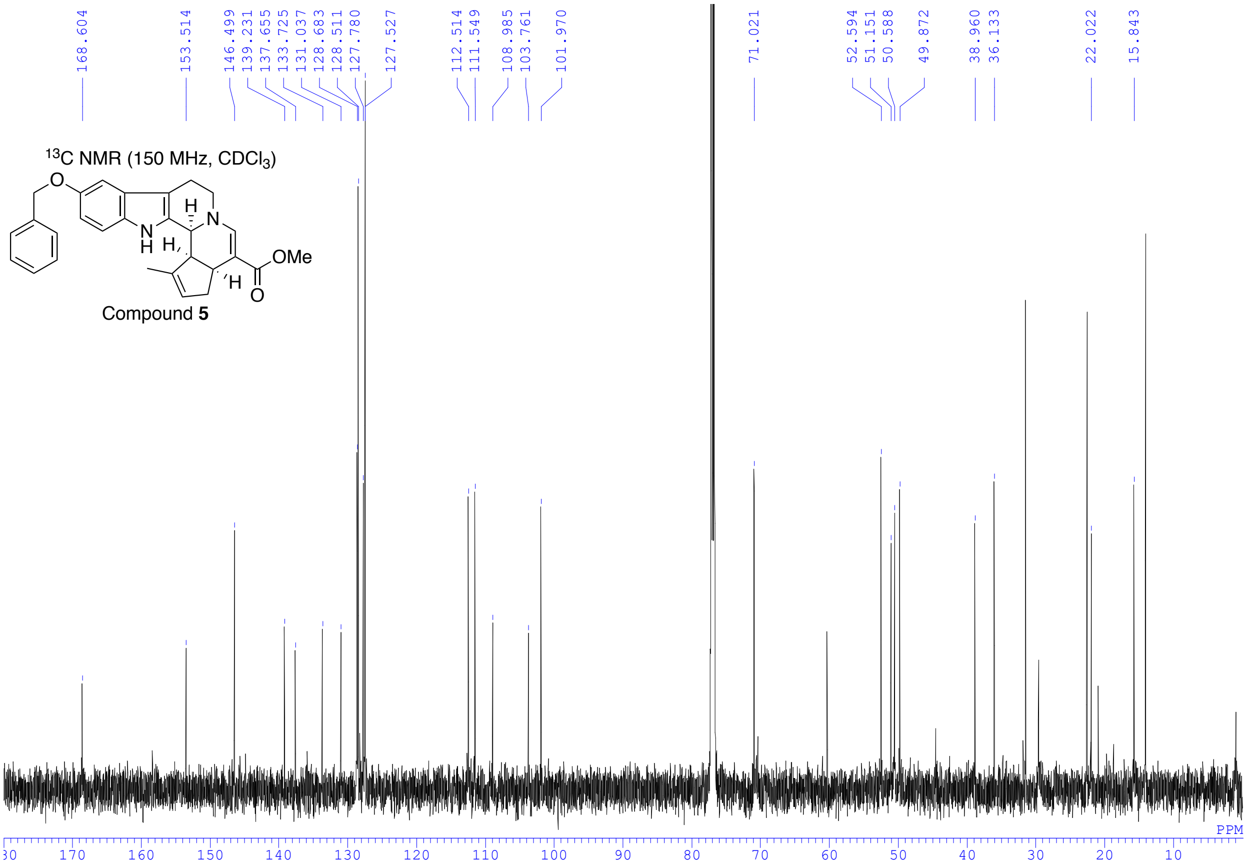

Compound **5**

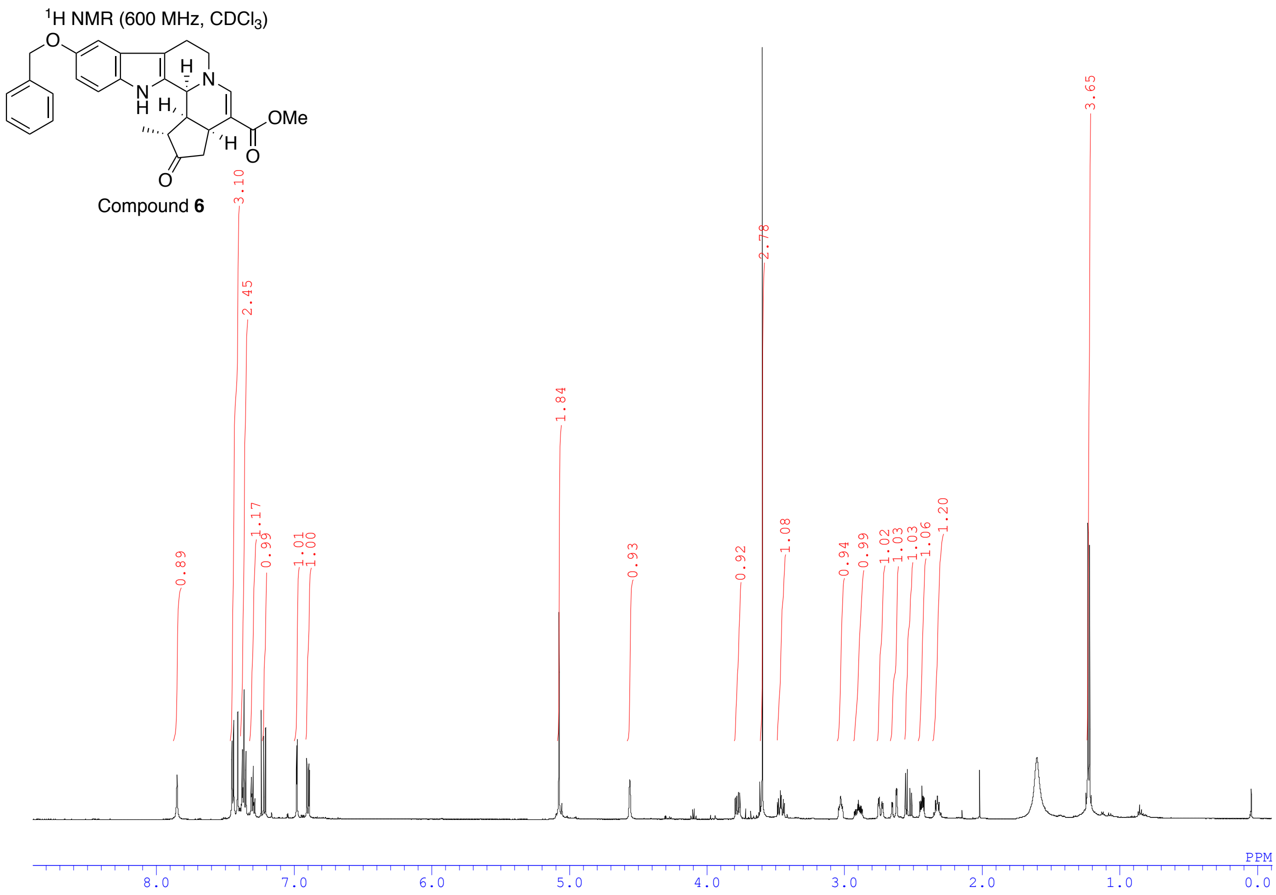
1H NMR (600 MHz, CDCl3)
OMe
Compound 6
3.65
2.78
3.10
2.45
1.84
PPM
8.0
7.0
6.0
5.0
4.0
3.0
2.0
1.0
0.0

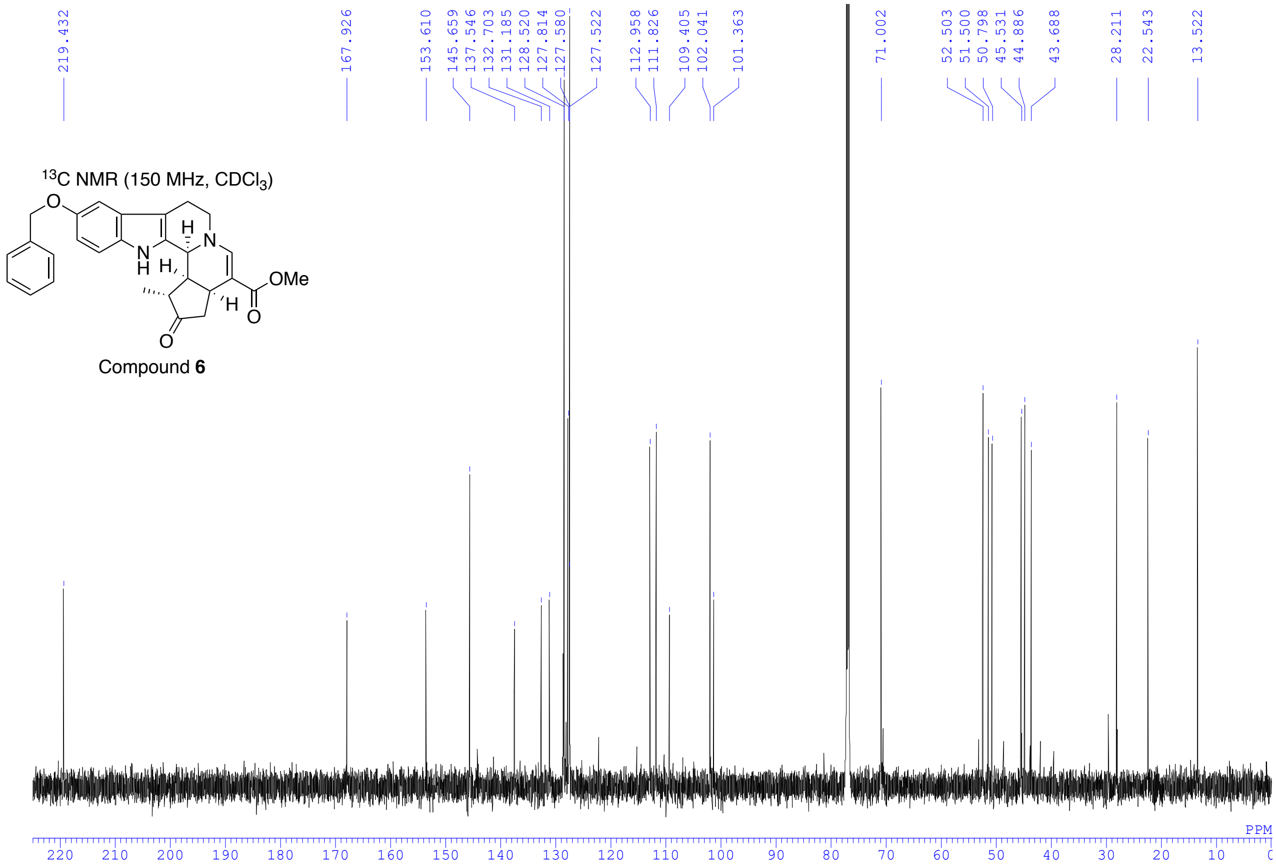
13C NMR (150 MHz, CDCl3)
OMe
Compound 6
219.432
167.926
153.610
145.659
137.546
132.703
131.185
128.520
127.814
127.580
127.522
112.958
111.826
109.405
102.041
101.363
71.002
52.503
51.500
50.798
45.531
44.886
43.688
28.211
22.543
13.522
PPM

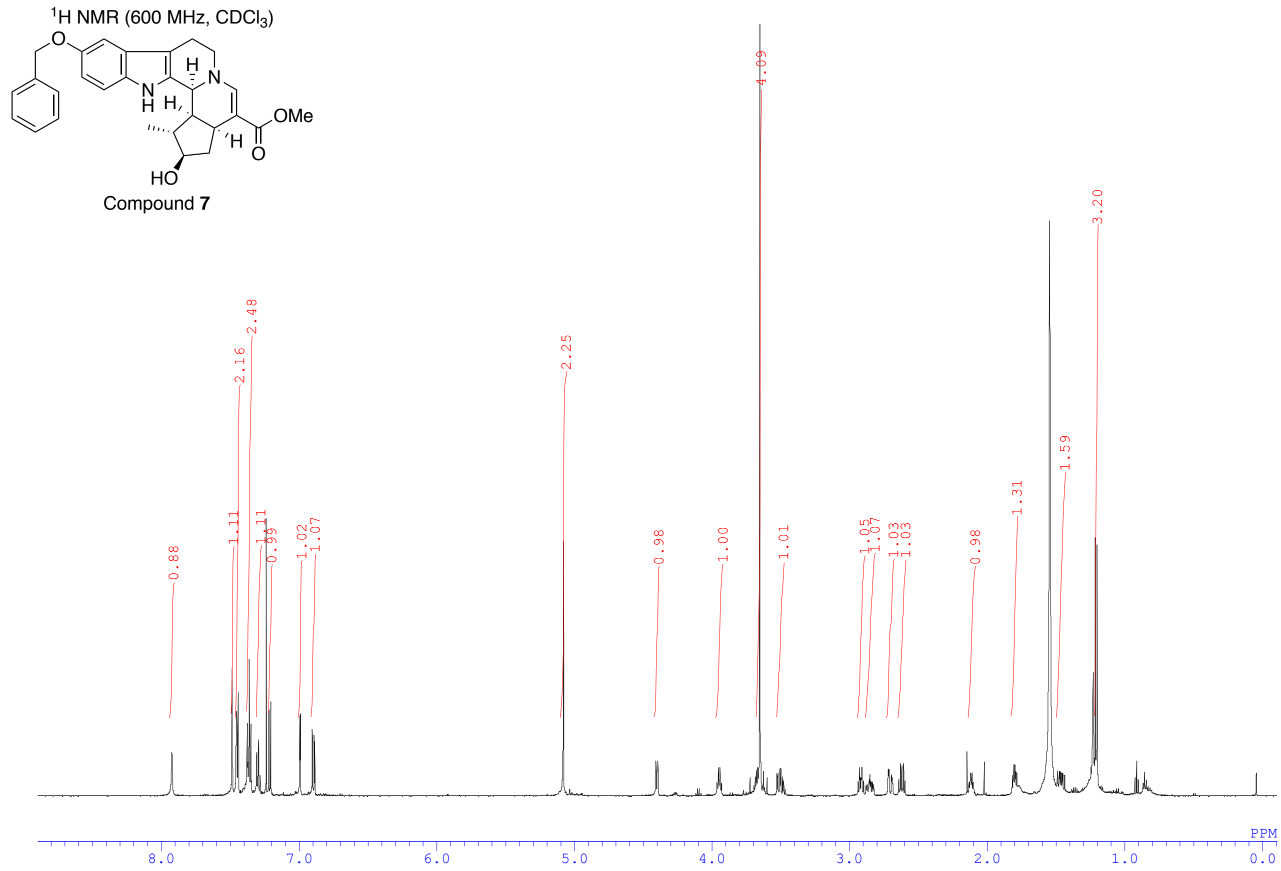
1H NMR (600 MHz, CDCl3)
OMe
HO
Compound 7
0.88
1.11
2.16
2.48
1.11
0.99
1.02
1.07
2.25
0.98
1.00
4.09
1.01
1.05
1.07
1.03
1.03
0.98
1.31
1.59
3.20
PPM
8.0
7.0
6.0
5.0
4.0
3.0
2.0
1.0
0.0

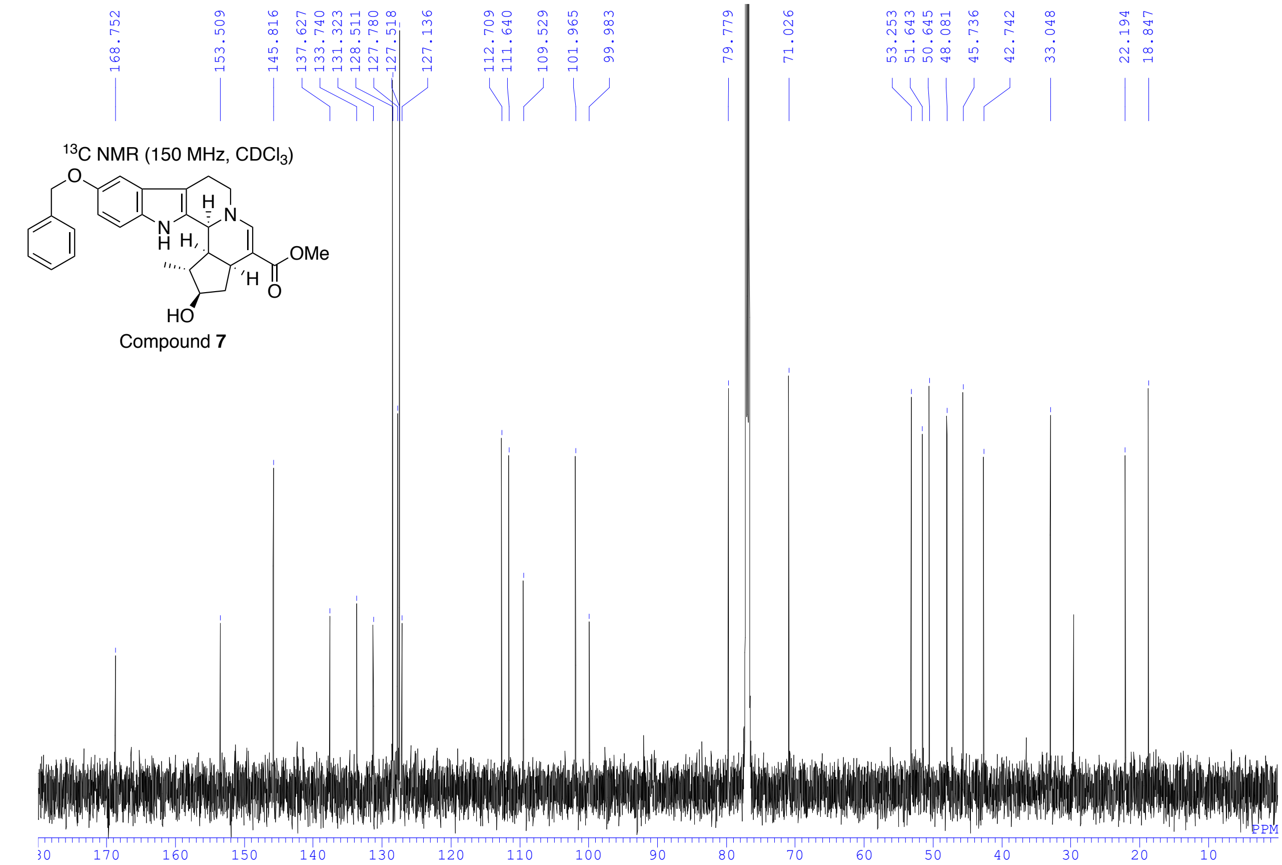
168.752
153.509
145.816
137.627
133.740
131.323
128.511
127.780
127.518
127.136
112.709
111.640
109.529
101.965
99.983
79.779
71.026
53.253
51.643
50.645
48.081
45.736
42.742
33.048
22.194
18.847
13C NMR (150 MHz, CDCl3)
OMe
HO
Compound 7
PPM
170
160
150
140
130
120
110
100
90
80
70
60
50
40
30
20
10

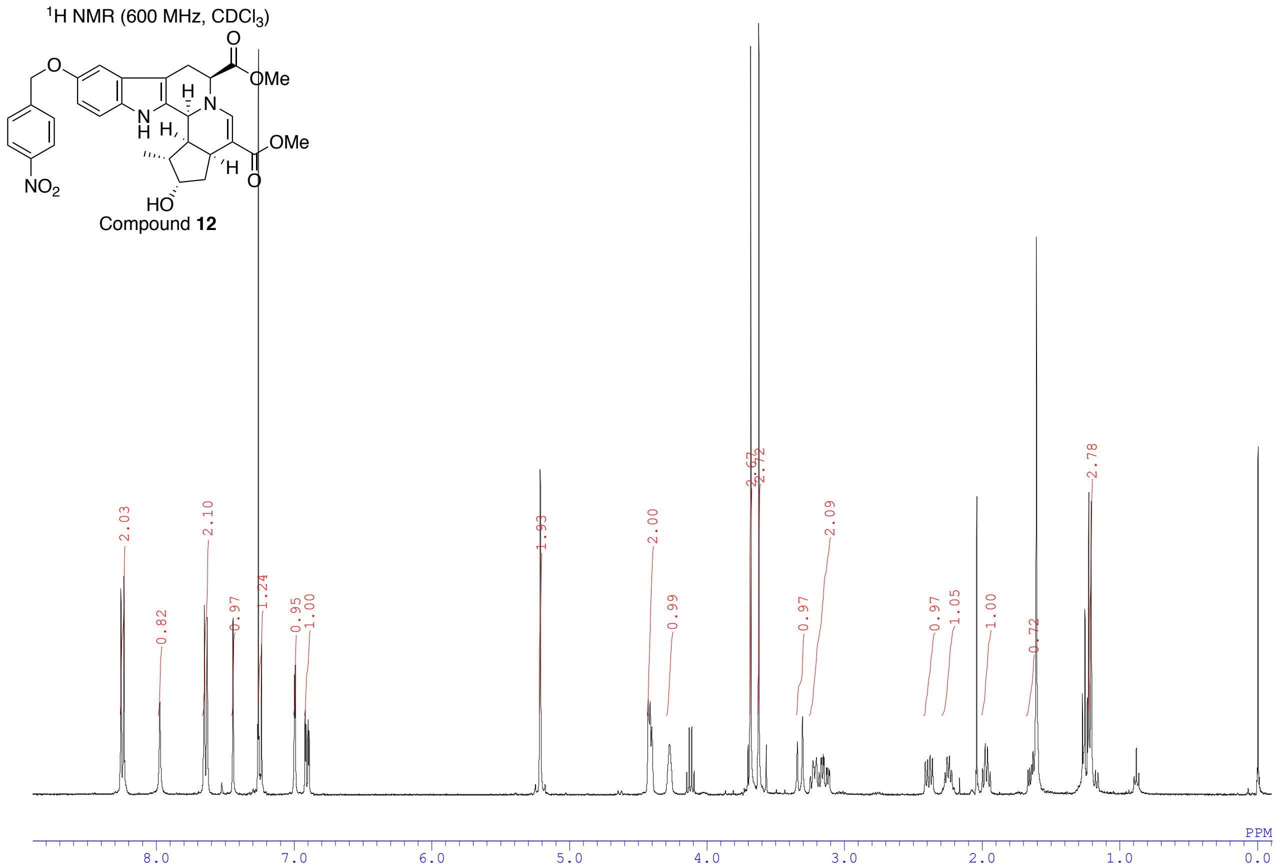

1H NMR (600 MHz, CDCl3)
OMe
OMe
NO2
HO
Compound 12
2.03
0.82
2.10
0.97
1.24
0.95
1.00
1.93
2.00
0.99
2.67
2.72
0.97
2.09
0.97
1.05
1.00
0.72
2.78
PPM
8.0
7.0
6.0
5.0
4.0
3.0
2.0
1.0
0.0

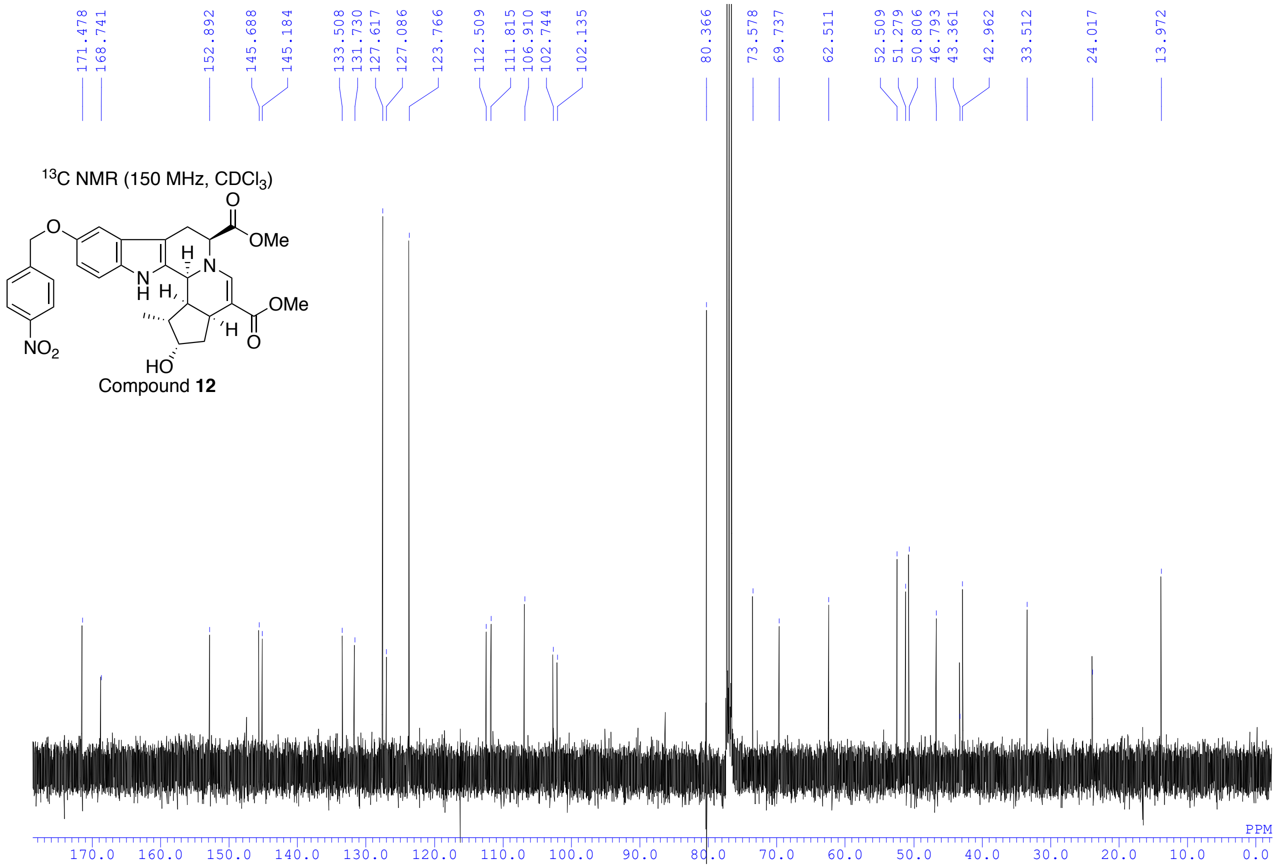

171.478
168.741
152.892
145.688
145.184
133.508
131.730
127.617
127.086
123.766
112.509
111.815
106.910
102.744
102.135
80.366
73.578
69.737
62.511
52.509
51.279
50.806
46.793
43.361
42.962
33.512
24.017
13.972
13C NMR (150 MHz, CDCl3)
OMe
OMe
NO2
HO
Compound 12
PPM
170.0
160.0
150.0
140.0
130.0
120.0
110.0
100.0
90.0
80.0
70.0
60.0
50.0
40.0
30.0
20.0
10.0
0.0

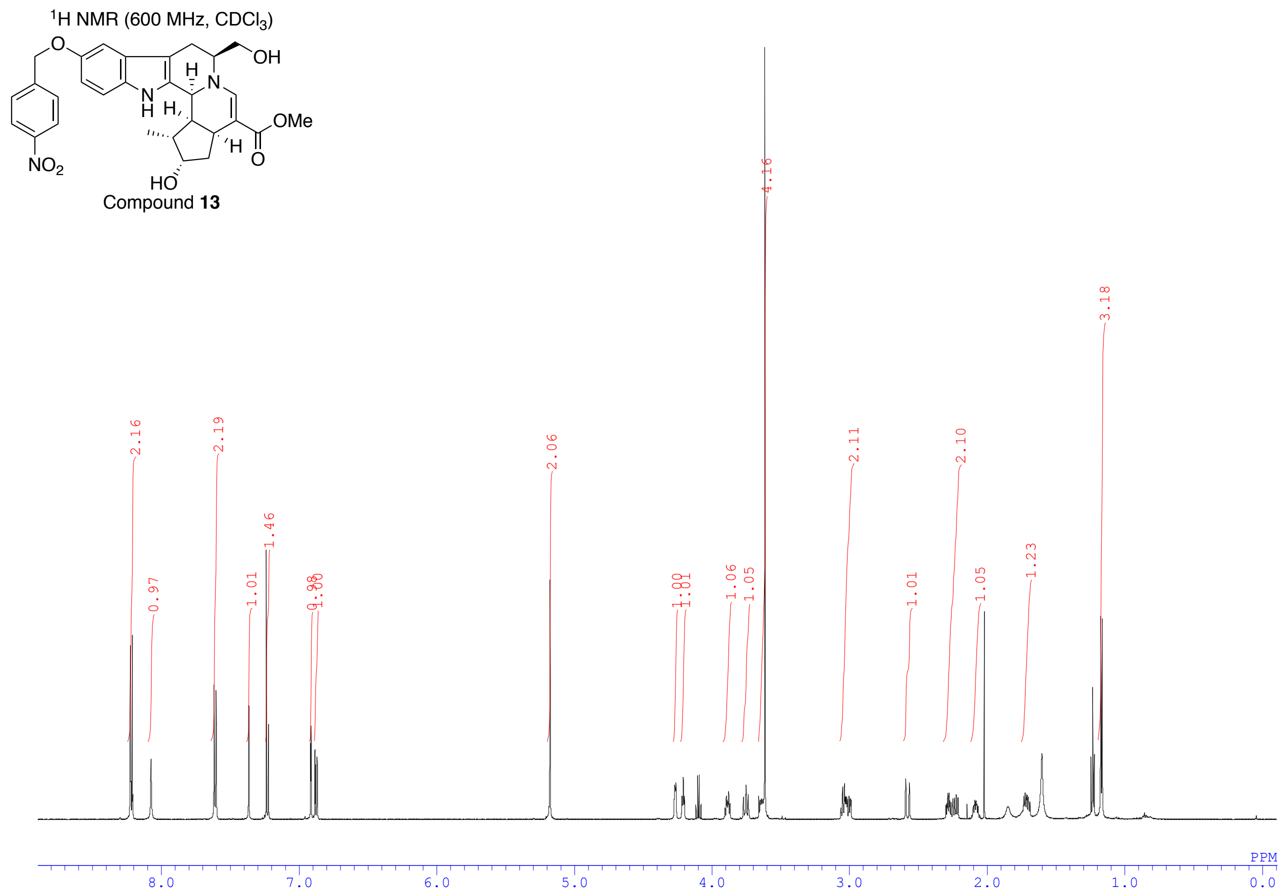

^{1}H NMR (600 MHz, $CDCl_3$)
O
OH
H
N
H H
OMe
H
O
NO_2
HO
Compound **13**
2.16
0.97
2.19
1.01
1.46
0.98
1.00
2.06
1.00
1.01
1.06
1.05
4.16
2.11
1.01
2.10
1.05
1.23
3.18
PPM
8.0
7.0
6.0
5.0
4.0
3.0
2.0
1.0
0.0

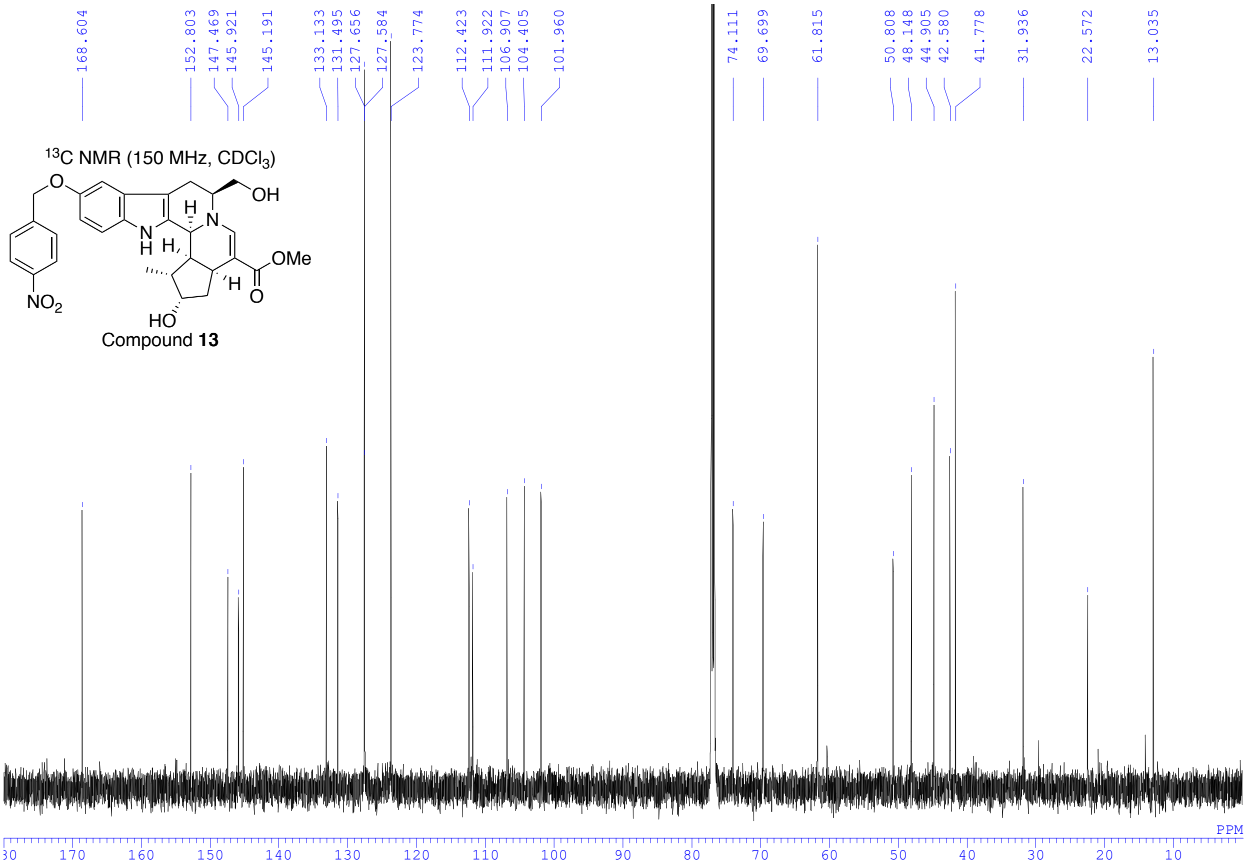

168.604
152.803
147.469
145.921
145.191
133.133
131.495
127.656
127.584
123.774
112.423
111.922
106.907
104.405
101.960
74.111
69.699
61.815
50.808
48.148
44.905
42.580
41.778
31.936
22.572
13.035
^{13}C NMR (150 MHz, $CDCl_3$)
O
OH
H
N
H H
OMe
H
O
NO_2
HO
Compound **13**
PPM
30
170
160
150
140
130
120
110
100
90
80
70
60
50
40
30
20
10
